# Religious celibacy is linked to marriage and wealth transmission practices across different world religions

**DOI:** 10.1038/s41467-026-78153-0

**Published:** 2026-09-29

**Authors:** Gabriel Šaffa, Sara Sherbaji, Ruth Mace

**Affiliations:** Department of Anthropology, University College London, London, UK

**Keywords:** Anthropology, Cultural evolution

## Abstract

Lifelong religious celibacy may seem to be an evolutionary puzzle. However, it is possible that where siblings compete for parental resources, families can benefit from having some offspring become celibate monks or nuns. Here, we test whether celibacy is more likely to be found in societies with substantial marriage transactions, lower levels of polygyny, and heritable wealth, especially land. Additionally, we predict male celibacy to be linked to polygyny and bride-wealth, and female celibacy to monogamy or polyandry and to dowry. Using Bayesian phylogenetic multilevel models on a cross-cultural sample of 352 societies, we find that celibacy occurs mainly in monogamous societies with heritable wealth, and male and female celibacy are more common where marriage payments are higher for the celibate sex. Buddhism and Christianity are especially likely to institutionalize celibacy, suggesting that the emergence of celibate religious institutions reflects common responses to similar socio-ecological conditions across cultures.

## Abstract (web summary)

Lifelong religious celibacy has been linked to sibling competition over parental wealth. Here, the authors show that celibacy is linked to marriage patterns, and its prevalence among men and women is related to which sex faces higher marriage payments.

## Introduction

Lifelong religious celibacy (hereafter celibacy) is often presented as an evolutionary puzzle: why should anyone forego reproduction^1,2^? This seemingly maladaptive behavior is often explained as the result of an evolutionary mismatch or as a by-product of adaptation. In contrast, recent studies have suggested that celibacy may have adaptive properties—parents alleviate competition for parental resources between siblings by electing one of them to enter a monastery^3,4^. This carries a direct fitness cost for the parent because of the non-reproducing offspring, but this is potentially more than outweighed by an indirect fitness benefit obtained by increasing the competitiveness of their other offspring. This, in turn, is expected to enhance the reproductive fitness of the monk’s brothers or the nun’s sisters, thereby conferring inclusive fitness benefits to the celibate’s parents. Extending this framework, an inclusive fitness model of celibacy recently published by Micheletti and Mace^5^ suggests that the sex for which marriage costs are higher will have more celibates. Here, we have compiled a cross-cultural database on celibacy to empirically test a set of hypotheses proposing that resource competition within the family—generated in part by marriage patterns—is linked to both the occurrence of celibacy and the sex of celibates across societies, directly testing the predictions outlined in Micheletti and Mace^5^.

Humans invest heavily in their offspring, and this has been associated with positive offspring outcomes^6,7^. However, parents face a trade-off: as family size grows, the resources available per offspring decline, intensifying sibling competition and reducing parental care per child^8,9^. When resources are finite (or rival, i.e., their value declines with division; most commonly material wealth but also parental care^10^), this can result in unequal resource distributions among siblings, influencing their reproductive success and parental inclusive fitness^11,12^. Similar patterns occur in non-human animals where parental resources cannot be fully shared or decline with larger broods^13,14^. Intense competition among offspring drives the evolution of adaptive strategies^15,16^, such as dispersal from the natal nest^17,18^, brood reduction through hatching asynchrony^19,20^, parental discrimination^21,22^, and even infanticide^23^.

In many human societies, the pressures created by rival resources have produced strategies analogous to those seen in non-human animals^11,24–26^. One such strategy is the removal of individuals from the reproductive pool through celibacy, which can enhance the reproductive prospects of relatives^3,4,27^. It has been shown that men with a monk brother or son are, on average, wealthier and have more children than those whose male kin remain non-celibate^4^. A well-documented example comes from the ecologically constrained Himalayan hinterlands, where families avoid fragmenting scarce arable land by marrying multiple brothers to a single wife (polyandry)^28^. To reduce competition between same-sex siblings within polyandrous marriage, parents donate surplus sons to monasteries. For instance, in the village of Ladakh in India, as many as two-thirds of single men were monks^29^. Conversely, in the Nepalese village of Sama, a lack of marriage opportunities among daughters resulted in 19% of the female population becoming nuns^30^.

Similar dynamics occur in patrilineal societies where wealth is passed down the male line, increasing competition for reproductive opportunities between brothers^11,31,32^. In land-limited production systems of medieval and early modern Europe, for example, patriliny and restricted inheritance resulted in an increasing number of downwardly mobile sons who entered military or clergy ranks^33,34^. In societies where marriage customarily involves wealth transfers from the bride’s side of the family to the groom’s and eventually to the couple or the bride (dowry), competition between women is intense^35–39^. This was the case, for example, among middle- to upper-status families in historical China, where parents commonly sent their daughters to Buddhist nunneries^40^.

**Fig. 1:**
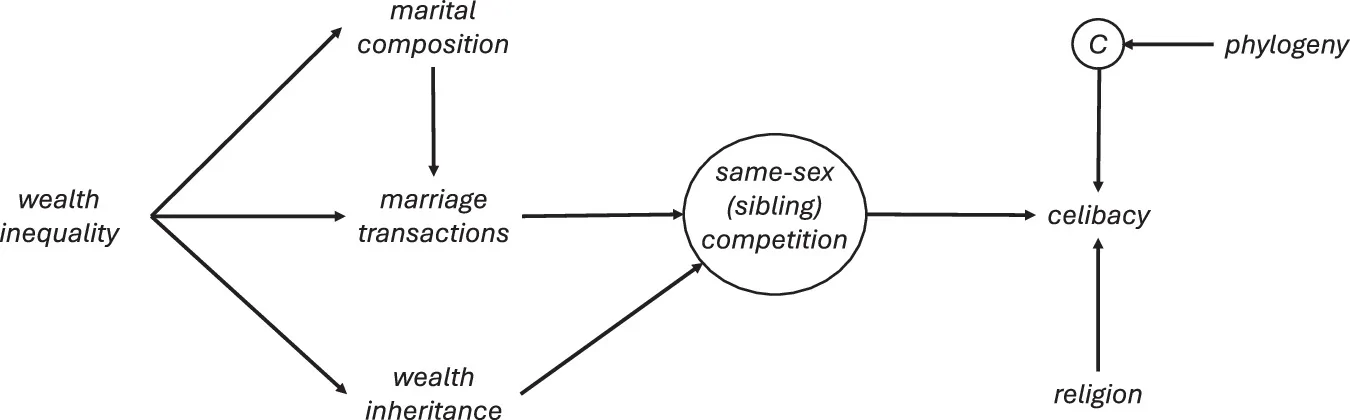
Directed acyclic graph depicting our assumptions about the processes that generate celibacy at the societal level. Nodes represent the main variables in the study; arrows indicate causal relationships among them. Encircled nodes represent latent variables, with *C* referring to unobserved cultural factors. Celibacy refers to the presence of any form of celibacy; marital composition refers either to polygyny, monogamy or polyandry; marriage transactions refer to any form of transaction of a substantial number of resources at marriage; wealth inheritance refers to the presence of any form of wealth inheritance; wealth inequality represents social stratification scale. The causal structure also applies to sex-specific celibacy but the variables have different meanings: celibacy refers to either male or female celibacy; marital composition refers to polygyny in male celibacy model or monogamy/polyandry in model for female celibacy; marriage transactions include either transactions from the groom’s side or both sides in male celibacy model or transactions from the bride’s side or both sides in model for female celibacy; wealth inheritance refers to patrilineal or bilateral inheritance; wealth inequality remains unchanged (see Methods for a verbal description of the model).

These ethno-historical examples illustrate that celibacy is often found under restricting local socio-ecological conditions. In this work, we investigate the extent to which such conditions, related particularly to marriage patterns, influence variation in celibacy cross-culturally. This work provides a systematic test of these observations. We do this by examining the relationship between marriage patterns and celibacy, using an eHRAF World Cultures^41^ online database containing expertly indexed ethnographic data for 352 societies. Our predictions follow from the causal assumptions outlined in the directed acyclic graph (DAG) in Fig. 1.

We assume same-sex sibling competition as the key mechanism underlying the emergence of celibacy. It is latent in our study, however, as we have not measured it directly. Building on the rationale of previous studies^11,12^, we assume that the unequal distribution of resources (wealth inequality) biases the intensity of competition between same-sex siblings for parental resources. We use the type of wealth transfers at marriage (marriage transactions), the number of concurrent spouses (marital composition), and the pattern of inheritance passed down kinship descent lines (wealth inheritance) as causes of the competition between same-sex siblings. We anticipate that marriage transactions involving substantial transfers of wealth predict celibacy. This creates economic thresholds that some individuals cannot meet, effectively excluding them from the mating pool^40^. We further expect that male or female celibacy is more likely to be found where marriage costs are higher for that sex^5^: transfers from the groom’s side of the family (bride-wealth) generate competition for parental resources between male siblings that should result in more male celibacy; transfers from the bride’s side (dowry) generate competition between female siblings, leading to more female celibacy. With respect to marital composition, we expect that celibacy is more likely to occur in societies where the number of marriage partners between the sexes is equal (i.e., under monogamy; see ref. ^10^ for a discussion on wealth inequality and monogamy). A corollary of this reasoning is that societies where males marry more than one female (polygyny) are more likely to be associated with male celibacy, and societies where females marry more than one male (polyandry) are more likely to be associated with female celibacy. We expect that modes of wealth inheritance, which increase competition over rival resources—especially family land—will be associated with elevated rates of celibacy^27^. In patrilineal inheritance systems, increased competition between male siblings is likely to result in more male celibacy. The converse might be expected for female celibacy, where wealth is passed down the female line (matriliny). However, given that matriliny is much less frequent than patriliny and inheritance in matrilineal societies is commonly passed down diagonally (to nephews), we do not expect matrilineal inheritance to play a role in driving female celibacy and thus leave this prediction out from testing.

**Fig. 2:**
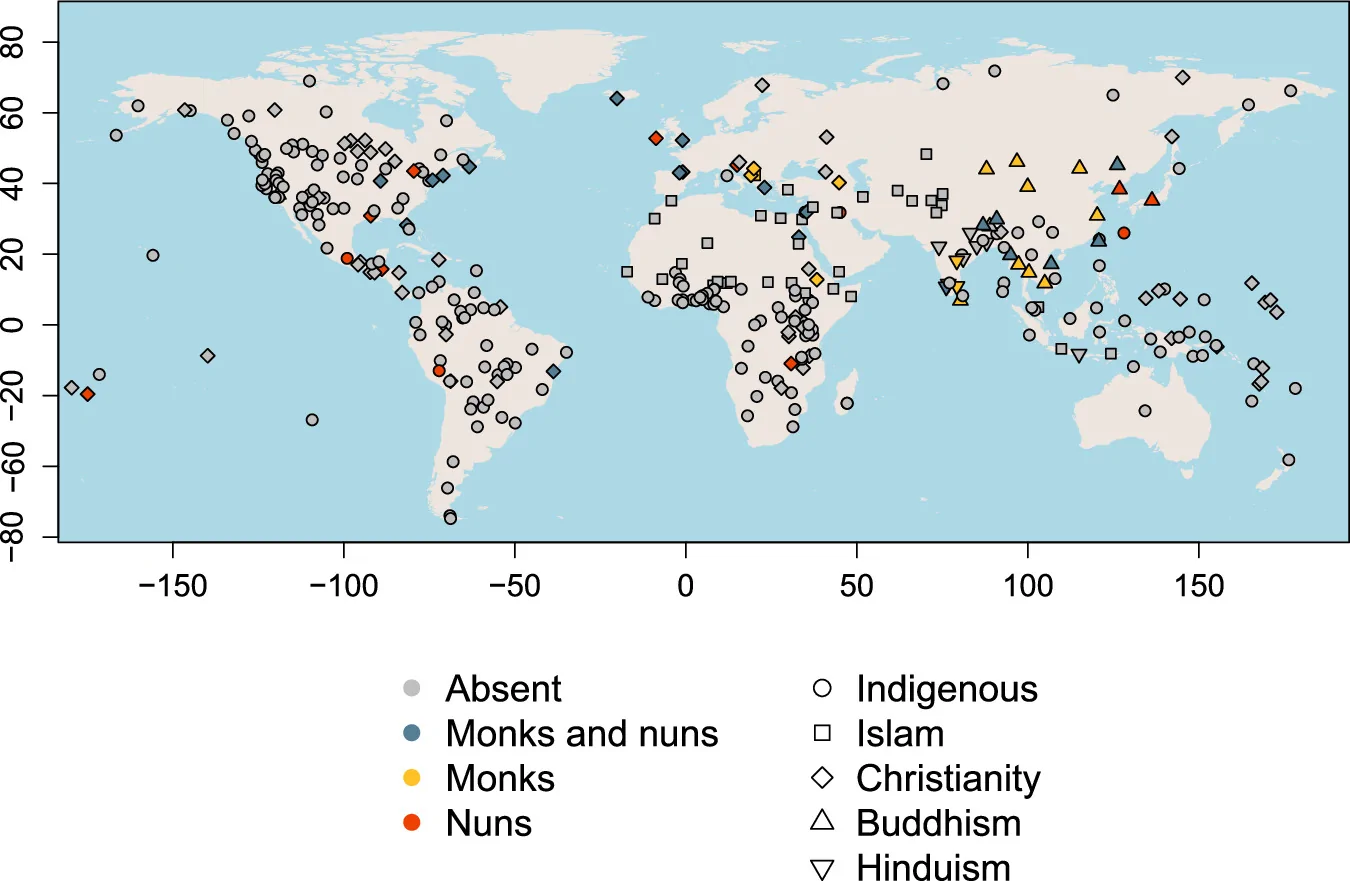
Geographic distribution of religious celibacy in the study sample (*N* = 352). The points are superimposed on major world religious traditions (shaped points), distinguishing societies with both monks and nuns (cyan), only monks (yellow) and only nuns (red) or with no celibacy (gray). Celibacy data were drawn from the primary ethnographic literature accessed at eHRAF World Cultures database (see Supplementary Methods in the SI for details on retrieving and coding the data). The map was created in RStudio software.

Finally, we expect that these dynamics are influenced by religion, as certain aspects of some religions may be more or less conducive to celibacy^42^, and cultural relatedness due to the shared ancestry between societies. Because celibacy has been historically more widespread in Eurasia than in any other part of the globe (Fig. 2), we also performed analyses of Eurasian societies alone. To test our hypotheses, we used Bayesian phylogenetic multilevel models where each of the tested relationships was estimated in a separate model adjusted for a set of potential confounds, as implied by our DAG (see Methods for further details). Inference was based on model predictions and on simulations of new outcomes under different conditions.

Here, we find that celibacy is more common in monogamous societies and where wealth, especially land, is heritable. Male and female celibacy are more common where marriage payments are higher for the celibate sex. Compared to other major religions, Buddhism and Christianity are more likely to have celibacy, which may provide an example of a parallel cultural evolution of a particular kind of religious institution in the face of similar socio-ecological drivers.

## Results

### Presence or absence of celibacy

**Fig. 3:**
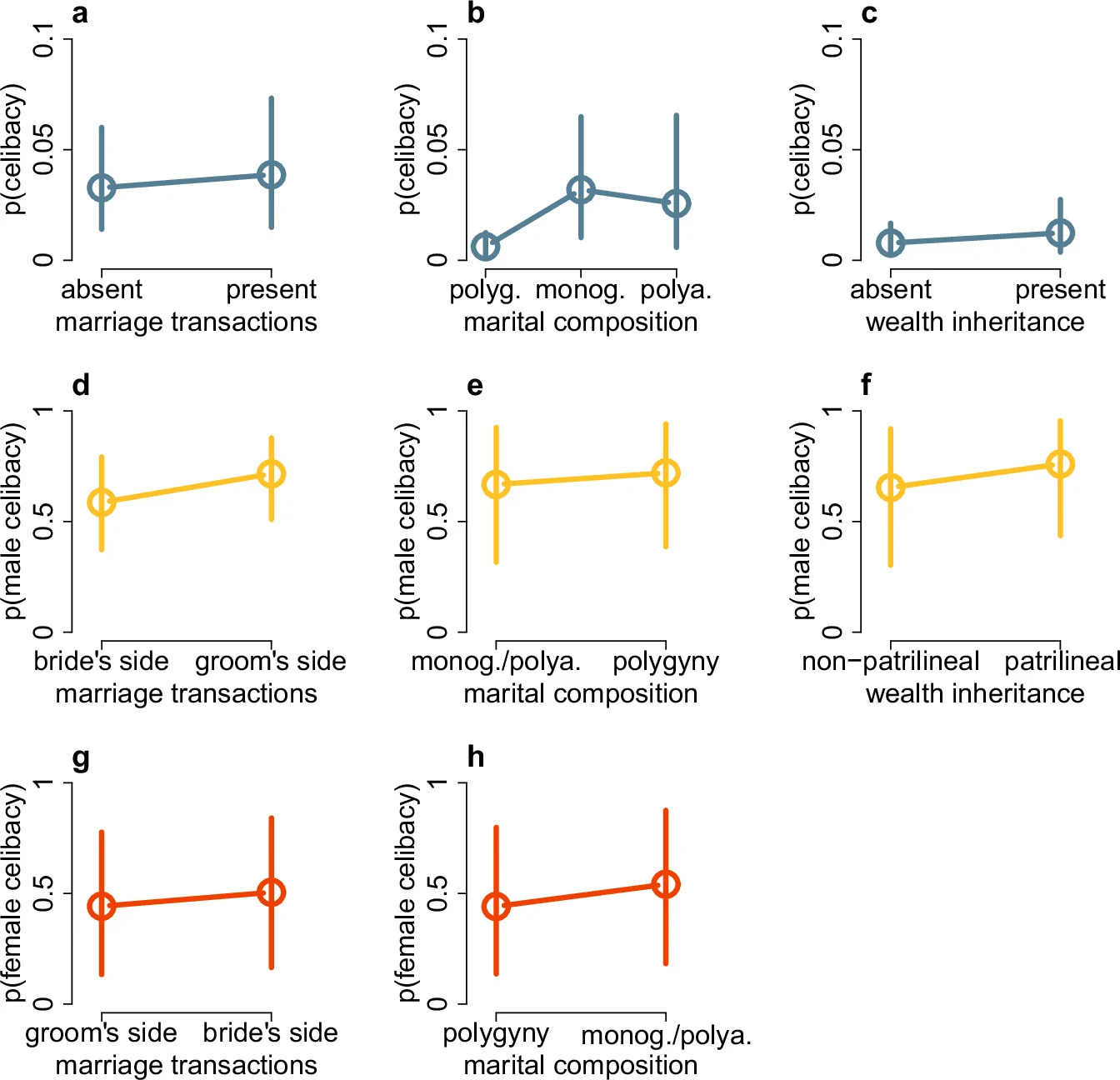
Model predictions for total-effect estimates for all-societies (*N* = 333) and sex-specific celibacy (*N* = 44) samples. **a–c** Celibacy (cyan); **d–f** Male celibacy (yellow); **g**, **h** Female celibacy (red). Open points indicate posterior means and vertical lines are 90% credibility intervals obtained by averaging over the estimates’ posterior distributions, each containing 2000 parameter values. The plot (**b**) shows estimates from the model where marital composition was modeled as a categorical variable. All plots are on the probability scale (vertical axis). The very small effects in plots (**a–c**) are due to a very low probability of celibacy in all-societies sample, captured by intercept parameters. Therefore, these plots are on a 0–0.1 scale. polyg. polygyny, monog. monogamy, polya. polyandry.

Our Bayesian phylogenetic multilevel models showed that, in all-societies sample, marriage transactions—either from the groom’s side, bride’s side or both sides—were associated with an increased likelihood of lifelong celibacy but the effect was weak (mean = 0.15, 90% CI [−0.42, 0.72]), with the estimate’s posterior being only 67% in the predicted direction (Supplementary Table 1 and Fig. 3a). With respect to marital composition—ordered from polygyny, through monogamy, to polyandry—we first modeled it as an ordinal predictor, where the effects across levels were forced to increase incrementally and sum to one (see ref. ^43^), which showed that the maximum effect of marital composition was reliably positive (1.23 [0.59, 1.84]; Supplementary Table 1 and Supplementary Fig. 1a), with the estimate’s posterior being >99% in the predicted direction. The log odds of celibacy increased by 0.77 [0.50, 0.95] when transitioning from polygyny to monogamy, and by 0.23 [0.05, 0.50] from monogamy to polyandry (Supplementary Fig. 1b). We then modeled marital composition as a categorical predictor—implying no relationship between marriage systems—which showed that polygyny reliably decreased the log odds of celibacy (−1.15 [−1.79, −0.52]), while monogamy (0.51 [−0.15, 1.17]) and, to a lesser extent, polyandry (0.18 [−0.62, 0.94]) increased it (Supplementary Fig. 2). Celibacy was also more likely in societies where intergenerational transfers of immovable property, i.e., land, occurred (0.45 [−0.16, 1.08]). The posterior distribution of this effect was 89% in the predicted direction (Fig. 3c and Supplementary Table 1). The analysis of the Eurasian subsample, including marital composition modeled as both ordinal and categorical, produced similar results, though there was no effect of marriage transactions (0.03 [−0.63, 0.66], 54% PP; Supplementary Table 2 and Supplementary Figs. 3a–c–5). Overall, in line with our prediction, marital composition—particularly monogamy—exerted the greatest influence on the likelihood of celibacy, in both all-societies and in Eurasian samples.

### Male celibacy

As predicted, societies where transactions at marriage from the groom’s side occurred were more likely to have male than female celibacy (0.62 [−0.07, 1.34]), with the estimate’s posterior being 93% in the predicted direction (Supplementary Table 1 and Fig. 3d). Polygynous societies were more likely to have male celibacy compared to monogamous or polyandrous societies, but the effect was relatively modest (0.29 [−0.39, 0.97]) and less certain (76% PP; Supplementary Table 1 and Fig.3e). When marital composition was modeled as a categorical predictor, polygyny (0.23 [−0.46, 0.92]) and polyandry (0.20 [−0.60, 0.98]) increased the log odds of male celibacy, while monogamy decreased it (−0.30 [−1.03, 0.40]; Supplementary Fig. 6). However, the effects were relatively modest and uncertain. Male celibacy was also more likely in societies with patrilineal inheritance of wealth (0.61 [−0.17, 1.35]), with 90% of the posterior in the predicted direction (Supplementary Table 1 and Fig. 3f). Analysis of the Eurasian subsample yielded similar results, though the effect of wealth inheritance decreased (0.21 [−0.58, 1.04], 67% PP; Supplementary Table 2 and Supplementary Figs. 3d–f and 7). Nevertheless, these results show that male celibacy is more likely to occur in societies where wealth is transferred at marriage from the groom’s side and/or inherited patrilineally across generations.

### Female celibacy

Societies where transactions at marriage were from the bride’s side (0.30 [−0.39, 1.01]) and where either monogamy or polyandry occurred (0.49 [−0.22, 1.19]) were more likely to have female than male celibacy, with the estimates’ posteriors 76% and 88% in the predicted direction, respectively (Supplementary Tables 1 and 2 and Fig. 3g, h). When marital composition was modeled as categorical, monogamy (0.37 [−0.35, 1.08]), and only weakly polyandry (0.08 [−0.70, 0.88]), increased the log odds of female celibacy, while polygyny decreased it (−0.45 [−1.17, 0.27]; Supplementary Fig. 8). The Eurasian subsample yielded similar results, but with a notably stronger effect of marriage transactions (0.53 [−0.18, 1.25]; 88% PP; Supplementary Table 2 and Supplementary Figs. 3g, h and 9). Overall, female celibacy is more likely to occur in monogamous societies with transactions of wealth from the bride’s side.

### Evaluating the variation in celibacy accounted for by phylogeny and religion

Incorporating multiple variation components simultaneously allowed us to evaluate their relative contributions to the total variation in celibacy. We found that, on average, and considering both all-societies and Eurasian samples, the probability of a society having at least some celibate members was about two times higher across religions, i.e., intercepts, than the variation due to phylogenetic relatedness based on language similarity (Supplementary Figs. 10 and 11a–c). Similarly, religion accounted for about 30% more variation in the probability of male celibacy being present than phylogeny (Supplementary Figs. 10 and 11d–f). In contrast, in most of the models for female celibacy, phylogenetic relatedness accounted for about 30–50% more variation than the variation across religions, particularly in the Eurasian subsample (Supplementary Figs. 10 and 11g, h). There was also substantial variation across religions in the effects of the tested predictors, i.e., slopes, which was particularly high in models for male celibacy (Supplementary Figs. 10 and 11d–f).

Stratifying the slopes also allowed us to compare effects of the tested predictors between religions. We found that Buddhism and, similarly but to a lesser degree, Christianity exhibited the strongest support for our predictions, in terms of both magnitude and directionality of effects. Specifically, marriage transactions increased the log odds of celibacy in Christian (0.63 [−0.20, 1.82], 84% PP) and, only weakly, Buddhist (0.10 [−1.41, 1.78], 55% PP) societies, while in all other religions the estimates were negative (Supplementary Table 3). The same pattern was found in the Eurasian subsample (Supplementary Table 4). Transactions from the groom’s side increased the log odds of male celibacy most notably in Buddhist societies in both all-societies (2.10 [−0.14, 5.99], 91% PP) and Eurasian (2.86 [−0.06, 7.30], 93% PP) samples, followed by Christian societies in the latter sample (1.72 [−0.68, 6.08], 82% PP; Supplementary Tables 3 and 4). Only in Indigenous religions the effect was negative (both samples). Transactions from the bride’s side increased the log odds of female celibacy, again, most notably in Buddhist (0.71 [−0.31, 2.62], 78% PP) societies, while in the Eurasian subsample it was most notable in both Buddhist (0.77 [−0.5, 2.63], 80% PP) and Christian (0.82 [−0.41, 2.97], 79% PP) societies, followed by only weakly supported Indigenous religions (60% PP). There was no association in Islamic and Hindu societies in either sample (Supplementary Tables 3 and 4), likely because these religions include very little celibacy of any kind.

Marital composition—ordered from polygyny, through monogamy, to polyandry—increased the likelihood of celibacy most notably in Buddhist (all societies: 0.29 [−0.63, 1.81], 64% PP; Eurasia: 0.60 [−0.44, 2.51], 73% PP) and Christian (all societies: 0.33 [−0.34, 1.37], 76% PP; Eurasia: 0.16 [−0.78, 1.32], 59% PP) societies, followed by societies with Indigenous religions in the all-societies sample (0.22 [−0.78, 1.57], 62% PP), while the estimates were negative in Islamic and Hindu societies in both all-societies and Eurasian samples (Supplementary Table 5, 6). However, in all religions, effects of marital composition on celibacy were relatively weak and uncertain. Polygyny increased the log odds of male celibacy particularly in Buddhist societies (1.71 [−0.13, 5.22], 89% PP), and to a lesser extent in Islamic (0.78 [−1.14, 3.89], 67% PP) and Hindu (0.51 [−1.87, 3.71], 59% PP) societies, while it was negative in societies with Indigenous and Christian religions (Supplementary Table 5). This is likely because there are numerous Indigenous as well as Christian societies—especially outside Eurasia—that, despite being polygynous, do not have male celibacy. However, in Christian societies in the Eurasian subsample, this effect turned positive but was uncertain (69% PP; Supplementary Table 6). Because eight out of nine Christian societies in our Eurasian subsample that have male celibacy are monogamous, this result should be interpreted with caution. In both all-societies and Eurasian samples, monogamy or polyandry increased the likelihood of female celibacy in all religions except Islam, showing no relationship, while it was most notable in Christian (0.38 [−0.63, 1.91], 69% PP), followed by Indigenous (0.36 [−0.77, 2.35], 62% PP) and Buddhist (0.28 [−0.66, 1.63], 64% PP) religions. The Eurasian subsample replicated the results (Supplementary Tables 5 and 6). Nonetheless, effects of monogamy/polyandry on female celibacy in both samples were relatively weak and uncertain.

Wealth inheritance showed no effects on celibacy in all religions in the all-societies sample, while it increased the log odds of celibacy only in Christian societies in the Eurasian subsample (0.57 [−0.46, 2.61], 71% PP; Supplementary Tables 7 and 8). In contrast, patrilineal inheritance increased the log odds of male celibacy most prominently in Christian societies in the all-societies sample (2.01 [−0.15, 5.27], 91% PP) and Buddhist societies in the Eurasian subsample (1.05 [−0.54, 3.78], 78% PP), followed by Hindu and Islamic societies, while it was negative in Indigenous religions in both samples (Supplementary Tables 7 and 8).

Taken together, although the reported estimates sometimes formed a gradient across religions, in most models, Buddhism along with Christianity were mostly responsible for the observed patterns. Specifically, in Buddhist societies, male celibacy was associated more strongly with marriage transactions and marital composition, whereas in Christian societies, it was more strongly influenced by inheritance systems. In both religions, and to a lesser extent in Indigenous ones, female celibacy was more strongly associated with marriage transactions, though it was generally less prominent.

### Simulating the distributions of celibacy in Buddhist and Christian societies

To better illustrate the implications of our models, we simulated celibacy distributions across different predictor values (0/1), averaging over the posterior distributions of the estimated parameters. We then assessed differences in the resulting frequencies of celibacy between simulated conditions using chi-squared test. Based on the findings from our varying-intercept/varying-slope models, here we present simulations for Buddhism and Christianity only; simulations based on models not specific to any religion are provided in the Supplementary Figs. 12 and 13.

**Fig. 4:**
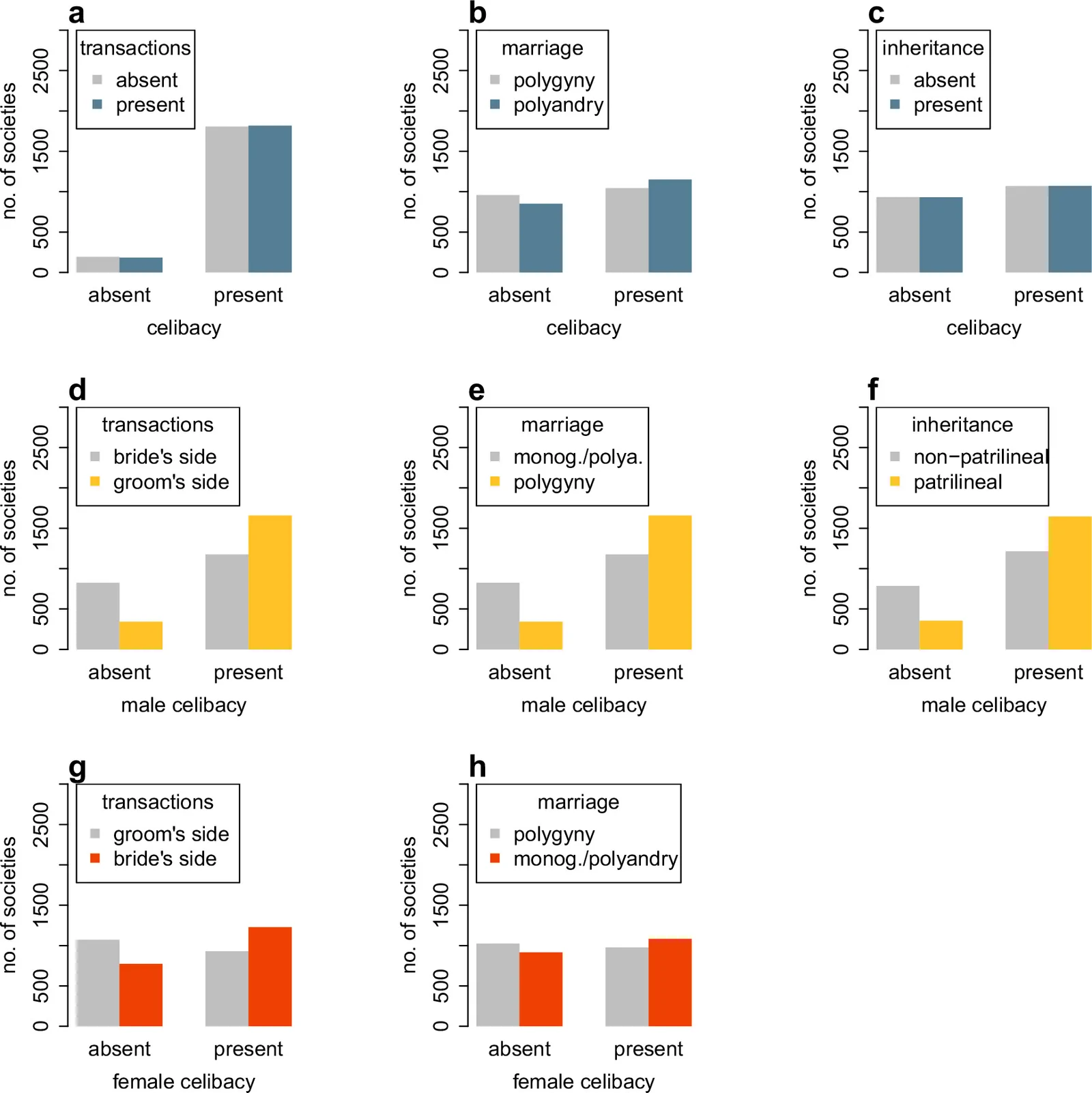
Distributions of celibacy rates in simulated Buddhist societies based on models for all-societies (*N* = 333) and sex-specific celibacy (*N* = 44) samples. **a–c** Celibacy; **d–f** Male celibacy; **g**, **h** Female celibacy. Outcomes were simulated under different values (gray for absence; cyan for presence in models for celibacy; yellow for presence in models for male celibacy; red for presence in models for female celibacy) of each predictor, averaging over the estimates’ posterior distributions, each containing 2000 parameter values. Celibacy distributions in plot (**b**) are simulated only under polygyny and polyandry, while omitting monogamy (see Methods for details).

In the simulated samples of Buddhist societies, differences in the rates of lifelong celibacy between societies with and without substantial transfers of resources at marriage and heritable wealth, i.e., land, were not statistically significant (Fig. 4a–c). Only the presence of polyandrous marital composition showed a statistically significant difference in the rate of lifelong celibacy (${\chi }_{(1)}^{2}$ = 5.221, *p* = 0.022; Fig. 4b), a pattern also observed in the Eurasian subsample (${\chi }_{(1)}^{2}$ = 4.933, *p* = 0.026; Supplementary Fig. 14b). By contrast, the presence of transactions from the groom’s side (${\chi }_{(1)}^{2}$ = 82.347, *p* < 0.001), polygyny (${\chi }_{(1)}^{2}$ = 64.772, *p* < 0.001) and patrilineal inheritance (${\chi }_{(1)}^{2}$ = 64.650, *p* < 0.001) showed a statistically significant difference in male celibacy compared to their absence (Fig. 4d–f). Notably, Buddhist societies with these practices but without male celibacy were exceedingly rare, with all these differences being statistically significant (transactions: ${\chi }_{(1)}^{2}$ = 199.900, *p* < 0.001; marital composition: ${\chi }_{(1)}^{2}$ = 172.490, *p* < 0.001; inheritance: ${\chi }_{(1)}^{2}$ = 162.190, *p* < 0.001; Fig. 4d–f). The pattern was also observed for the Eurasian subsample (transactions: ${\chi }_{(1)}^{2}$ = 347.100, *p* < 0.001; marital composition: ${\chi }_{(1)}^{2}$ = 189.510, *p* < 0.001; inheritance: ${\chi }_{(1)}^{2}$ = 104.440, *p* < 0.001; Supplementary Fig. 14d–f). The simulations indicated statistically significant difference in the rates of female celibacy in societies with transactions from the bride’s side (${\chi }_{(1)}^{2}$ = 41.485, *p* < 0.001) and those practicing monogamy or polyandry (${\chi }_{(1)}^{2}$ = 5.770, *p* = 0.017; Fig. 4g, h), which was also found for the Eurasian subsample (transactions: ${\chi }_{(1)}^{2}$ = 39.368, *p* < 0.001; marital composition: ${\chi }_{(1)}^{2}$ = 4.085, *p* = 0.043; Supplementary Fig. 14g, h).

**Fig. 5:**
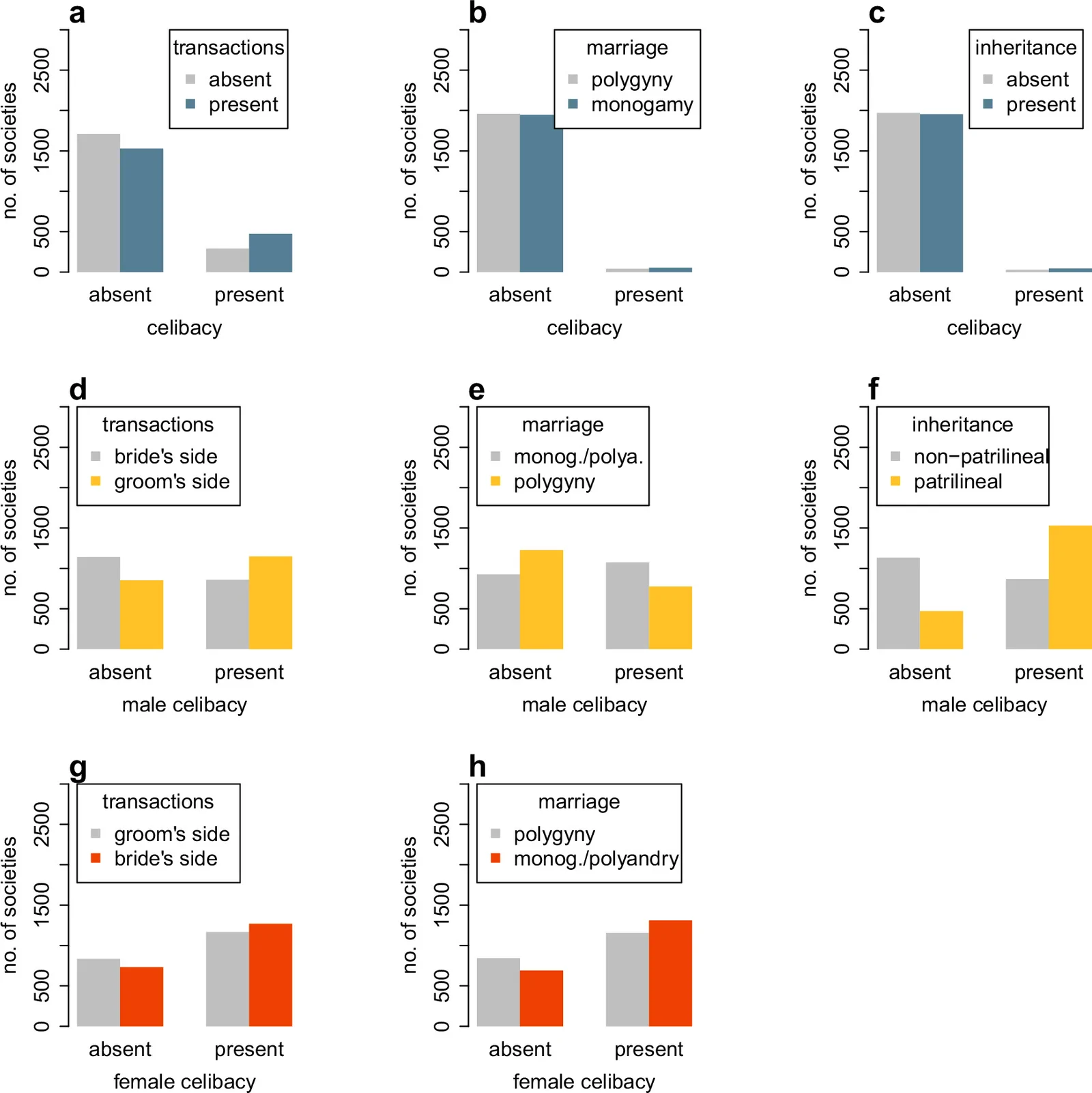
Distributions of celibacy rates in simulated Christian societies based on models for all-societies (*N* = 333) and sex-specific celibacy (*N* = 44) samples. **a–c** Celibacy; **d–f** Male celibacy; **g**, **h** Female celibacy. Outcomes were simulated under different values (gray for absence; cyan for presence in models for celibacy; yellow for presence in models for male celibacy; red for presence in models for female celibacy) of each predictor, averaging over the estimates’ posterior distributions, each containing 2000 parameter values. Celibacy distributions in plot **b** are simulated only under polygyny and monogamy, while omitting polyandry (see Methods for details).

In the simulated Christian societies, only the presence of transfers of relatively greater amount of resources at marriage generated statistically significant difference in celibacy (${\chi }_{(1)}^{2}$ = 43.470, *p* < 0.001; Fig. 5a–c), while in the Eurasian subsample of Christian societies, both the presence of marriage transactions (${\chi }_{(1)}^{2}$ = 24.775, *p* < 0.001) and heritable wealth (${\chi }_{(1)}^{2}$ = 26.648, *p* < 0.001) produced statistically significant differences in the rates of celibacy (Supplementary Fig. 15a–c). Marital composition did not produce statistically significant differences in celibacy in either the all-societies sample (${\chi }_{(1)}^{2}$ = 1.779, *p* = 0.182; Fig. 5b) or Eurasian subsample (${\chi }_{(1)}^{2}$ = 1.968, *p* = 0.161; Supplementary Fig. 15b). It should be noted that, unlike for Buddhist societies, celibacy rates for Christian societies were simulated for polygyny and monogamy. Like Buddhist societies, transactions from the groom’s side (${\chi }_{(1)}^{2}$ = 41.307, *p* < 0.001) and patrilineal inheritance (${\chi }_{(1)}^{2}$ = 183.380, *p* < 0.001) generated statistically significant difference in male celibacy compared to the absence of these practices (Fig. 5d–f), which was also the case for the Eurasian subsample (transactions: ${\chi }_{(1)}^{2}$ = 47.773, *p* < 0.001; inheritance: ${\chi }_{(1)}^{2}$ = 22.438, *p* < 0.001; Supplementary Fig. 15d–f). While polygyny was associated with a statistically significant decrease in male celibacy in the all-societies sample of Christian societies (${\chi }_{(1)}^{2}$ = 48.649, *p* < 0.001; Fig. 5e), there was a statistically significant increase in male celibacy under polygyny in the Eurasian subsample (${\chi }_{(1)}^{2}$ = 7.4279, *p* = 0.010; Supplementary Fig. 15e). As mentioned above, the result of the model that this simulation is based on should be interpreted with caution.

Finally, there were statistically significant differences in female celibacy under the presence of transactions from the bride’s side (${\chi }_{(1)}^{2}$ = 4.271, *p* = 0.04) as well as monogamy or polyandry (${\chi }_{(1)}^{2}$ = 9.497, *p* = 0.002; Fig. 5g, h). These differences were even more pronounced in the Eurasian subsample of Christian societies (transactions: ${\chi }_{(1)}^{2}$ = 42.632, *p* < 0.001; marriage: ${\chi }_{(1)}^{2}$ = 18.671, *p* < 0.001; Supplementary Fig. 15g, h).

## Discussion

In this study, we show that marriage patterns, assumed here to be proxies for same-sex sibling competition for heritable wealth, predict cross-cultural distributions of lifelong religious celibacy—including which sex is most often associated with celibacy in societies. Celibacy is found predominantly in non-polygynous societies with heritable wealth, and its occurrence among men or women aligns with which sex faces higher marriage payments. Dowry was associated with nuns and bride-wealth with monks. These results provide direct empirical support for the inclusive fitness model proposed by Micheletti and Mace^5^.

Numerous ethnographic examples derived from our own research corroborate this pattern. In early 20th-century Mongolia, widespread bride-wealth and polygyny coincided with up to 40% of men becoming Buddhist monks^44^. In Thailand, the decline of monasticism in the 1960s followed the introduction of mandatory public education and expanding economic opportunities, which reduced sons’ dependence on parental resources^45^. In South Korea’s Chungcheong province, where daughters received large dowries and monogamy prevailed, nunneries outnumbered monasteries by a factor of three^46^. Similarly, in late medieval and early modern Europe, elite families faced escalating dowry demands due to hypergyny, i.e., marrying-up in status, and competition for noble titles, which led to high rates of female cloistering among the upper classes^5,47,48^.

Our analysis also uncovered similar factors associated with religious celibacy in both Buddhist and Christian societies, suggesting parallel cultural evolution of celibate institutions in response to similar socio-ecological pressures. Most of these societies are in mainland Eurasia and belong to a small number of language families—primarily Sino-Tibetan and Indo-European, but also Mongolic-Khitan, Dravidian, and Austroasiatic. Sino-Tibetan and Indo-European societies share important economic and demographic similarities: their subsistence was traditionally based on intensive agriculture, they had large and sedentary populations with high wealth inequality, and private land ownership was the main form of heritable wealth^33,49^. In such contexts, where resources are predictable but depletable, such as land, parents may be incentivized to allocate wealth to fewer offspring by directing others into celibate religious roles^3^.

While this view emphasizes parental agency—particularly parent-offspring conflict—influencing patterns of celibacy, recent theoretical model suggests that it is predominantly firstborn offspring that benefits in inclusive fitness terms from their siblings becoming celibate or foregoing reproduction in other ways^50^. These frameworks are not mutually exclusive: under certain conditions, primogeniture, i.e., inheritance passed on to the firstborn child, is the most viable option for both parents and firstborns. Thus, religious celibacy likely emerges from multiple interacting mechanisms, all compatible with an inclusive fitness perspective.

Although these patterns explain the prevalence of celibacy in many Eurasian societies, its absence in other regions—despite similar ecological or economic conditions—highlights important exceptions. For instance, polygyny and bride-wealth are widespread in many non-celibate societies in the eHRAF sample. The general absence of (male) celibacy in these societies may reflect a lower need to recruit individuals into non-reproductive roles. In much of Africa, for example, relatively low productivity afforded by extensive agriculture rarely led to the shortage of land, while livestock could be increased by husbandry^33,49^. Because heritable wealth in the form of land or animals was not so drastically depleted through division, sibling competition remained less intense^51^, potentially explaining the absence of institutional celibacy in Africa, even despite a long-standing presence of Christianity. These labor-limited systems contrast with the land-limited systems of Eurasia, where celibacy is relatively widespread. It should be noted, however, that these interpretations apply primarily to earlier periods (19th–20th centuries) and may not accurately reflect patterns of celibacy in modern-day Africa.

Another notable exception is the Inca empire. The *aclla* women of Cuzco (13th–17th centuries CE) were sequestered and required to remain celibate, with violations punishable by death^52^. However, there is no evidence for a formal institution of male celibacy, despite extreme reproductive inequality among men caused by the presence of immense harems held by elite males. This may be explained by relatively low male-male competition for heritable wealth: despite the presence of bride-wealth, it consisted of common goods, such as firewood or dried meat, not livestock, suggesting limited competitive pressures typical of other bride-wealth societies^52^.

Similarly, in Hindu societies, male celibacy aligns with our predictions, but female celibacy is notably absent. The socio-ecological conditions for female celibacy were met—particularly dowry inflation and high-status marriage competition^39,40^. Dowries placed severe financial burdens on families with multiple daughters, particularly in higher castes where inter-caste marriage was prohibited. In such contexts, convents might be expected. One possible explanation for their absence is that dowry inflation in India is relatively recent and traditionally limited to upper castes, whereas lower castes practiced bride-wealth^5,38^. An alternative explanation may be female infanticide, which was historically common in Bengal, Uttar Pradesh, and Punjab—regions with no tradition of female celibacy^53^. However, further research is needed to investigate the possible negative association between infanticide and religious celibacy.

Taken together, celibacy should be understood as one of several adaptive strategies that mitigate same-sex sibling competition for heritable wealth. These strategies include eliminating the need to fragment the land through polyandry^28^, reducing the number of spouses through monogamy^10^, limiting heirs via primogeniture^54^, or long-range dispersal, such as sending sons into military service—a common fate for later-born sons^4,5,31,50^. Such strategies may act as substitutes or occur alongside each other, particularly in contexts where both sexes compete for resources. This could explain why most societies where celibacy is present include celibates of both sexes, which, in turn, could account for somewhat diluted estimates reported here.

Religious affiliation remains a stronger source of variation than phylogeny in our models, suggesting that celibacy is to a greater extent influenced by historical contingencies unique to each religious tradition. In contrast, female celibacy shows slightly stronger influence from phylogeny than from religion (Fig. 4g, h and Supplementary Fig. 2g, h), indicating that it clusters more tightly among closely related societies. Female celibacy is also less common than male celibacy, although both often co-occur within the same societies, which make up most celibate societies in our sample. Notably, this sex-specific patterning can be observed in other cultural practices—such as genital mutilation/cutting and witchcraft accusations^55,56^. The underlying mechanisms driving these patterns remain unclear; in both cases, males tend to be more frequently targeted, while analogous female practices typically occur in association with those of males, warranting further investigation.

This study is subject to several limitations inherent to the use of ethnographic data. One persistent issue lies in determining whether the absence of a reported practice reflects its true absence or merely a lack of documentation, applying especially to our all-societies sample.

The key terms used to detect celibacy in the eHRAF database were mostly in English. The use of original terms (e.g., the Central Thai *bhikku* for monks and *mae chi* for nuns; the Cambodian *daun chi* for nuns; the Burman *thila-shin* or *bhikkuni* for nuns; or the Qing-period Chinese *ho-shang* or *seng-jen* for monks and *ni-ku* for nuns) may have resulted in a different search outcome. For example, ethnographers may have used the term *bhikkuni* instead of nun to refer to female celibates, the inclusion of which could potentially increase the count of female celibacy in the sample. However, as ethnographers were mainly writing in English, it is likely that they exclusively used English terms.

As most ethnographers of the time were male, there is a possibility that female celibacy and marriage practices concerning females were underreported. Females in these societies might have felt discouraged to divulge information to the male ethnographers or to include them in their spaces. However, male ethnographers would likely have still reported the presence of convents, especially given the novelty of female celibacy in some of these societies. More broadly, information as to whether and/or what kind of celibacy was present could have been excluded or ignored out of lack of interest or for other reasons by ethnographers. However, religious celibacy differs from other cultural practices in its degree of public visibility, making systematic non-observation by ethnographers less likely.

The extent to which ethnographies in eHRAF reflect the reality of the variation of male and female celibacy in these societies could be questioned. However, in several instances, national censuses collected by governmental institutions within these societies seemed to correspond to proportions of male and female celibacy emerging from the search. For example, according to the Ministry of Education of Thailand in 1976, there were 213,175 monks and 10,529 nuns (i.e., monks constituted 95.3% of the monastic population^57^). A count of the number of mentions of monks and nuns in Central Thai in eHRAF produced a similar statistic: monks constituted 97.4% of the celibate sample.

Lastly, another possible limitation concerns our coding decisions. Because both male and female celibacy most often occurred alongside each other, we grouped these societies with those where either only male or female celibacy occurred when coding sex-specific celibacy. This likely introduced uncertainty and may explain some relatively weak estimates, despite intuitive theoretical predictions. Dropping these overlapping cases would have drastically reduced our sample size, and alternative modeling approaches (e.g., multinomial models with societies having both monks and nuns as a reference category) would not have adequately tested our hypotheses. Future studies could therefore benefit from including original foreign-language terms, exploring the extent to which bias might configure into their sources as well as the impact coding decisions might exert on the quality of parameter estimates.

Lifelong religious celibacy, where individuals lose prospects of reproduction, has puzzled anthropologists who often viewed it through the lens of maladaptation or evolutionary mismatch. However, such explanations offer little predictive power regarding when or where this trait arises. In contrast, recent theoretical and empirical work has shown that celibacy can yield inclusive fitness benefits to a celibate’s kin and is likely to evolve in contexts of resource competition^3–5^.

Our study directly tested these predictions using a cross-cultural sample and Bayesian multilevel models formally adjusted for a set of potential confounding variables. We show that competition for heritable wealth is linked to lifelong religious celibacy, and that its prevalence among men or women reflects which sex faces higher marriage payments. Thus, celibacy may co-evolve with marriage practices and serve as one of several adaptive strategies to maximize inclusive fitness in environments marked by intense resource competition. The presence of remarkably similar patterns across Buddhist and Christian societies suggests a parallel cultural evolution of comparable religious institutions in response to shared socio-ecological pressures.

## Methods

### Assumptions

The assumptions underlying our analysis are summarized in the DAG in Fig. 1. Based on a set of simple logical rules, DAGs help derive the set(s) of variables that the model must be conditioned on to obtain unbiased estimates from the data^58,59^. Note that the assumptions encoded in our DAG are not intended to represent a mechanistic model of lifelong religious celibacy. Rather, we use DAGs as a heuristic tool to mitigate confounding bias, which is pervasive in observational data. Although DAGs improve the transparency of the analysis, the resulting estimates remain sensitive to the specific assumptions implied by a given DAG. We nonetheless believe that the DAG in Fig. 1 adequately captures the relationships among the variables in our data.

Central to our causal model is the same-sex sibling competition for parental resources in the form of heritable wealth, which is considered the key mechanism driving celibacy—our exposure (same-sex competition → celibacy). Competition for wealth is unobserved, as we have not measured it directly (hence it is shown in a circle). However, we used three proxies that we assume are direct or indirect causes of resource competition and, ultimately, celibacy. Firstly, we assumed that marriage transactions are a direct cause of resource competition—male-male competition under transactions from the groom’s side, female-female competition under transactions from the bride’s side—and thus an indirect cause of celibacy (marriage transactions → same-sex competition → celibacy)^5,31,37,60^. Secondly, marital composition, defined as a socially recognized number of concurrent spouses for a given sex, i.e., polygyny, monogamy or polyandry, is hypothesized to influence resource competition indirectly through marriage transactions (marital composition → marriage transactions → same-sex competition → celibacy)^29,31,37^. This is because transfer of resources at marriage is the main mechanism driving competition for heritable wealth. Thirdly, patterns of wealth inheritance are assumed to be another cause of resource competition (wealth inheritance → same-sex competition)^11,31,37,60,61^. All three proxies are in turn directly influenced by wealth inequality, which is exogenous in our system (marital composition ← wealth inequality → marriage transactions; wealth inequality → wealth inheritance). We further assumed that the relationships we aimed to test vary across major world religions, which we considered an independent cause of celibacy (religion → celibacy). Lastly, there are likely unobserved cultural factors *C* shared among related societies, potentially confounding any relationship of interest, and for which we used language phylogeny as proxy (phylogeny → C → celibacy)^43,62^. Note that assumptions encoded in the DAG in Fig. 1 generalize to all forms of celibacy analyzed in this study.

### Data

Data for the present study were retrieved from eHRAF World Cultures and D-PLACE online databases^41,63^. An automated search of all societies included in eHRAF was conducted, of which 51 societies showed evidence for the presence of some form of lifelong religious celibacy (for details on the search and the coding process, see Supplementary Methods section in the SI). A form of sex-specific celibacy in society, i.e., male celibacy, female celibacy, or both male and female celibacy, was then determined. In addition, the main form of marital composition as well as marriage transaction were coded for all 51 celibate societies. In the remaining eHRAF societies, celibacy was inferred to be absent. However, it is vital to recognize that this constitutes absence of evidence and in no way indicates evidence of absence. In total, the sample included 352—both celibate and non-celibate—societies. Using society IDs, we then utilized D-PLACE to extract information on wealth inheritance (EA074), social stratification (EA066) as well as marital composition (EA009) and marriage transactions (EA006). Finally, for each society, information about a geographic region and a predominant religion was extracted from cultural summaries in eHRAF. The cultural summaries were also used to fill in missing values in some of our predictors. Societies with any number of missing values were excluded from analyses.

Celibacy—regardless of the sex of celibate and its form—was coded as 1 for its presence and 0 for its absence. As for the subsample of celibate societies, we created a binary indicator for the presence of male celibacy, e.g., monks, which included societies having male celibacy only, as well as societies having both male and female celibacy, as the latter societies constitute the majority (*N* = 21) of celibate societies. The presence of female celibacy, e.g., nuns, was coded in the same manner. These three binary indicators represented our outcome variables.

Marriage transactions from D-PLACE were coded as a binary indicator for the presence of any of the following transactions—bride-wealth, dowry, or gift exchange—to code the presence of transactions where relatively greater amounts of resources are being transferred at marriage, in contrast to societies where transactions at marriage involve fewer resources (token bride-wealth), take another form (bride-service) or are entirely absent. This was used for the analysis of celibacy. Marriage transactions from eHRAF were divided in two binary indicators—one for the presence of bride-wealth and where transactions from both groom’s and bride’s families occur, to clump together societies where competition for heritable wealth is primarily between males, the other for the presence of dowry and, again, where transactions from both sides occur, to code for societies where competition for parental resources is primarily between females. They were used for the analysis of male and female celibacy, respectively.

Marital composition from D-PLACE was coded as a 3-level ordinal scale—from polygyny, through monogamy, to polyandry—to reflect an increasing competition for parental resources. This was used for the analysis of celibacy. Marital composition from eHRAF was divided in two binary indicators—one for the presence of polygyny to reflect competition for heritable wealth between males, the other for the presence of monogamy or polyandry to reflect competition for heritable wealth between females. They were used for the analysis of male and female celibacy, respectively.

Wealth inheritance, which included immovable property, i.e., land, was coded, using D-PLACE, as a binary indicator for the presence of any form of land inheritance, in contrast to societies where intergenerational transmission of land was non-existent, and, as such, it was used for the analysis of celibacy. For the analysis of male celibacy, wealth inheritance was coded as a binary indicator for societies where inheritance by sons and both sons and daughters occurred.

We used class differentiation from D-PLACE, a 5-level ordinal variable, ranging from absence of classes, wealth distinctions, elite and dual stratification, to complex stratification, as a proxy for wealth inequality^64,65^.

Religion consisted of five categories—Indigenous, Islam, Christianity, Buddhism and Hinduism. Our coding of religion did not differentiate between ancient and recent spread of any of the major religions in a society, or between various branches within these religions.

Finally, we built a language tree to account for phylogenetic autocorrelation as well as for the influence of unobserved cultural factors *C*. We used glottocodes provided by D-PLACE to match each society with its language that was searched for at the Automated Similarity Judgment Program (ASJP) website^66^. For societies whose language was not included in ASJP (*N* = 12), we used their closest ASJP proxy language, as given by Glottolog^67^. Next, we downloaded a file with wordlists for each language and used software available at the ASJP website to calculate a pairwise distance matrix between languages. The tree was then constructed, with the distance matrix as an input, using the neighbor-joining algorithm in MEGA 11 software^68^. Societies that either speak the same language or a different dialect of the same language (*N* = 21) were added to the tree manually—as polytomies.

### Analysis

Analyses were conducted on four (sub)samples: (1) all-societies analysis of celibacy (*N* = 333); (2) analysis of celibacy in Eurasia (*N* = 91); (3) analysis of sex-specific, i.e., male and female, celibacy (*N* = 44); and (4) analysis of sex-specific, i.e., male and female, celibacy in Eurasia (*N* = 34). The decision to analyze also the Eurasian subsamples stems from the fact that, historically, celibacy has been much more widespread in Eurasia than in any other part of the world. Even despite the recent spread of some of the major world religions, e.g., Christianity, celibacy is rare outside Eurasia (apart from some immigrant communities in North America, see Fig. 2).

We modeled the probability of celibacy, and likewise that of male and female celibacy, using Bernoulli regression with a logit link. All models were adjusted for potentially confounding variables, as implied by our DAG (Fig. 1), and each estimate was obtained by fitting a separate model^69^. To account for the phylogenetic covariance, we used a correlation matrix derived from the language tree.

We then refitted the models where we stratified both intercepts and slopes by the varying effects of religion, which were assumed to be identically and independently distributed. By doing so, we aimed to test whether the observed patterns from previous models replicate across religions as well as to evaluate relative contributions of both components—religion and phylogeny—to the overall variation in celibacy. The same procedure was repeated for each (sub)sample. While our models assumed constant variance within each religious category, the category of Indigenous religions grouped together all faiths not classified under major world religions. As a result, treating it as equivalent to categories like Buddhism or Christianity may lead to overdispersion. However, celibacy is nearly universally absent among Indigenous religions in our sample.

We used the *rethinking* package (v2.42)^43^, which utilizes Hamiltonian Monte Carlo engine Stan (v2.35.0)^70^, to generate posterior distributions for model parameters. We ran four parallel chains, each with a 50% warm-up and one thousand iterations, resulting in 2000 posterior samples. Convergence was assessed by Gelman-Rubin diagnostic and the number of effective samples. Weakly regularizing priors—normal priors for intercepts and slopes, exponential priors for standard deviations—were assigned to all parameters to prevent the model from overfitting the data. To summarize the posteriors, we used the mean, 90% credibility intervals (CI), and the percentage of posterior probability on the expected side of zero (%PP). Rather than relying on dichotomous significance thresholds, the %PP offers a direct probabilistic interpretation of the hypothesized direction of the effect. For mathematical definitions of our models, see the Supplementary Methods section in the SI.

Results are presented as model predictions as well as counterfactual simulations of the outcomes under different conditions, taking into account whole posterior distributions of model parameters. All analyses were carried out in RStudio software (v4.4.1)^71^ and following packages were used to process the data and to prepare the figures: *ape* (v5.8)^72^; *geiger* (v2.0.11)^73^; *phytools* (v2.3.0)^74^; *maps* (v3.4.3)^75^; and *tidyr* (v1.3.1)^76^. The data and the code associated with this study can be accessed through ref. ^77^.

### Reporting summary

Further information on research design is available in the Nature Portfolio Reporting Summary linked to this article.

## Supplementary information

Supplementary Information

Reporting Summary

Peer Review file

## Data Availability

The celibacy data generated in this study using eHRAF World Cultures database^41^ and the existing data from the D-PLACE database^63^ used in this study have been deposited in the Figshare database under accession code https://doi.org/10.6084/m9.figshare.30346969.

## Code availability

The custom R code generated in this study has been deposited in the Figshare database under accession code: https://doi.org/10.6084/m9.figshare.30346969.
