# Supplementary material for: Religious celibacy is linked to marriage and wealth transmission practices across different world religions
Source: Nat Commun. 2026 Sep 29;17:11230. doi: 10.1038/s41467-026-78153-0 (PMC13720115; doi:10.1038/s41467-026-78153-0)
Supplement: Supplementary file 1 [file 41467_2026_78153_MOESM1_ESM.pdf]

Supplementary Information

for

Religious celibacy is linked to marriage and wealth transmission practices across different world religions

Gabriel Šaffa, Sara Sherbaji, Ruth Mace[*]

[*]Corresponding author (r.mace@ucl.ac.uk)

Supplementary Table 1. Summaries of posterior distributions of total-effect estimates for all-societies (N = 333) and sex-specific celibacy (N = 44) samples. Each posterior is represented by its mean, 90% credibility intervals (CI) and the percentage of posterior probability on the expected side of zero (% PP). Estimates are on the log odds scale.

predictor	mean	90% CI	% PP
celibacy			
marriage transactions	0.15	-0.42, 0.72	67.10
marital composition	1.23	0.59, 1.84	99.80
wealth inheritance	0.45	-0.16, 1.08	89.15
male celibacy			
transactions grom the groom's side	0.62	-0.07, 1.34	92.80
polygyny	0.29	-0.39, 0.97	75.60
patrilineal inheritance	0.61	-0.17, 1.35	90.35
female celibacy			
transactions from the bride's side	0.30	-0.39, 1.01	76.05
monogamy/polyandry	0.49	-0.22, 1.19	88.15

Supplementary Table 2. Summaries of posterior distributions of total-effect estimates for all-societies (N = 91) and sex-specific celibacy (N = 34) Eurasian samples. Each posterior is represented by its mean, 90% credibility intervals (CI) and the percentage of posterior probability on the expected side of zero (% PP). Estimates are on the log odds scale.

predictor	posterior mean	90% CI	% PP
celibacy			
marriage transactions	0.03	-0.63, 0.66	53.45
marital composition	0.57	-0.21, 1.30	89.25
wealth inheritance	0.45	-0.27, 1.16	84.55
male celibacy			
transactions grom the groom's side	0.49	-0.26, 1.22	85.85
polygyny	0.37	-0.35, 1.10	79.15
patrilineal inheritance	0.21	-0.58, 1.04	66.80
female celibacy			
transactions from the bride's side	0.53	-0.18, 1.25	88.45
monogamy/polyandry	0.46	-0.24, 1.20	84.80

Supplementary Table 3. Summaries of posterior distributions of total-effect estimates for all-societies (N = 333) and sex-specific celibacy (N = 44) samples stratified by religion – effect of marriage transactions. Each posterior is represented by its mean, 90% credibility intervals (CI) and the percentage of posterior probability on the expected side of zero (% PP). Estimates are on the log odds scale.

religion	posterior mean	90% CI	% PP
celibacy			
Indigenous	-1.10	-3.50, 0.18	14.55
Islam	-0.43	-2.38, 1.02	36.30
Christianity	0.63	-0.20, 1.82	84.00
Buddhism	0.10	-1.41, 1.78	53.65
Hinduism	-0.15	-1.67, 1.26	42.55
male celibacy			
Indigenous	-0.55	-3.95, 2.00	40.15
Islam	0.83	-1.56, 4.26	65.95
Christianity	0.73	-0.75, 2.70	74.55
Buddhism	2.10	-0.14, 5.99	90.70
Hinduism	0.90	-1.55, 4.51	67.05
female celibacy			
Indigenous	0.22	-1.27, 2.07	54.75
Islam	-0.03	-1.94, 1.82	49.70
Christianity	0.37	-0.63, 1.82	67.50
Buddhism	0.71	-0.31, 2.62	78.35
Hinduism	0.04	-1.42, 1.71	50.05

Supplementary Table 4. Summaries of posterior distributions of total-effect estimates for all-societies (N = 91) and sex-specific celibacy (N = 34) Eurasian samples stratified by religion – effect of marriage transactions. Each posterior is represented by its mean, 90% credibility intervals (CI) and the percentage of posterior probability on the expected side of zero (% PP). Estimates are on the log odds scale.

religion	posterior mean	90% CI	% PP
celibacy			
Indigenous	-1.03	-3.65, 0.27	18.40
Islam	-0.39	-2.30, 1.10	34.40
Christianity	0.57	-0.45, 2.25	75.20
Buddhism	0.21	-1.17, 1.84	57.70
Hinduism	-0.20	-1.75, 1.05	42.05
male celibacy			
Indigenous	-1.05	-5.63, 1.87	32.80
Islam	1.23	-1.63, 5.90	67.95
Christianity	1.72	-0.68, 6.08	82.05
Buddhism	2.86	-0.06, 7.30	93.05
Hinduism	1.36	-1.24, 5.55	73.35
female celibacy			
Indigenous	0.37	-1.20, 2.67	60.00
Islam	-0.02	-1.94, 1.92	50.10
Christianity	0.82	-0.41, 2.97	78.65
Buddhism	0.77	-0.32, 2.63	79.90
Hinduism	-0.05	-1.74, 1.44	49.10

Supplementary Table 5. Summaries of posterior distributions of total-effect estimates for all-societies (N = 333) and sex-specific celibacy (N = 44) samples stratified by religion – effect of marital composition. Each posterior is represented by its mean, 90% credibility intervals (CI) and the percentage of posterior probability on the expected side of zero (% PP). Estimates are on the log odds scale.

religion	posterior mean	90% CI	% PP
celibacy			
Indigenous	0.22	-0.78, 1.57	61.95
Islam	-0.13	-1.53, 1.00	44.25
Christianity	0.33	-0.34, 1.37	75.80
Buddhism	0.29	-0.63, 1.81	63.60
Hinduism	-0.14	-1.74, 1.01	45.80
male celibacy			
Indigenous	-0.42	-3.53, 1.87	43.00
Islam	0.78	-1.14, 3.89	67.15
Christianity	-0.69	-2.67, 0.68	25.70
Buddhism	1.71	-0.13, 5.22	89.15
Hinduism	0.51	-1.87, 3.71	58.70
female celibacy			
Indigenous	0.36	-0.77, 2.35	61.65
Islam	-0.03	-1.58, 1.60	48.15
Christianity	0.38	-0.63, 1.91	68.45
Buddhism	0.28	-0.66, 1.63	63.80
Hinduism	0.20	-1.05, 1.84	57.35

Supplementary Table 6. Summaries of posterior distributions of total-effect estimates for all-societies (N = 91) and sex-specific celibacy (N = 34) Eurasian samples stratified by religion – effect of marital composition. Each posterior is represented by its mean, 90% credibility intervals (CI) and the percentage of posterior probability on the expected side of zero (% PP). Estimates are on the log odds scale.

religion	posterior mean	90% CI	% PP
celibacy			
Indigenous	-0.66	-2.56, 0.44	25.60
Islam	-0.27	-2.24, 1.10	39.75
Christianity	0.16	-0.78, 1.32	58.85
Buddhism	0.60	-0.44, 2.51	72.70
Hinduism	-0.33	-2.15, 0.83	38.55
male celibacy			
Indigenous	-0.55	-4.06, 1.99	40.40
Islam	0.86	-1.27, 4.23	67.45
Christianity	0.69	-1.42, 3.74	62.80
Buddhism	1.88	-0.15, 5.44	88.00
Hinduism	0.64	-1.56, 3.85	63.55
female celibacy			
Indigenous	0.23	-1.09, 2.13	55.55
Islam	0.02	-1.47, 1.53	50.35
Christianity	0.47	-0.41, 2.02	72.75
Buddhism	0.29	-0.60, 1.69	64.40
Hinduism	0.13	-1.03, 1.68	56.15

Supplementary Table 7. Summaries of posterior distributions of total-effect estimates for all-societies (N = 333) and sex-specific celibacy (N = 44) samples stratified by religion – effect of wealth inheritance. Each posterior is represented by its mean, 90% credibility intervals (CI) and the percentage of posterior probability on the expected side of zero (% PP). Estimates are on the log odds scale.

religion	posterior mean	90% CI	% PP
celibacy			
Indigenous	-0.06	-0.98, 0.77	44.75
Islam	0.04	-1.01, 1.20	52.05
Christianity	0.04	-0.82, 1.02	50.10
Buddhism	0.01	-1.05, 1.19	50.15
Hinduism	0.04	-0.97, 1.14	52.20
male celibacy			
Indigenous	-1.02	-5.15, 1.83	32.10
Islam	1.11	-1.41, 4.99	70.60
Christianity	2.01	-0.15, 5.27	90.80
Buddhism	1.44	-0.74, 4.65	81.70
Hinduism	1.29	-1.46, 5.48	72.75

Supplementary Table 8. Summaries of posterior distributions of total-effect estimates for all-societies (N = 91) and sex-specific celibacy (N = 34) Eurasian samples stratified by religion – effect of wealth inheritance. Each posterior is represented by its mean, 90% credibility intervals (CI) and the percentage of posterior probability on the expected side of zero (% PP). Estimates are on the log odds scale.

religion	posterior mean	90% CI	% PP
celibacy			
Indigenous	-0.04	-1.49, 1.24	49.05
Islam	0.07	-1.37, 1.58	51.95
Christianity	0.57	-0.46, 2.61	71.30
Buddhism	0.09	-1.34, 1.73	55.35
Hinduism	0.01	-1.47, 1.58	49.70
male celibacy			
Indigenous	-0.75	-3.97, 1.27	34.00
Islam	0.69	-1.17, 3.84	63.75
Christianity	0.74	-0.90, 3.30	71.80
Buddhism	1.05	-0.54, 3.78	77.85
Hinduism	0.86	-1.05, 4.19	69.50

Supplementary Fig. 1: Ordered effects of marital composition on celibacy for all-societies sample (N = 333). **a** Posterior distribution of the estimate for the maximum effect of marital composition (β_M). **b** Incremental effects (δ) of marital composition, i.e., transitions from polygyny to monogamy and from monogamy to polyandry. Black vertical lines indicate 90% credibility intervals obtained by averaging over the estimates' posterior distributions, each containing 2000 parameter values. Abbreviations: polyg. – polygyny; monog. – monogamy; polya. – polyandry.

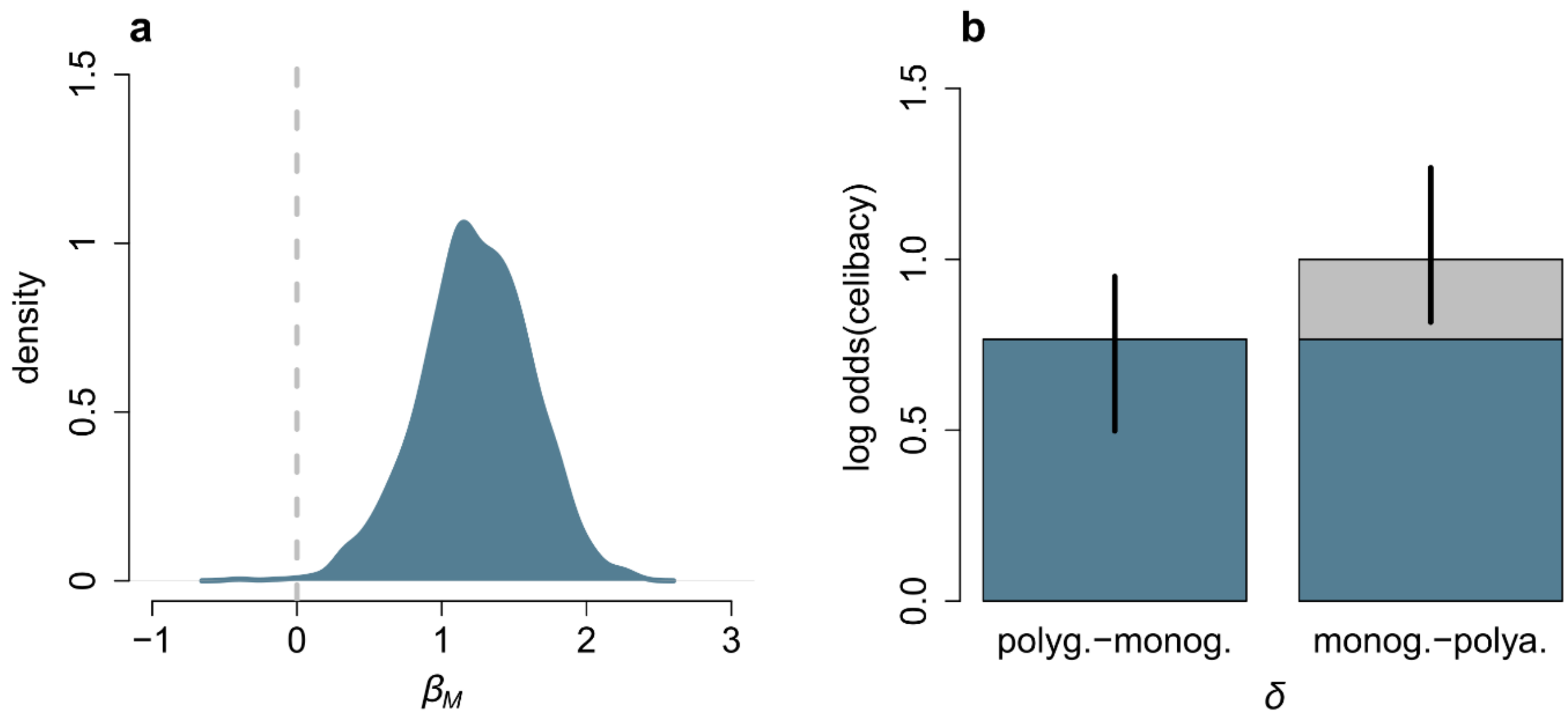

Supplementary Fig. 2: Categorical effects of marital composition on celibacy for all-societies sample (N = 333). Open points indicate posterior means, while horizontal lines are 90% credibility intervals obtained by averaging over the estimates' posterior distributions, each containing 2000 parameter values. Estimates are on the log odds scale.

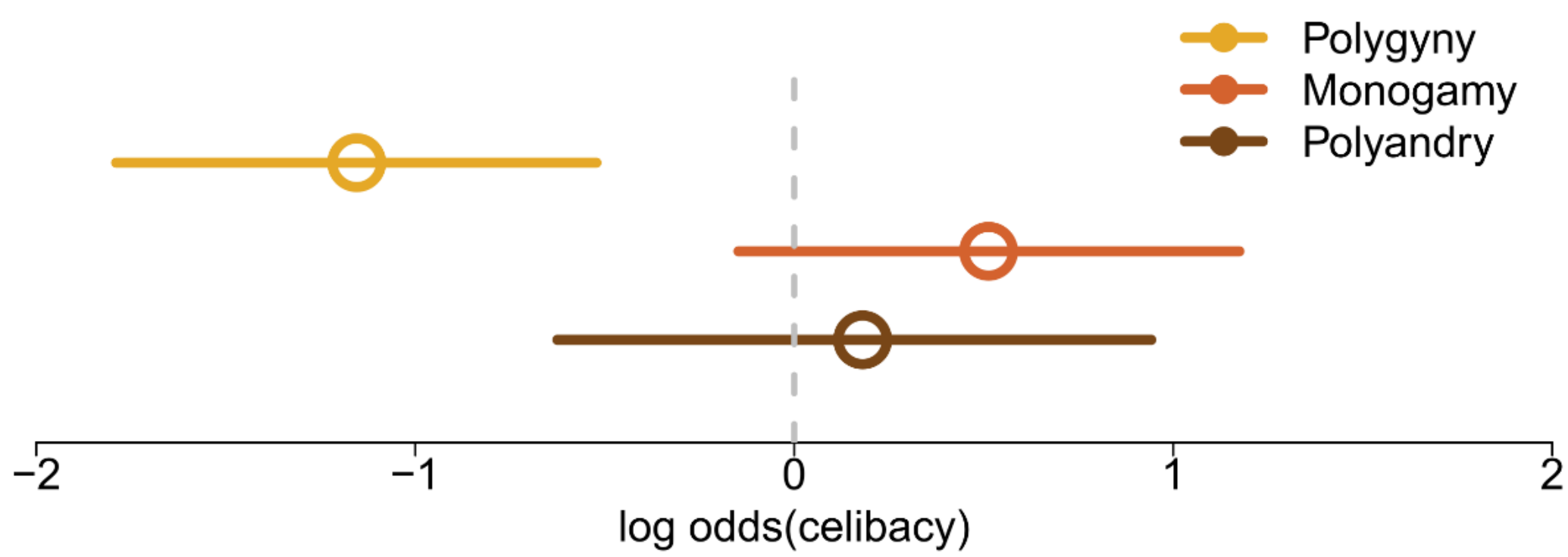

Supplementary Fig. 3: Model predictions for total-effect estimates for all-societies (N = 91) and sex-specific celibacy (N = 34) Eurasian samples. a-c Celibacy (cyan); **d-f** Male celibacy (yellow); **g-h** Female celibacy (red). Open points indicate posterior means and vertical lines are 90% credibility intervals obtained by averaging over the estimates' posterior distributions, each containing 2000 parameter values. The plot **b** shows estimates from the model where marital composition was modeled as a categorical variable. All plots are on the probability scale (vertical axis). The very small effects in plots **a-c** are due to a very low probability of celibacy in all-societies sample, captured by intercept parameters. Therefore, these plots are on a 0-0.5 scale. Abbreviations: polyg. – polygyny; monog. – monogamy; polya. – polyandry.

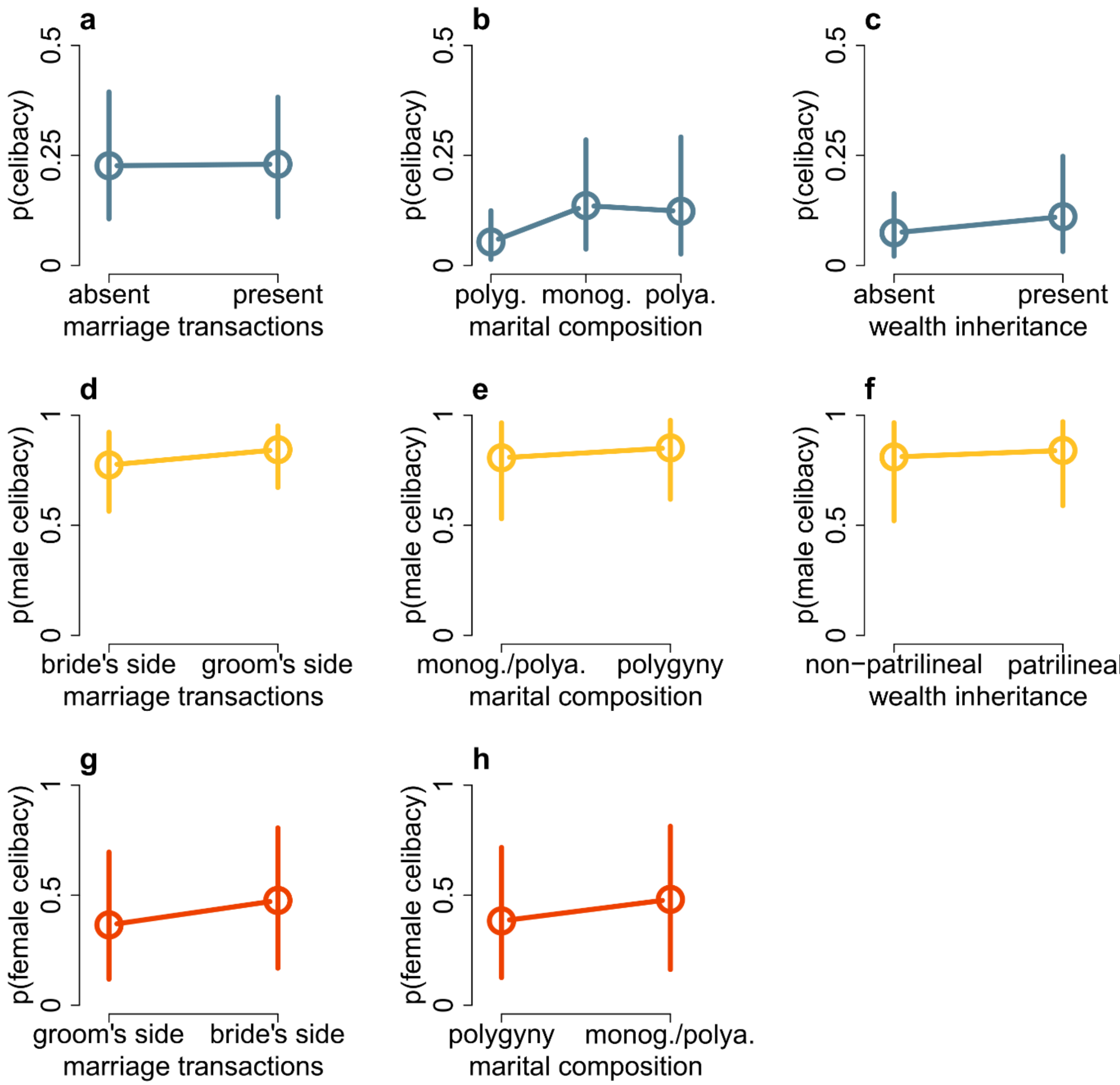

Supplementary Fig. 4: Ordered effects of marital composition on celibacy for Eurasian sample (N = 91). a Posterior distribution of the estimate for the maximum effect of marital composition (β_M). **b** Incremental effects (δ) of marital composition, i.e., transitions from polygyny to monogamy and from monogamy to polyandry. Black vertical lines indicate 90% credibility intervals obtained by averaging over the estimates' posterior distributions, each containing 2000 parameter values. Abbreviations: polyg. – polygyny; monog. – monogamy; polya. – polyandry.

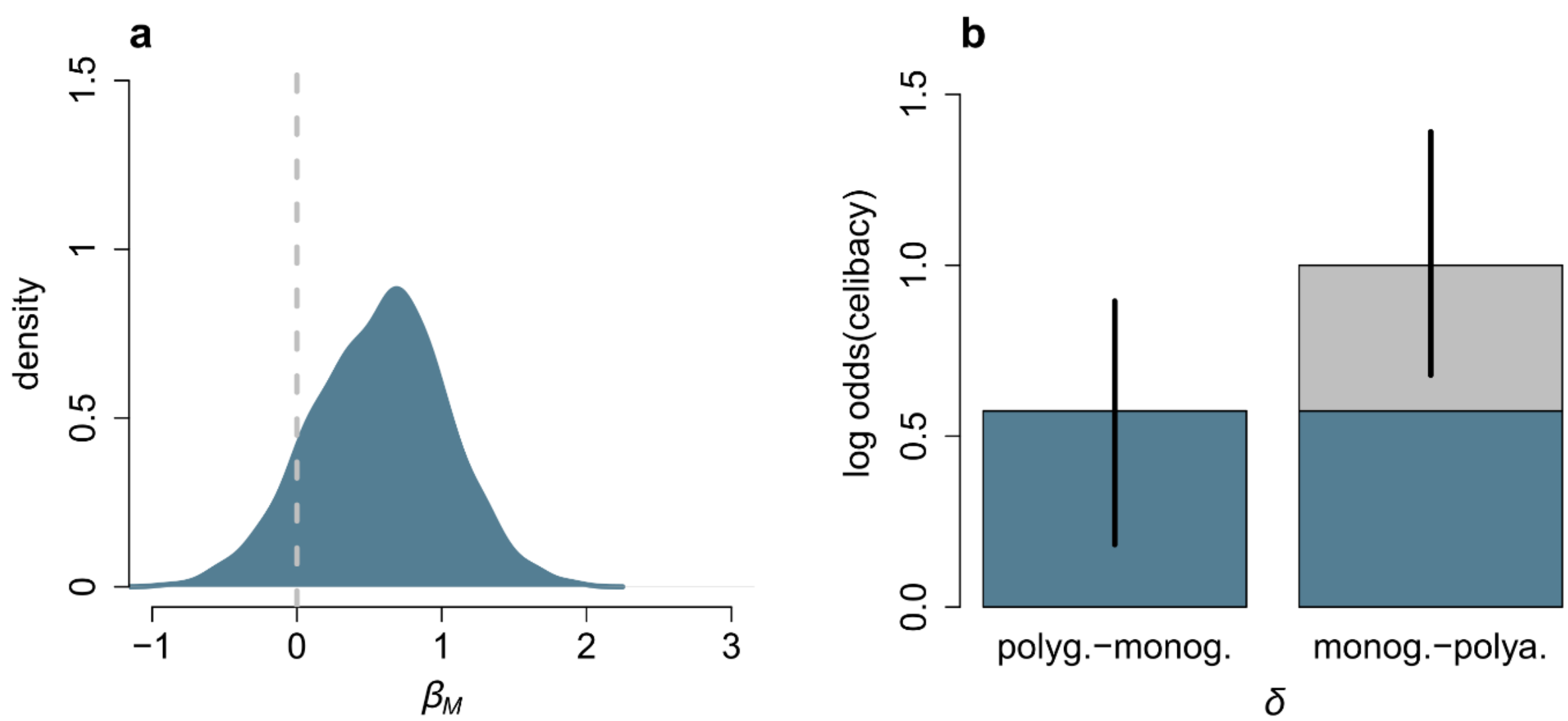

Supplementary Fig. 5: Categorical effects of marital composition on celibacy for Eurasian sample (N = 91). Open points indicate posterior means, while horizontal lines are 90% credibility intervals obtained by averaging over the estimates' posterior distributions, each containing 2000 parameter values. Estimates are on the log odds scale.

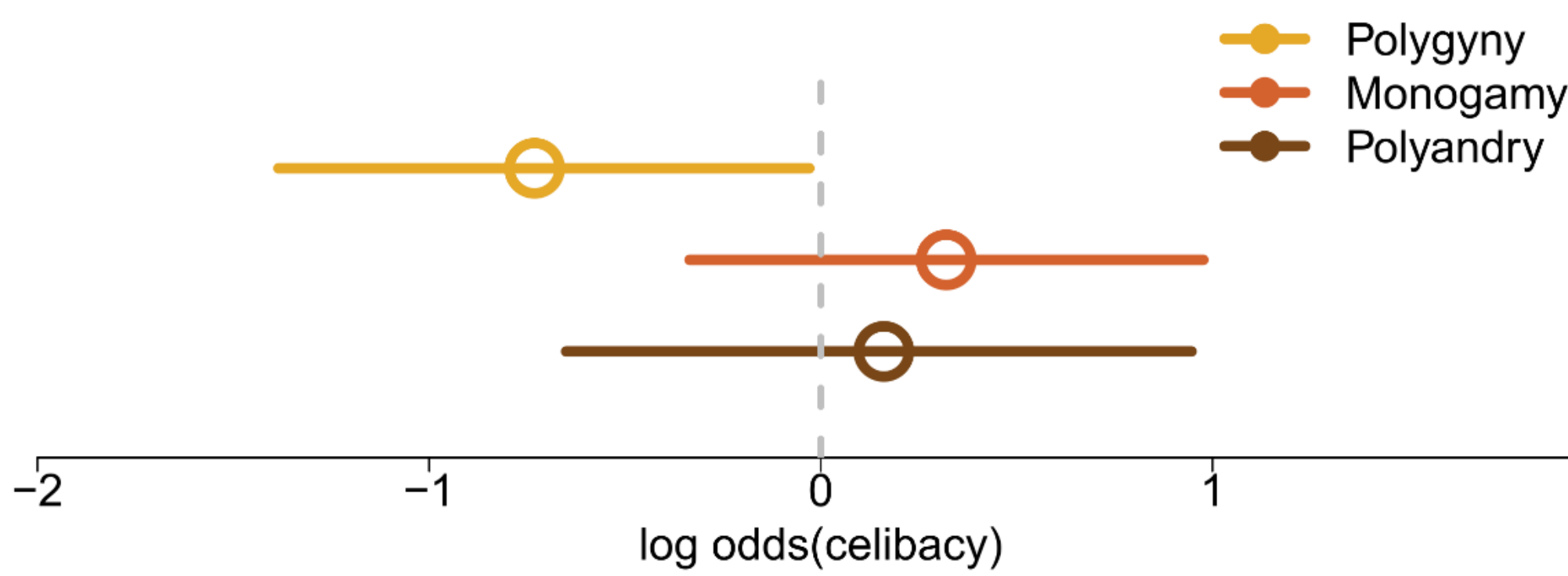

Supplementary Fig. 6: Categorical effects of marital composition on male celibacy for sex-specific celibacy sample (N = 44). Open points indicate posterior means, while horizontal lines are 90% credibility intervals obtained by averaging over the estimates' posterior distributions, each containing 2000 parameter values. Estimates are on the log odds scale.

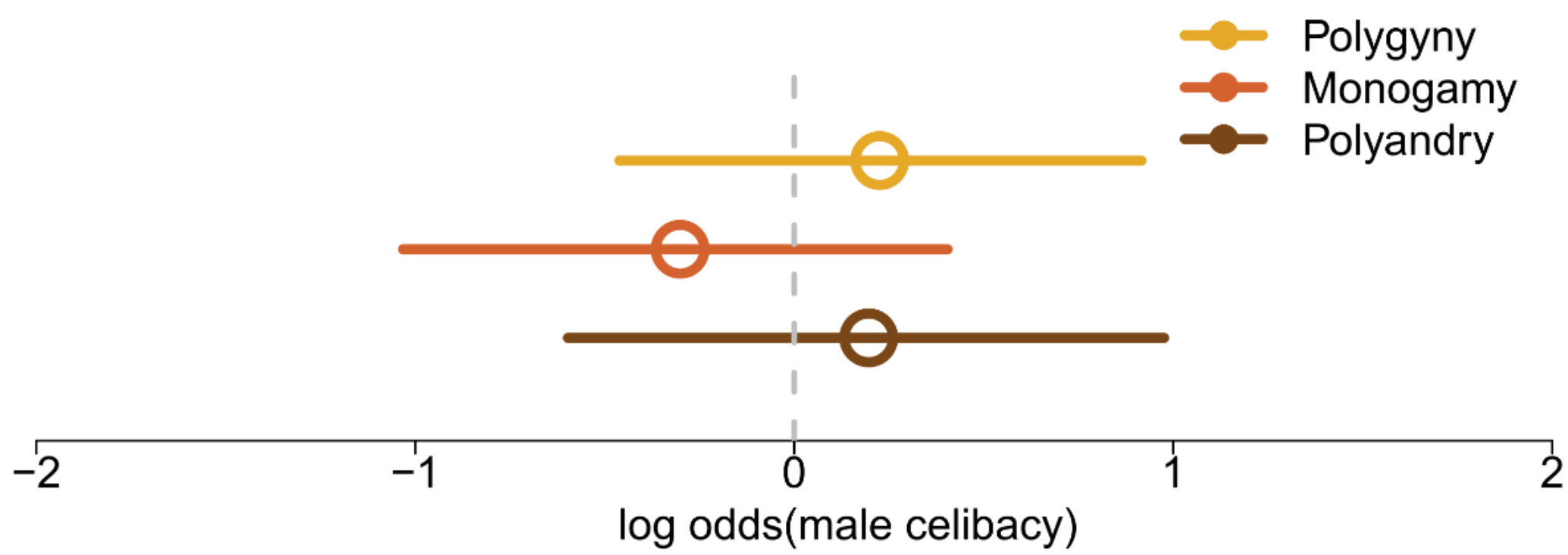

Supplementary Fig. 7: Categorical effects of marital composition on male celibacy for sex-specific celibacy Eurasian sample (N = 34). Open points indicate posterior means, while horizontal lines are 90% credibility intervals obtained by averaging over the estimates' posterior distributions, each containing 2000 parameter values. Estimates are on the log odds scale.

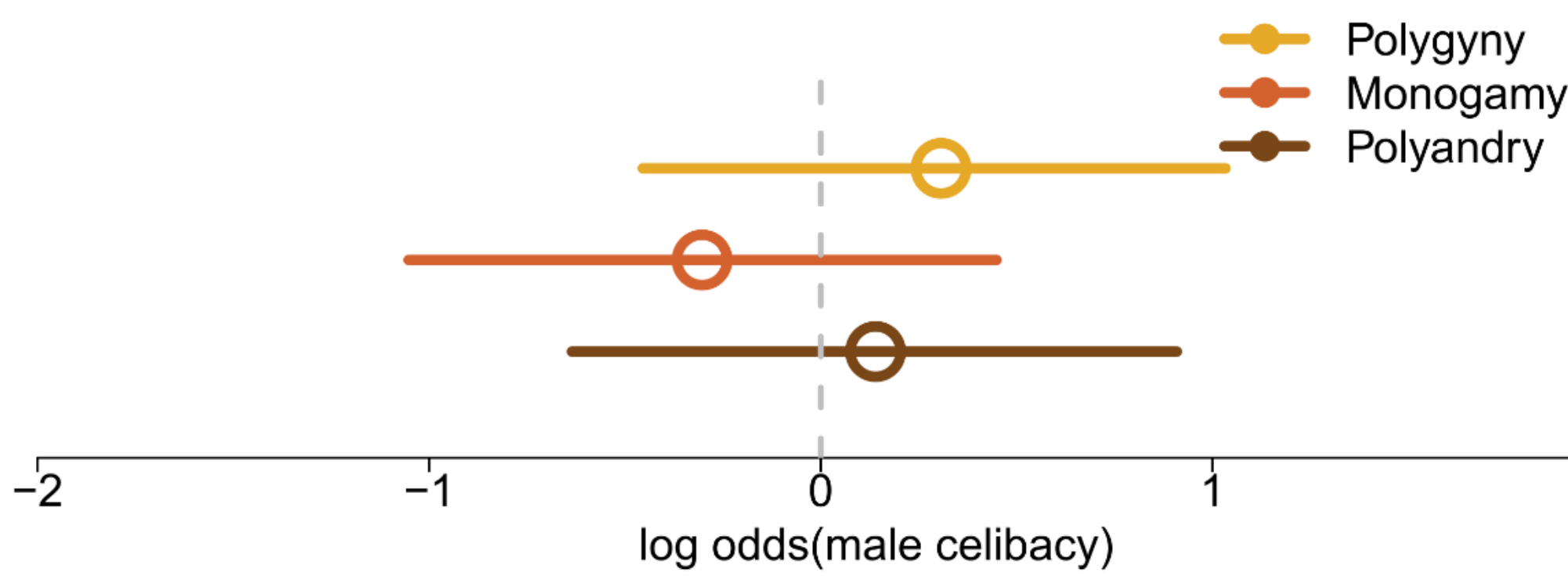

Supplementary Fig. 8: Categorical effects of marital composition on female celibacy for sex-specific celibacy sample (N = 44). Open points indicate posterior means, while horizontal lines are 90% credibility intervals obtained by averaging over the estimates' posterior distributions, each containing 2000 parameter values. Estimates are on the log odds scale.

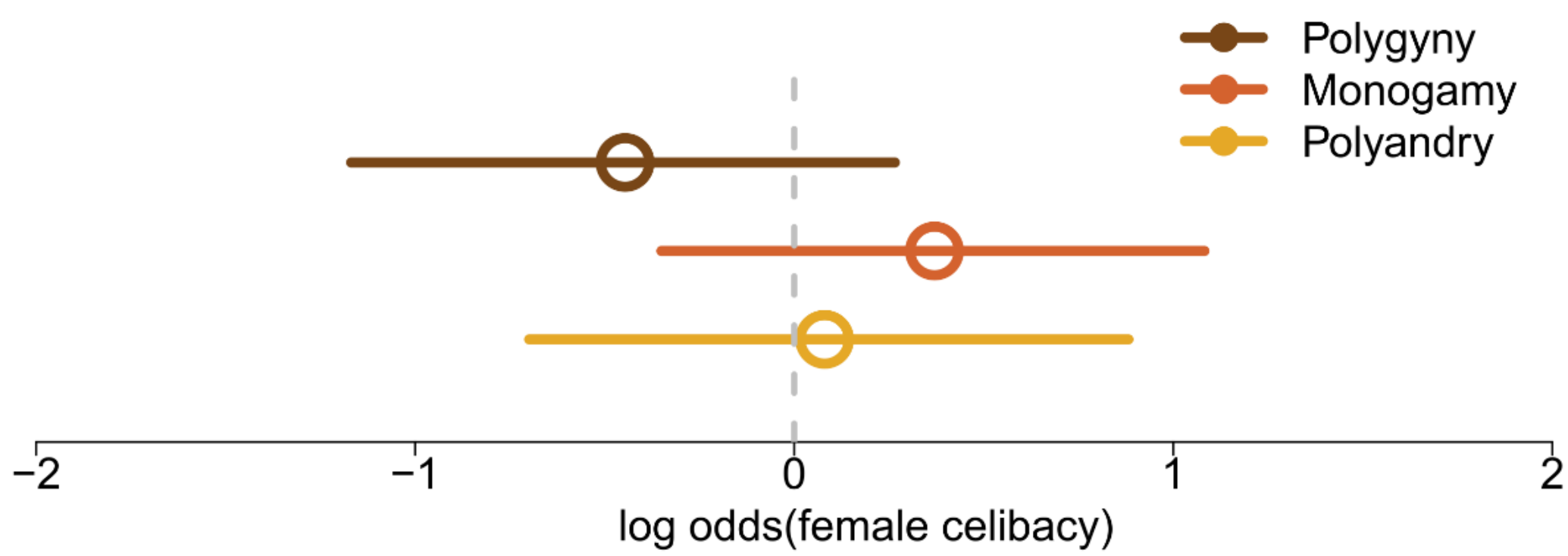

Supplementary Fig. 9: Categorical effects of marital composition on female celibacy for sex-specific celibacy Eurasian sample (N = 34). Open points indicate posterior means, while horizontal lines are 90% credibility intervals obtained by averaging over the estimates' posterior distributions, each containing 2000 parameter values. Estimates are on the log odds scale.

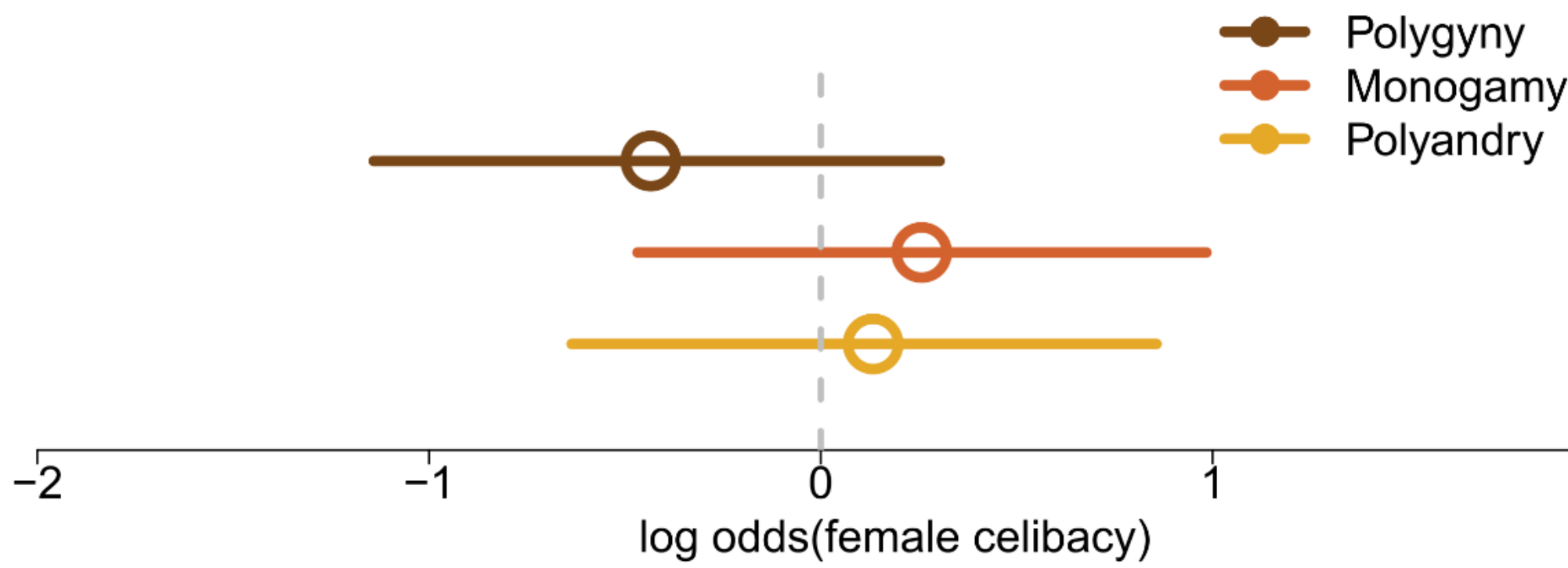

Supplementary Fig. 10: Posterior distributions of variation components for all-societies (N = 333) and sex-specific celibacy (N = 44) samples. a-c Celibacy; **d-f** Male celibacy; **g-h** Female celibacy. σ_a: variation in the log odds of (sex-specific) celibacy across religions (intercepts); σ_b: variation in the effect of the tested predictor on the log odds of (sex-specific) celibacy across religions (slopes); σ_{phy}: variation in the log odds of (sex-specific) celibacy due to phylogenetic relatedness. Values within the plots denote posterior means. All plots in the panel are on the log odds scale.

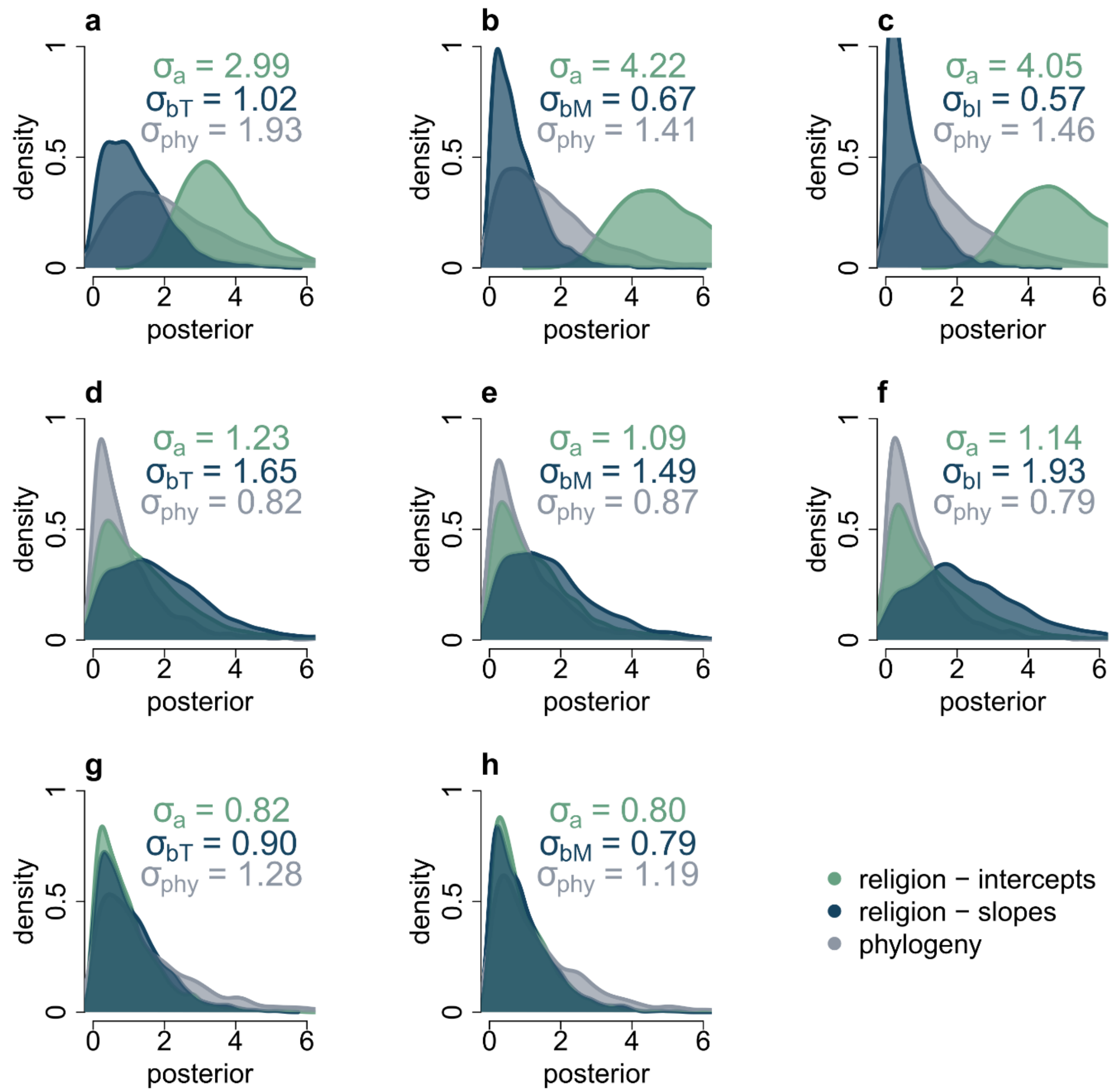

Supplementary Fig. 11: Posterior distributions of variation components for all-societies (N = 91) and sex-specific celibacy (N = 34) Eurasian samples. a-c Celibacy; **d-f** Male celibacy; **g-h** Female celibacy. σ_a: variation in the log odds of (sex-specific) celibacy across religions (intercepts); σ_b: variation in the effect of the tested predictor on the log odds of (sex-specific) celibacy across religions (slopes); σ_{phy}: variation in the log odds of (sex-specific) celibacy due to phylogenetic relatedness. Values within the plots denote posterior means. All plots in the panel are on the log odds scale.

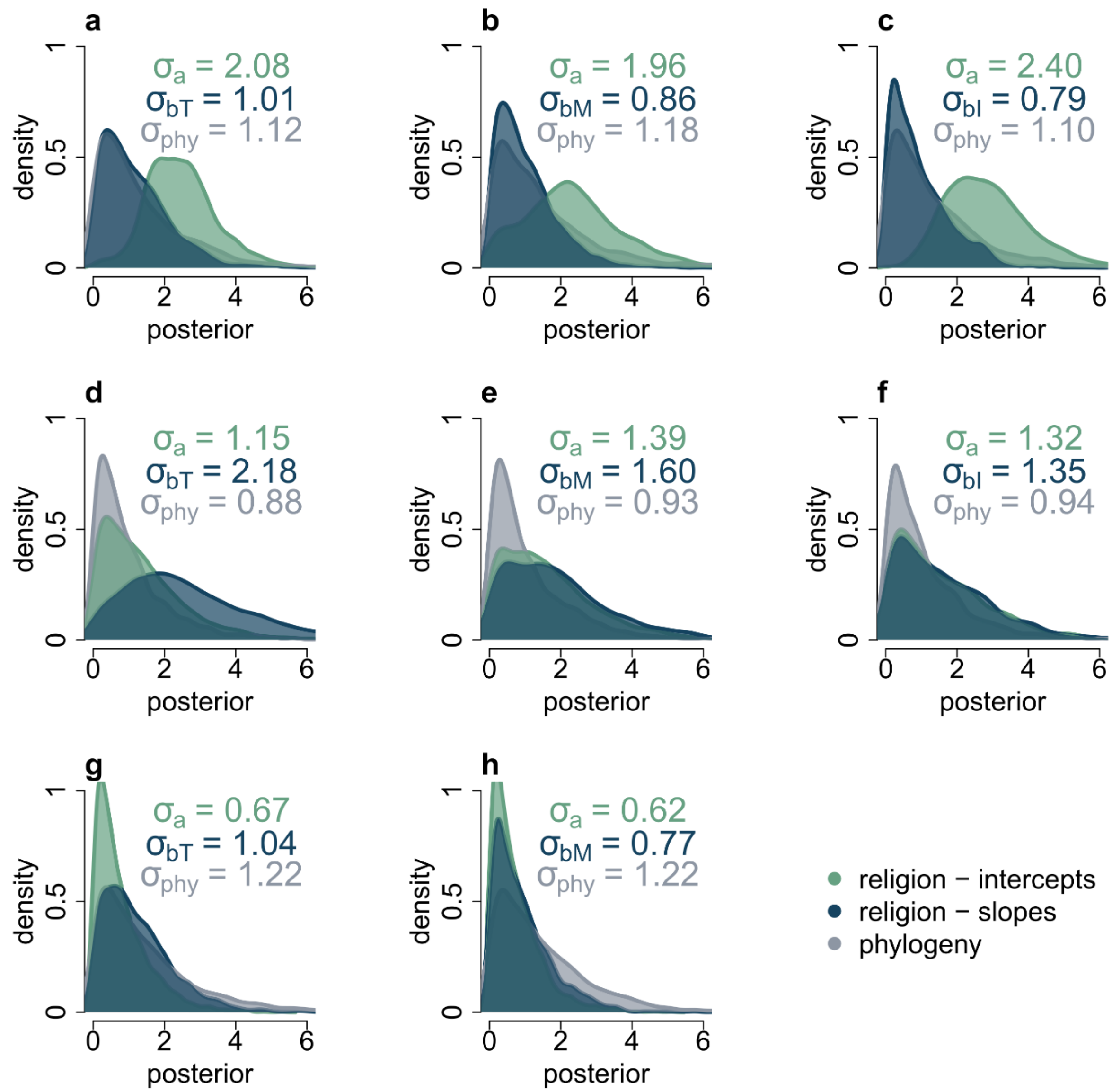

Supplementary Fig. 12: Distributions of celibacy rates in simulated societies based on models for all-societies (N = 333) and sex-specific celibacy (N = 44) samples. a-c Celibacy; **d-f** Male celibacy; **g-h** Female celibacy. Outcomes were simulated under different values (gray for absence; cyan for presence in models for celibacy; yellow for presence in models for male celibacy; red for presence in models for female celibacy) of each predictor, averaging over the estimates' posterior distributions, each containing 2000 parameter values. Celibacy distributions in plot **b** are simulated only under polygyny and polyandry, while omitting monogamy.

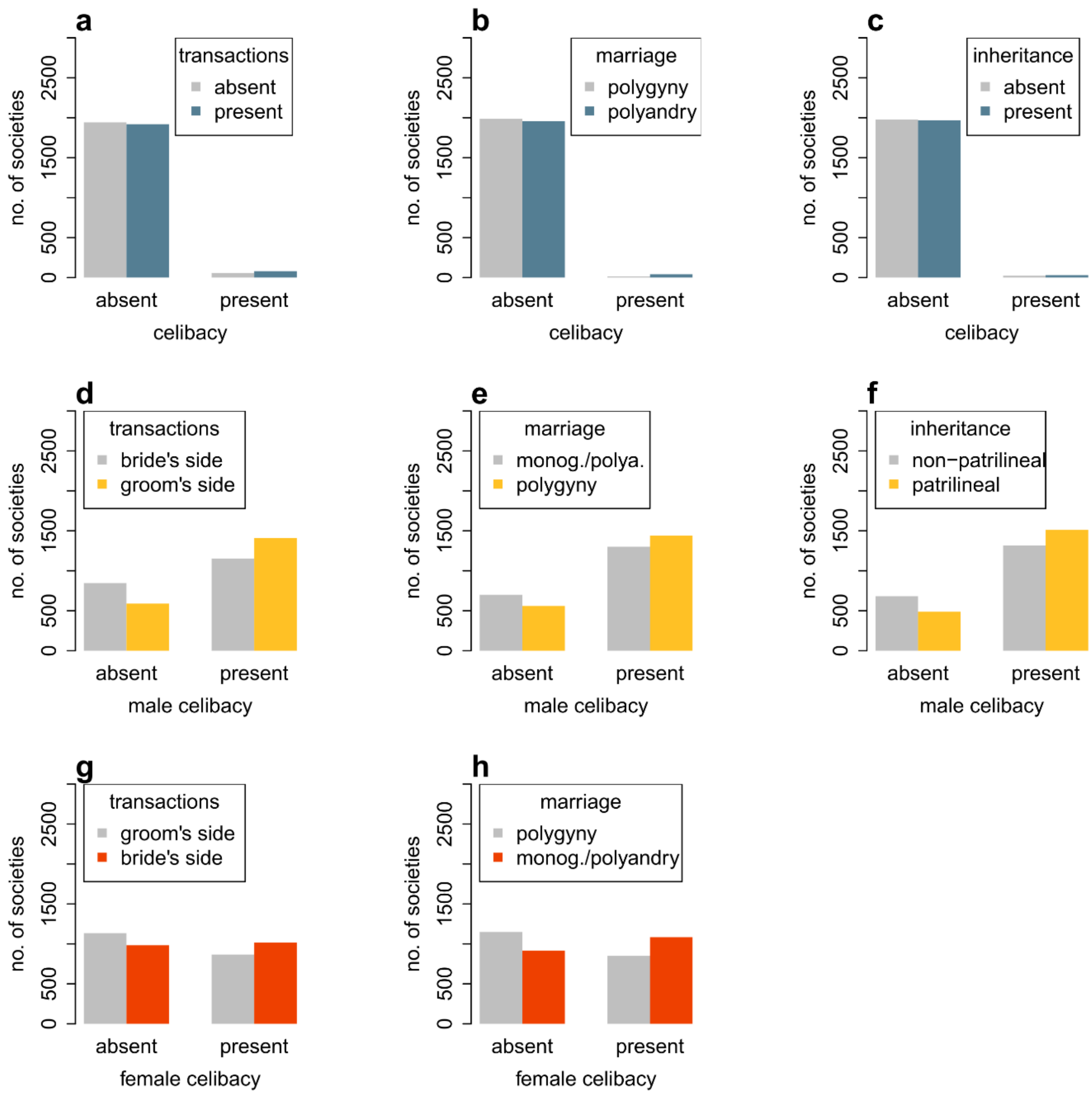

Supplementary Fig. 13: Distributions of celibacy rates in simulated societies based on models for all-societies (N = 91) and sex-specific celibacy (N = 34) Eurasian samples. a-c Celibacy; **d-f** Male celibacy; **g-h** Female celibacy. Outcomes were simulated under different values (gray for absence; cyan for presence in models for celibacy; yellow for presence in models for male celibacy; red for presence in models for female celibacy) of each predictor, averaging over the estimates' posterior distributions, each containing 2000 parameter values. Celibacy distributions in plot **b** are simulated only under polygyny and polyandry, while omitting monogamy.

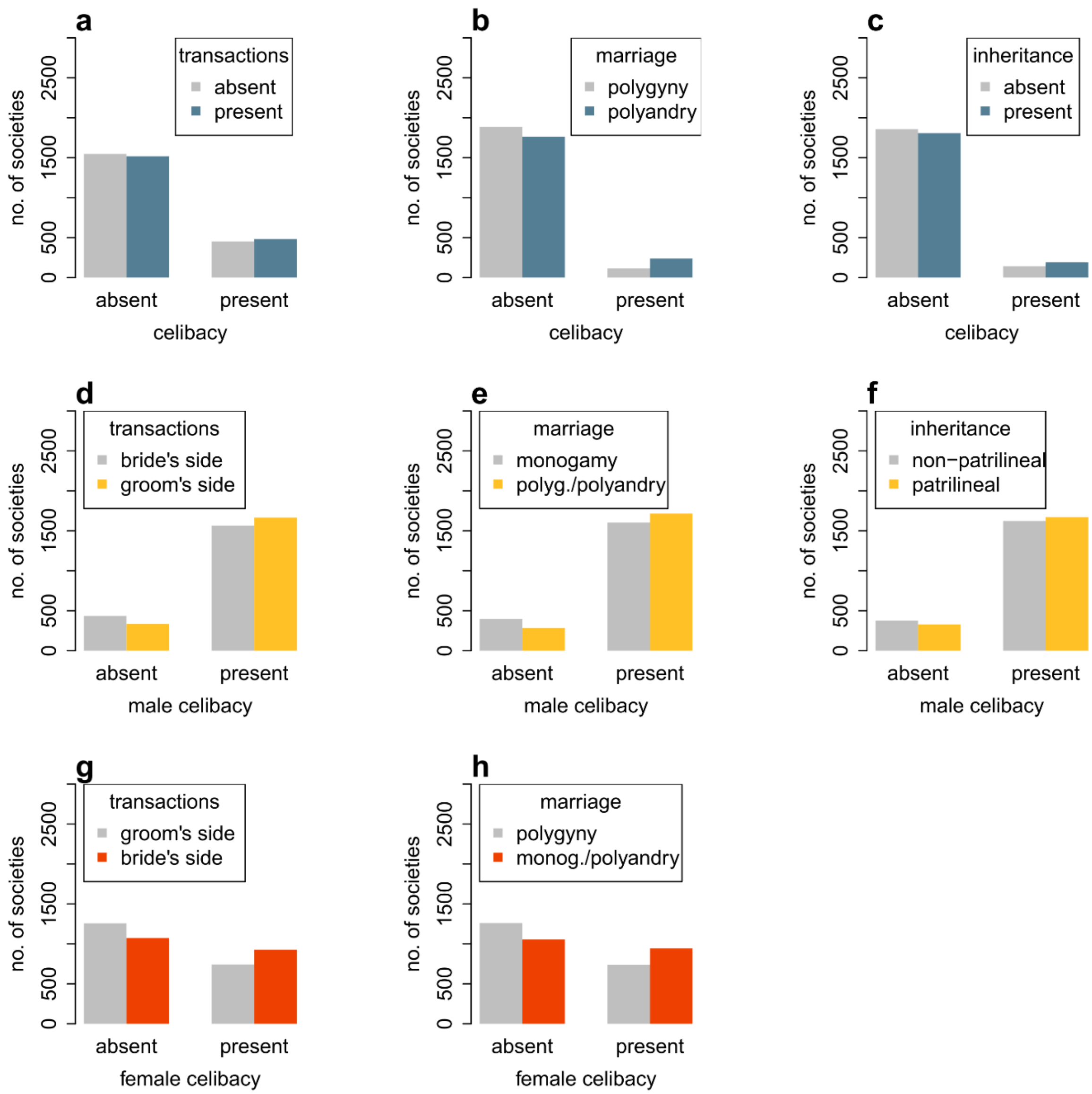

Supplementary Fig. 14: Distributions of celibacy rates in simulated Buddhist societies based on models for all-societies (N = 91) and sex-specific celibacy (N = 34) Eurasian samples. a-c Celibacy; **d-f** Male celibacy; **g-h** Female celibacy. Outcomes were simulated under different values (gray for absence; cyan for presence in models for celibacy; yellow for presence in models for male celibacy; red for presence in models for female celibacy) of each predictor, averaging over the estimates' posterior distributions, each containing 2000 parameter values. Celibacy distributions in plot **b** are simulated only under polygyny and polyandry, while omitting monogamy.

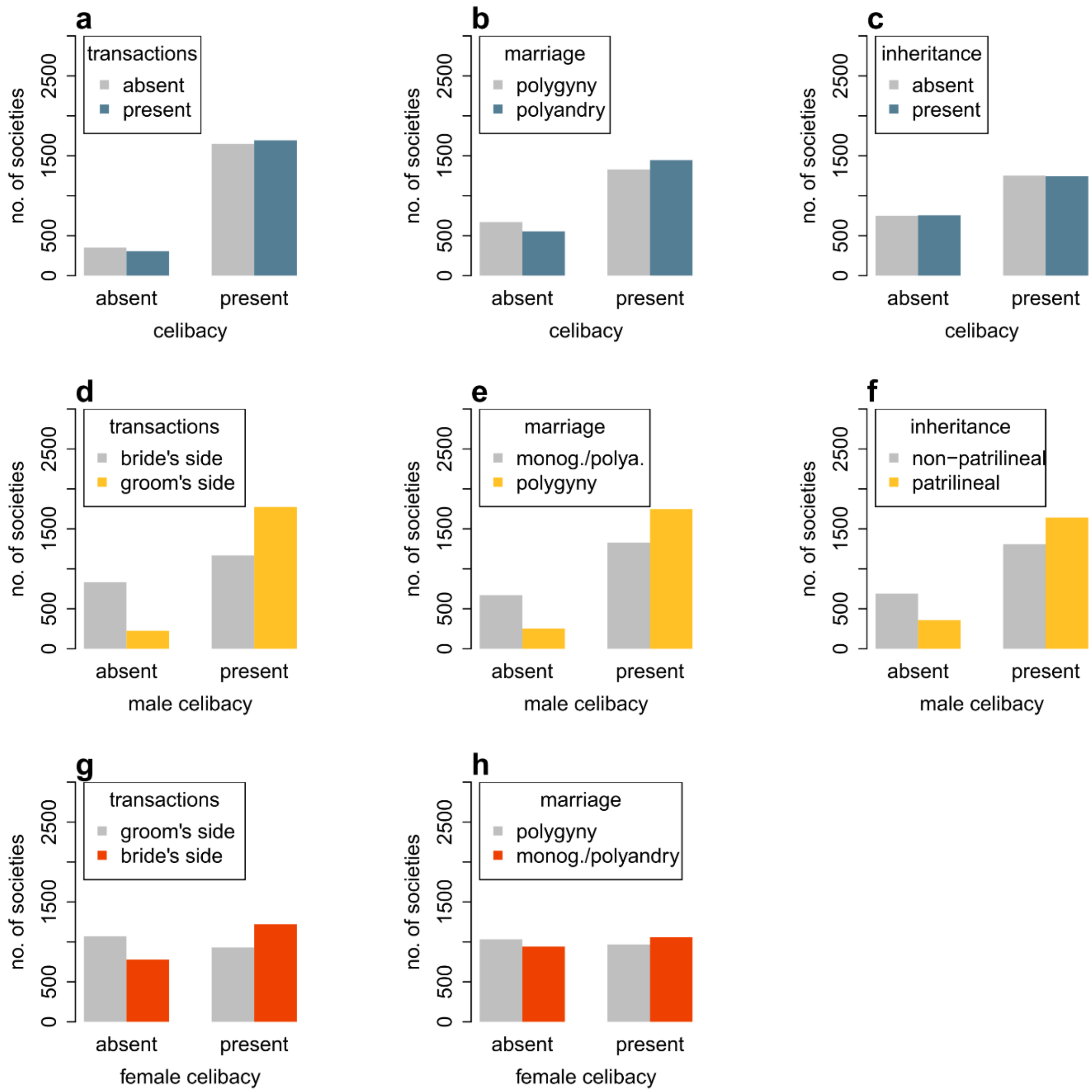

Supplementary Fig. 15: Distributions of celibacy rates in simulated Christian societies based on models for all-societies (N = 91) and sex-specific celibacy (N = 34) Eurasian samples. a-c Celibacy; **d-f** Male celibacy; **g-h** Female celibacy. Outcomes were simulated under different values (gray for absence; cyan for presence in models for celibacy; yellow for presence in models for male celibacy; red for presence in models for female celibacy) of each predictor, averaging over the estimates' posterior distributions, each containing 2000 parameter values. Celibacy distributions in plot **b** are simulated only under polygyny and monogamy, while omitting polyandry.

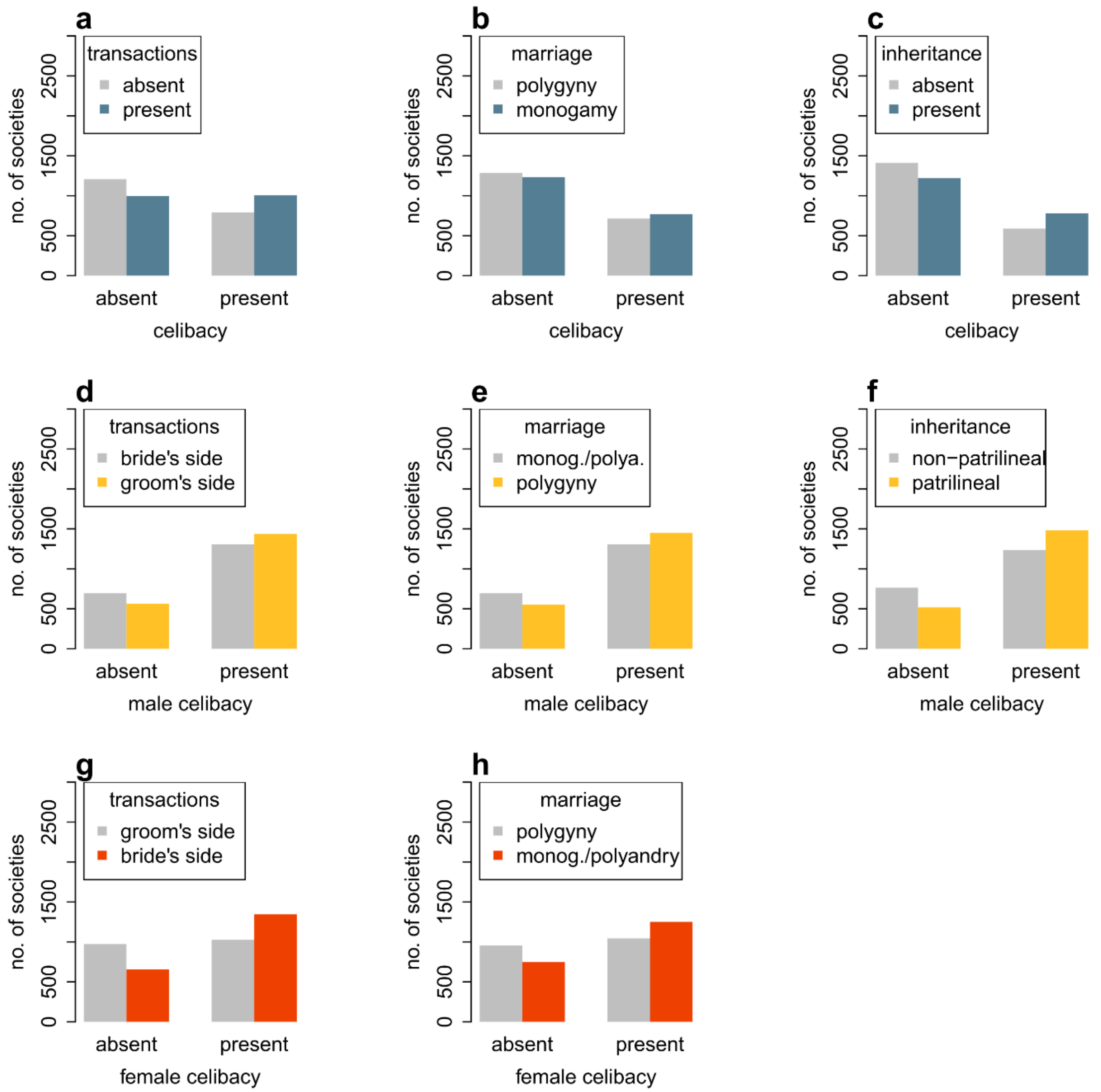

Supplementary Methods

1. Overview of the history and geography of religious celibacy

Religious celibacy potentially dates as far back as the 7th century BCE[1]. The Upaniṣad, a collection of Sanskrit texts that document the central religious concepts of Hinduism, describe small-scale monastic institutions that emerged in the forest districts of Northern India, centered around charismatic teachers. Buddhist and Jain monastic movements followed in the 6th and 5th centuries BCE, stemming from the same social milieu. In the Vinaya, texts of the Buddhist canon, the Buddha invites five followers to undertake the ascetic life and ordains them with the words 'Come, monk'[2]. Before his death, Vardhamana Mahāvāra, an older contemporary of the Buddha, brought together eleven Brahman followers (members of the priestly and scholarly class in ancient Indian society) who became heads of the first Jain monastic order[3]. The first evidence of Christian monasticism is documented in the 4th century CE Egyptian papyrus, which mentions the existence of monastic groups in Late Roman Egypt and Syria[4]. These communities were influenced by the practices of the 3rd century solitaries, such as St. Anthony and Simeon Stylites, who pioneered the Christian monastic lifestyle. In Europe, the earliest male and female monasteries emerged in the late 5th century CE[5]. Pockets of celibates emerged in other religions, such as Judaism (Essenes, Qumran, and Therapeutae, all described by Philo of Alexandria in the 1st century CE[6]), Islam (Sufis who lived in zāwāyā, lodges resembling medieval monasteries[1]), and Daoism (influenced by Buddhist practices in the 6th century CE[7]). However, celibacy was never institutionalized in Judaism and Islam as both religions strongly encourage 'multiplying'. For example, the Qur'an says: 'As for monasticism, it was invented by them [i.e., Christians]; We did not ordain it for them, but (they adopted it) to seek God's pleasure'[8]). The practice of celibacy was not only common to the major world religions. The Aztecs required celibacy from youth belonging to high-ranking families, where the practice distinguished them from commoners whose physical pleasures were considered a sign of inferiority[6]. These youth were enrolled in temples known as the 'calmecac' and trained as religious and military leaders[9]. They led austere lives, did not comb or wash their hair, were exempted from paying tax, and were provisioned by neighboring villagers[10].

Although celibacy is present in most of major religious traditions, there is considerable variation as to which sex predominates in the ranks of celibates. The preponderance of male or female celibates and their status in society varies not just between but also within religions, and across different time periods.

In Buddhism, monks are typically granted greater status and recognition than nuns[11]. Where rules of female Buddhist monastic ordination can be found (e.g., in the Bhikkhunī Pātimokkha, the Buddhist canon of rules for female Theravādin celibates[2]), they significantly out-number the corresponding male collection of rules, signifying the unequal status of male and female celibates in this religious system. However, while in Tibetan and Theravāda Buddhism, the main form of Buddhism in Sri Lanka, Myanmar, Laos, Cambodia and Thailand, nuns are rare compared to monks, in Vietnam, China, Korea and Japan, nuns are more common and respected[12,13,14].

In Hinduism, male celibates far outnumber female celibates. Women are largely excluded from observing the asramas (four stages of spiritual life, three of which feature celibacy[15]). Those who observe the asramas are almost exclusively male; they enter as students between the ages of eight and twelve and commit between nine to thirty-six years of their lives to the asramas. One sect of Hinduism, which departs from the majority, is the sect of Brahma Kumaris. This movement preaches that women have inherently low status due to their purportedly sexual nature, and that women can improve their status through celibacy; the movement is predominantly composed of women at both leadership and common levels[6].

In Christianity, while both men and women can pledge vows of celibacy to the Church, nuns were usually seen as lower status, and lived in greater isolation, than male celibates[16]. Nevertheless, medieval convents housed a significant number of Europe's aristocratic women[5]. In 16th century CE Florence, half of the city's elite families enrolled their daughters in convents[6]. During the 17th century CE, three percent of the population of Venice – three-thousand women – were cloistered nuns[6].

As mentioned, Islam generally rejects celibacy, but certain Sufi sects allow men to become celibate dervishes[17].

Other religions similarly revered celibacy for both men and women. For instance, the Aztecs, between the 14th and 16th centuries CE, dedicated both male and female children to the calmecac. Punishment for transgressing oaths of chastity was equally severe for both genders; violators were either strangled to death, set on fire, or shot by arrows[9].

Elsewhere, perpetual celibacy was expected only of women. For example, in the 2nd millennium BCE the Babylonians had celibate priestesses known as 'naditu'[18]. Kings appointed their daughters as high priestesses, indicating that celibacy was not a practice that arose out of a need to alleviate poverty. The naditu received inheritances from their fathers and were supported by male relatives throughout their time in the convent. They sometimes married but were forbidden to bear children; their biological sisters or concubines sometimes served as surrogates. Similarly, the 'aclla' women of the Inca, mainly in Cuzco, Peru between the 13th and 17th centuries CE, were sequestered women who were required to remain celibate, failure to do so being punishable by death[19]. There was no male equivalent among the Inca. The aclla entered the convent as early as age ten. They were not offered to the convents by their parents, but according to the will of the community and the rites of the religion. Another example are various Greco-Roman cults in antiquity, which required virgin priestesses to tend to their gods (e.g., hierodules or temple slaves) or protect the sacred fires of their cities (e.g., Vestal virgins[6]).

2. Data collection

A two-level search was conducted, spanning the Outline of Cultural Materials (OCM; i.e., subject codes used to index paragraphs on eHRAF), and included subcategories '791 Magicians and Diviners', '792 Prophets and Ascetics', '793 Priesthood', '794 Congregations', and '795 Religious Denominations'. An 'AND' operator was added between the subjects and keyword search, as well

as an ‘OR’ operator for the search terms. To extract paragraphs mentioning lifelong religious celibates, search terms included the following individual-based identifiers: monk, monks, lama, sufi, dervish, sanyasi*, sannyasi*, sadhu, nun, and nuns. Additionally, the search terms included the following institution-based titles: vihara, ashram, monaster*, lamaser*, and nunner*. The addition of the asterisk allows for plural variations of the term to appear in the results. A ‘NOT’ operator between search levels excluded paragraphs with OCM ‘797 Missions’. This served to exclude paragraphs with mentions of missionary celibates, for example, Orthodox Christian Russian missionaries in the Alutiiq society, which does not reflect any relationship between celibacy and the socio-ecological circumstances that could have given rise to it.

3. Coding

After the paragraphs were extracted (N = 6855), the following variables were coded: 1) presence or absence of celibacy, 2) sex of celibate, 3) predominant marital composition, 4) marriage transaction(s), and 5) religion to which celibates belonged. For each society, locality and focal year/period for each variable were recorded. For example, for Monguor, the focus of ethnographers was on the regions of Gansu and Qinghai in China between 1911 and 1922. In Korea, ethnographers recorded the presence of celibates in the Chung-Chong and Kangwon provinces between the end of the 19th century and the mid-20th century. Ethnographies of Cambodian celibates were mainly recorded in the Central Plains around the mid-20th century. As repeatedly stressed by anthropologists, the effective use of these databases is contingent on the careful matching of variables of interest with specific times and places[20,21,22,23], as practices tend to vary over time and across, often geographically proximate, groups. For each of these categories, evidence for every decision was recorded using specific paragraphs obtained from the eHRAF search. Examples of excerpts justifying these decisions are provided for each variable below.

Presence or Absence of Celibacy

All societies retrieved from the search were examined to determine the presence of celibacy. The objective was to exclude mentions that were detected in the search but did not accurately track the presence of celibacy in that society. For example, the search for the term ‘nun’ could not exclude words, such as ‘renunciate’ and ‘enunciate’. In some cases, the mention of the term ‘monk’ was in the context of a story told by informants about another society. In other cases, ‘monk’ came up as part of the word ‘monkey’. Paragraphs containing such terms were not considered and, as such, societies they were associated with were excluded. All remaining societies (N = 51) were thus recorded as ‘celibate’ (i.e., have some substantial form of lifelong religious celibacy). Note that while societies that were excluded did not return any evidence of celibacy from the search eHRAF, it is possible that celibacy may exist there and could be found in external sources. For the scope of this research, however, only eHRAF was consulted to determine this decision and only eHRAF societies were used to conduct this research.

Sex of Celibate

Societies with evidence of celibacy were further categorized by the sex of celibate. This variable comprised three categories:

Male celibates

This category refers to societies with exclusively or almost exclusively male celibates. For example, in some societies ethnographers observed that there were no female celibates, as in the case of Buryats of Siberia:

'The Buryat lamas abided strictly by the rules of celibacy, while the Outer Mongolian lamas were rather lax about it. Finally, there were no nuns among the Buryats, or the so-called Shabgantsas, i.e., old women who shaved their heads and took the vow of abstinence. Otherwise, the Buryat Lamaism was the same as that of Outer Mongolia'[24].

Female celibates

This category includes societies with exclusively or almost exclusively female celibates. For example, the Inca between the 13th and 17th centuries:

'Virgin women were secluded in Inca society; superficially, therefore, the acllas might appear to be equivalent to nuns, vestal virgins, or the women in a sultan's harem'[25].

Male and female celibates

This category refers to societies with both male and female celibates. These societies typically included repeated mentions of monks and nuns living closely in monasteries and convents. For example, ethnographers noted the presence of both monks and nuns among the Sherpa in the Khumba region during the 20th century:

'Sons and daughters who become monks or nuns receive their share of the property at the time they take monastic vows, which is thus structurally equivalent to marriage'[26].

Marital Composition

To determine marital composition of each society, a search on eHRAF was conducted using the OCM subcategory '595 Polygamy'. The search was restricted to societies that were confirmed celibate earlier in the coding process. This variable included the following categories:

Monogamy

This category refers to societies which are exclusively monogamous (i.e., where one man is only married to one woman at a time). While monogamy is and has always been the dominant marital composition in most societies around the world, exclusive – or normative – monogamy tends to be

a feature of Western Eurasian societies and strongly promoted by Christian religion. Take, for example, Arab Christians in Egypt and across the Middle East:

‘To the modern Egyptians, the monogamous Copts, as well as the polygamous Mohammedans, kind-hearted and full of feeling as they are, lively family affections are not to be denied’[27].

Polygyny

This category refers to societies without any legal restriction on the number of wives. No society is exclusively polygynous, however. Despite the widespread presence of polygyny – around 85% of societies – polygynous marriages comprise a minority of marriage arrangements within most societies. Polygyny often occurs among the wealthy, who can afford more than one wife, and sometimes in marriages where the first wife cannot bear a child or a son for her husband. Consider the practice of polygyny in Inner Mongolia, where both qualifications seem to be present:

‘Theoretically sons are more desirable than daughters, since males maintain the family line and inherit the wealth. Moreover, the fact that a husband sometimes resorts to polygyny when his first wife cannot give him a son, would seem to indicate that such a preference exists’[24].

‘Most Mongolian marriages are monogamous. Polygyny occurs and is, in fact, theoretically desirable, but few families are wealthy enough to be able to afford it’[24].

Polyandry

This category refers to societies where one woman is married to multiple men at the same time. Such societies are never exclusively polyandrous but also practice monogamy and often polygyny. Polyandry is practiced in less than 1% of ethnographically documented societies. For example, in Tibet, where all three marriage practices are found, the marriage system chosen by each family is usually one that addresses the specific socioecological problem the family is presented with. The nature of polyandry in Tibet is often fraternal (wherein one woman marries several brothers). As detailed in the literature review, the practice usually occurs to preserve wealth in the family when living conditions are harsh. For example, consider the practice among the Sherpa in the Solu-Khumbu region in eastern Nepal:

‘The land inheritance pattern is such that a father's land is divided equally among his sons, and theirs among their sons, theoretically ad infinitum. This policy, as one would imagine, tends to create postage-stamp fields. In the old days this problem could be solved by the practice of fraternal polyandry, in which all the sons, or several of them, married a single wife. The sons collectively held the land inherited from their father, and all the sons of their wife inherited the same undivided body of property. But the Nepalese government has outlawed polyandry (although there are still a few cases), and the problem is now handled in a variety of ways’[28].

Marriage Transactions

To determine the marriage transaction of each society, a search on eHRAF was conducted using the OCM subcategory '582 Mode of Marriage'. As before, the search was restricted to societies that were confirmed celibate earlier in the coding process. This variable was subjected to two levels of coding.

Level 1

Adapted from Huber et al.[29], this level of coding comprised the following categories:

Bride-service

Where the groom works for the bride's parents temporarily, this is considered bride-service. Bride-service can take place before or after the marriage, and often does so before. Bride-service usually takes place when the groom cannot provide the bride's parents with sufficient wealth; in other cases, it is a trial period before the formal ceremony takes place. This helps the bride's parents gauge whether the groom is suitable for their daughter, especially in cases where their daughter inherits land and where the groom is expected to tend to it. When giving their daughters to men of lower status, Kandayan fathers in Sri Lanka usually expect some form of bride-service from the groom:

'There are always enough poor men of the same wamsa [status] who would like binna [marriage between a lower status man and higher status woman] connections and there is no need for a wealthy man to have a binna son-in-law who is of low wamsa as well as being poor. Of course, reciprocity is maintained in binna marriages. The husband compensates for his poverty by his extra labor to his father-in-law'[30].

Groom-service

Where the bride works for the groom's parents temporarily, this is known as groom-service. Like bride-service, it often serves as an assessment of the bride's suitability within the marriage and family. In many cases, it serves as household training for the bride by the mother-in-law. Take for instance this arrangement in Korea:

'The family in Korea may be a much complicated organization, with its fathers, grandfathers, and great-grandfathers, to say nothing of the mothers, grandmothers, and the great-grandmothers, who spend their time in ruling with an iron hand the younger women of the household. Among the younger women are to be found the wives and concubines of the younger men of the family, and in many cases the prospective wives of the boys who are not yet married, but whose brides have been selected and placed in the family in order that they may be trained up in the way of absolute obedience to a mother-in-law'[31].

Bride-wealth

The transfer of wealth from the groom's side of the family to the bride's side of the family is known as bride-wealth. This is the most common practice among ethnographically documented societies,

found in nearly 52% of them. It is widespread in Africa, the Middle East, Eastern Europe (see the example below of bride-wealth in Central Serbia in the early 20th century), and elsewhere:

‘The father of a distinguished family told me that he bought one daughter-in-law for 6,000 dinars, and another for 4,000 dinars, while he was not charged anything for the third one. Speaking of the latter he said: “Cursed be the hour she entered my house. She has caused 100,000 dinars worth of damage and she will succeed in splitting the zadruga [extended family], the largest and richest in the whole district. If she had been worth any money, they would not have let her go without payment, for something worthwhile is worth paying for.” The remark of this father indicates that the custom of bride buying was very old and widespread. Otherwise, he would not have considered it normal and justified to sacrifice such large sums. He had paid for two daughters-in-law as much as for about four or five head of cattle, and this was a large amount for a Balkan peasant in a period of a serious agricultural crisis. At that time 1,000 dinars was worth about 18 dollars’[32].

Groom-wealth

This refers to the transfer of wealth from the bride’s side of the family to the groom’s side of the family. This form of marriage transaction is much less common than bride-wealth, with less than 4% of societies engaging in this practice. It is mostly found across Europe and post-colonial nations such as India, of which the following is an example:

‘Brahmans have to pay large dowries, whether they are able or no: the least sum being Rs. 1,000 for a bridegroom whose educational qualifications are measured by the Matriculation examination. Even ‘ne'er-do-weels’ get a high dowry owing to the unsatisfied demand for bridegrooms’[33].

Dowry from bride’s parents

This refers to the transfer of wealth from the bride’s parents to the couple or – as is more often the case – to the bride. It is often used to help the couple establish themselves. Whereas this category is sometimes confused with groom-wealth, the distinguishing feature is the recipient of the wealth transfer. In the case of groom-wealth, it is the family of the groom. Dowry from the bride’s parents often constitutes what would have been given as inheritance to the daughter (e.g., land or cattle), or can be as small as a trousseau (i.e., material items, sometimes prepared by the bride herself with the help of her family, such as clothing or furniture). Consider this example from the Greek society:

‘A couple set up house with the bride's dowry (a house given to her by her parents, its furniture and fittings, and possibly a few grain terraces and olive trees) but the groom will not get the land which is his share of his natal family's estate until his parents die’[34].

Dowry from groom’s parents

This refers to the transfer of wealth from the groom’s parents to the couple or to the groom. Again, it is often used to help the couple establish themselves. See, for example, the dowries brought by both husband and wife in an Amhara marriage in Dega Damot district of Gojjam province, Ethiopia, between the years 1961 and 1962:

'The Dowries Brought into Ayyälä's Marriage Ayyälä's movable property at marriage: 4 pair of oxen, 15 cows, 20 sheep, 12 horses, 3 mules, 4 male slaves (Nilots), 2 female slaves (Nilots). His wife brought into the marriage: 3 pairs of oxen, 10 cows, no sheep, 6 horses, 4 mules, 2 male servants (Nilots), 1 female servant (Nilot)'[35].

Gift exchange

This refers to the exchange of material items between parents and relatives from both sides. Consider this example from Tongans in Polynesia:

'The formality of a traditional marriage arrangement was marked by a customary set of reciprocal visits among prospective affines, including the exchanges of food, mats, and tapa, 7. See Collocott 1923b. but without any considerable movement of goods (as bridewealth or dowry) to either the husband's or wife's kinsmen'[36].

Level 2

Once one or more of the above values were selected for each society, the transactions were then grouped into one of the following categories:

From the groom's side

Any wealth transactions extending from the groom's side of the family to the couple, bride, groom, or the bride's kinsfolk. This included bride-service, bride-wealth, and dowry from the groom's parents.

From the bride's side

Any wealth transactions extending from the bride's side of the family to the couple, bride, groom, or the groom's kinsfolk. This included groom-service, groom-wealth, and dowry from the bride's parents.

Both sides

This included instances where any of the former two categories were present together, as well as gift exchange.

4. Statistical modeling

Our main Bayesian phylogenetic models were of the form:

$$\text{Celibacy}_\text{i} \sim \text{Bernoulli}(p_i)$$

$$\log \frac{p_i}{1 - p_i} = \alpha + \beta_x \text{X}_\text{i} + \beta_z \text{Z}_\text{i} + \text{phy}_\text{i}$$

and

$$\log\frac{p_i}{1-p_i} = \alpha_j + \beta_{x,j}\mathrm{X_i} + \beta_{z,j}\mathrm{Z_i} + \mathrm{phy_i}$$

$$\begin{pmatrix}\mathrm{phy_1}\\ \mathrm{phy_2}\\ \cdots\\ \mathrm{phy_n}\end{pmatrix} \sim \mathrm{MVNormal}\left(\begin{pmatrix}0\\ 0\\ \cdots\\ 0\end{pmatrix}, \mathbf{S}\right)$$

$$\mathbf{S} = \sigma_{phy}\mathbf{R}$$

$$\alpha \sim \mathrm{Normal}(0{,}1.5)$$

$$\alpha_j \sim \mathrm{Normal}(0, \sigma_\alpha)$$

$$\beta_j \sim \mathrm{Normal}(0, \sigma_\beta)$$

$$\sigma_\alpha \sim \mathrm{Exponential}(1)$$

$$\sigma_\beta \sim \mathrm{Exponential}(1)$$

$$\sigma_{phy} \sim \mathrm{Exponential}(1)$$

where $\mathrm{phy_i}$ are society-specific phylogenetic intercepts and j is a varying effect of religion. Phylogenetic correlations are captured in the off-diagonal elements of the matrix **R** scaled by σ_{phy}, giving a magnitude of the phylogenetic effect. The β_x denotes the total effect of $\mathrm{X_i}$, while β_z denotes the coefficient of any variable $\mathrm{Z_i}$ used to adjust the model for, as implied by the DAG.

References

1. Davis, S. J. *Monasticism: A very short introduction* (Oxford University Press, 2018).
2. Strong, J. *Buddhisms: An Introduction* (Oneworld, 2015).
3. Dundas, P. *The Jains* (Routledge, 2002).
4. Goehring, J. E. *Ascetics, society, and the desert: studies in early Egyptian monasticism* (Trinity Press International, 1999).
5. Hager, B. J. Get thee to a nunnery: Female religious claustration in medieval Europe. *Ethology and Sociobiology* **13**, 385–407 (1992).
6. Abbott, E. *A history of celibacy* (Da Capo Press, 2001).
7. Kohn, L. *Sexual Control and Daoist Cultivation*. In Celibacy and Religious Traditions 241–264 (Oxford University Press, 2007).
8. The Qur'an (M. A. S. Abdel Haleem, Trans., Oxford University Press, 2010).
9. Soustelle, J. *Daily life of the Aztecs* (Macmillan Publishers, 1962).
10. Qirko, H. N. *Induced altruism in the maintenance of institutionalized celibacy* (The University of Tennessee, 1998).
11. Spiro, M. E. *Gender hierarchy in Burma: cultural, social, and psychological dimensions.* In Sex and gender hierarchies 316–333 (Cambridge University Press, 1993).
12. Kieschnick, J. H. *Celibacy in East Asian Buddhism*. In Celibacy and religious traditions (eds. C. Olson) 225–240 (Cambridge University Press, 2007).
13. Powers, J. *Celibacy in Indian and Tibetan Buddhism*. In Celibacy and religious traditions (eds. C. Olson) 201–224 (Oxford University Press, 2007).
14. Micheletti, A. J., Ge, E., Zhou, L., Chen, Y., Zhang, H., Du, J., & Mace, R. Religious celibacy brings inclusive fitness benefits. *Proceedings of the Royal Society B* **289**, 20220965 (2022).
15. Olivelle, P. *Celibacy in Classical Hinduism*. In Celibacy and Religious Traditions (eds. C. Olson) 151–164 (Oxford University Press, 2007).
16. Gilchrist, R. *Gender and material culture: The archaeology of religious women* (Routledge, 1994).
17. Bashir, S. *Islamic tradition and celibacy*. In Celibacy and religious traditions (Oxford University Press, 2007).
18. Saggs, H. W. F. *The greatness that was Babylon: a survey of the ancient civilization of the Tigris- Euphrates Valley*. In Great civilization series (Sidgwick and Jackson, 1988).
19. de la Vega, G. *First part of the Royal commentaries of the Yncas* (Cambridge University Press, 2010).
20. Kirby, K. R., Gray, R. D., Greenhill, S. J., Jordan, F. M., Gomes-Ng, S., Bibiko, H. J., ... & Gavin, M. C. D-PLACE: A global database of cultural, linguistic and environmental diversity. *PloS one* **11**, e0158391 (2016).
21. Watts, J., Jackson, J. C., Arnison, C., Hamerslag, E. M., Shaver, J. H., & Purzycki, B. G. Building quantitative cross-cultural databases from ethnographic records: promise, problems and principles. *Cross-Cultural Research* **56**, 62–94 (2022).

22. Ember, C. R. Using the HRAF collection of ethnography in conjunction with the Standard Cross-Cultural Sample and the Ethnographic Atlas. *Cross-Cultural Research* **41**, 396–427 (2007).
23. Slingerland, E., Atkinson, Q. D., Ember, C. R., Sheehan, O., Muthukrishna, M., Bulbulia, J., & Gray, R. D. Coding culture: Challenges and recommendations for comparative cultural databases. *Evolutionary Human Sciences* **2**, e29 (2020).
24. Far Eastern and Russian Institute of the University of Washington. *Mongolian People's Republic (Outer Mongolia)*. In Human Relations Area Files Subcontractor's Monograph (Human Relations Area Files, 1956).
25. Silverblatt, I. *Moon, sun, and witches: Gender ideologies and class in Inca and colonial Peru* (Princeton University Press, 1987).
26. Paul, R. A. *The Tibetan symbolic world: psychoanalytic explorations* (University of Chicago Press, 1982).
27. Klunzinger, C. B. *Upper Egypt: its people and its products* (Scribner, Armstrong, & Co., 1878).
28. Ortner, S. B. *Sherpas through their rituals.* In Cambridge studies in cultural systems (Cambridge University Press, 1978).
29. Huber, B. R., Danaher, W. F., & Breedlove, W. L. New cross-cultural perspectives on marriage transactions. *Cross-Cultural Research* **45**, 339–375 (2011).
30. Yalman, N. *Under the bo tree: studies in caste, kinship, and marriage in the interior of Ceylon* (University of California Press, Ltd., 1971).
31. Moose, J. R. *Village life in Korea* (Publishing House of the ME Church, South, Smith & Lamar, agents, 1911).
32. Erlich, V. S. *Family in transition: a study of 300 Yugoslav villages* (Princeton University Press, 1966).
33. Slater, G. *Some south Indian villages.* In Economic studies (H. Milford, Oxford University Press, 1918).
34. Kenna, M. E. *The idiom of family*. In mediterranean family structures 347–362 (Cambridge University Press, 1976).
35. Hoben, A. *The role of ambilineal descent groups in Gojjam Amhara social organization* (University Microfilms, Inc., 1963).
36. Marcus, G. E. *The nobility and the chiefly tradition in the modern Kingdom of Tonga* (The Polynesian Society, 1978).
37. Mulder, N. *Inside Thai society: interpretations of everyday life* (The Pepin Press, 1996).
