# Supplementary material for: Religious celibacy is linked to marriage and wealth transmission practices across different world religions
Source: Nat Commun. 2026 Sep 29;17:11230. doi: 10.1038/s41467-026-78153-0 (PMC13720115; doi:10.1038/s41467-026-78153-0)
Supplement: Supplementary file 2 [file 41467_2026_78153_MOESM2_ESM.pdf]

nature portfolio

Corresponding author(s): Ruth Mace

Last updated by author(s): Sep 3, 2026

Reporting Summary

Nature Portfolio wishes to improve the reproducibility of the work that we publish. This form provides structure for consistency and transparency in reporting. For further information on Nature Portfolio policies, see our Editorial Policies and the Editorial Policy Checklist.

Statistics

For all statistical analyses, confirm that the following items are present in the figure legend, table legend, main text, or Methods section.

n/a	Confirmed	
☐	☒	The exact sample size (n) for each experimental group/condition, given as a discrete number and unit of measurement
☒	☐	A statement on whether measurements were taken from distinct samples or whether the same sample was measured repeatedly
☐	☒	The statistical test(s) used AND whether they are one- or two-sided<br>*Only common tests should be described solely by name; describe more complex techniques in the Methods section.*
☐	☒	A description of all covariates tested
☒	☐	A description of any assumptions or corrections, such as tests of normality and adjustment for multiple comparisons
☐	☒	A full description of the statistical parameters including central tendency (e.g. means) or other basic estimates (e.g. regression coefficient) AND variation (e.g. standard deviation) or associated estimates of uncertainty (e.g. confidence intervals)
☐	☒	For null hypothesis testing, the test statistic (e.g. F, t, r) with confidence intervals, effect sizes, degrees of freedom and P value noted<br>*Give P values as exact values whenever suitable.*
☐	☒	For Bayesian analysis, information on the choice of priors and Markov chain Monte Carlo settings
☒	☐	For hierarchical and complex designs, identification of the appropriate level for tests and full reporting of outcomes
☒	☐	Estimates of effect sizes (e.g. Cohen's d, Pearson's r), indicating how they were calculated

Our web collection on statistics for biologists contains articles on many of the points above.

Software and code

Policy information about availability of computer code

Data collection	No software was used for data collection.
Data analysis	Stan v2.35.0, RStudio v4.4.1 (packages: rethinking v2.42, ape v5.8, geiger v2.0.11, phytools v2.3.0, maps v3.4.3, tidyr v1.3.1). Code is available at Figshare: https://doi.org/10.6084/m9.figshare.30346969.

For manuscripts utilizing custom algorithms or software that are central to the research but not yet described in published literature, software must be made available to editors and reviewers. We strongly encourage code deposition in a community repository (e.g. GitHub). See the Nature Portfolio guidelines for submitting code & software for further information.

Data

Policy information about availability of data

All manuscripts must include a data availability statement. This statement should provide the following information, where applicable:

- Accession codes, unique identifiers, or web links for publicly available datasets
- A description of any restrictions on data availability
- For clinical datasets or third party data, please ensure that the statement adheres to our policy

Data are available at Figshare: https://doi.org/10.6084/m9.figshare.30346969.

Research involving human participants, their data, or biological material

Policy information about studies with human participants or human data. See also policy information about sex, gender (identity/presentation), and sexual orientation and race, ethnicity and racism.

Reporting on sex and gender	N/A
Reporting on race, ethnicity, or other socially relevant groupings	N/A
Population characteristics	N/A
Recruitment	N/A
Ethics oversight	N/A

Note that full information on the approval of the study protocol must also be provided in the manuscript.

Field-specific reporting

Please select the one below that is the best fit for your research. If you are not sure, read the appropriate sections before making your selection.

☐ Life sciences ☒ Behavioural & social sciences ☐ Ecological, evolutionary & environmental sciences

For a reference copy of the document with all sections, see nature.com/documents/nr-reporting-summary-flat.pdf

Behavioural & social sciences study design

All studies must disclose on these points even when the disclosure is negative.

Study description	Cross-cultural comparative study utilizing quantitative ethnographic data and phylogenetic multilevel modeling approach.
Research sample	The sample consists of 352 societies included in the eHRAF World Cultures database. This database was chosen because it contains primary ethnographic materials, which allowed us to code our own data about celibacy, otherwise not available in any ethnographic databases. We also utilized the D-PLACE database, which is interlinked with eHRAF and contains ready-to-use cross-cultural codes for historic and ethnographically documented human populations.
Sampling strategy	Societies were chosen based on the availability of ethnographic information. The search of the primary ethnographic materials at eHRAF was intended to maximize the number of paragraphs mentioning a keyword of interest (e.g., celibacy, marriage). No sample size-calculation method was used.
Data collection	The data were collected by performing an automated search of the primary ethnographic materials at eHRAF using pre-specified criteria and coding the variables of interest, as well as by extracting cross-cultural codes from D-PLACE. The researcher was not blind to the study hypotheses.
Timing	Data collection started in 2022 and a preliminary version of the dataset was coded in 2023. The dataset was later revised and extended for a new data. The final version of the dataset was created in 2024.
Data exclusions	Before data analysis, 19 and 7 societies, respectively, were excluded from all-societies and sex-specific celibacy samples on the basis of incomplete ethnographic records. For Eurasian subsets of these datasets, we excluded 4 societies and 1 society, respectively.
Non-participation	N/A
Randomization	N/A

Reporting for specific materials, systems and methods

We require information from authors about some types of materials, experimental systems and methods used in many studies. Here, indicate whether each material, system or method listed is relevant to your study. If you are not sure if a list item applies to your research, read the appropriate section before selecting a response.

Materials & experimental systems

n/a	Involved in the study
☒	☐ Antibodies
☒	☐ Eukaryotic cell lines
☒	☐ Palaeontology and archaeology
☒	☐ Animals and other organisms
☒	☐ Clinical data
☒	☐ Dual use research of concern
☒	☐ Plants

Methods

n/a	Involved in the study
☒	☐ ChIP-seq
☒	☐ Flow cytometry
☒	☐ MRI-based neuroimaging

Plants

Seed stocks	N/A
Novel plant genotypes	N/A
Authentication	N/A
