# Supplementary material for: Religious celibacy is linked to marriage and wealth transmission practices across different world religions
Source: Nat Commun. 2026 Sep 29;17:11230. doi: 10.1038/s41467-026-78153-0 (PMC13720115; doi:10.1038/s41467-026-78153-0)
Supplement: Supplementary file 3 [file 41467_2026_78153_MOESM3_ESM.pdf]

nature portfolio

Peer Review File

Religious celibacy is linked to marriage and wealth transmission practices across different world religions

Corresponding Author: Professor Ruth Mace

This file contains all reviewer reports in order by version, followed by all author rebuttals in order by version.

Version 0:

Reviewer comments:

Reviewer #1

(Remarks to the Author)
Overview

I really enjoyed this paper. I found it theoretically accurate and compelling and the methods appropriate. I would like to see the paper revised and published. I do, however, have several concerns that ought to be addressed before the paper is published. The concerns are outlined below, and specific commentary is also attached in a marked-up draft of the manuscript.

Here are my primary concerns:

1. I have a moderately significant concern that the article is under-cited, and especially that it relies heavily on classic papers in evolutionary anthropology without citing more recent work on related topics either from evolutionary anthropology or other relevant fields. While I agree that it is fully appropriate and necessary for the authors to cite classic papers from Goody, Dickemann, Boone, Deady et al., etc that clearly underlie the theory and analyses in the paper, there are occasionally entire sections of the text (particularly in the introduction and discussion) that only cite these texts despite the fact that many more recent papers exist for those regions or topics, sometimes from an evolutionary perspective and sometimes papers that support key points even if they come from other fields. Work is thus needed throughout the text to add to and modernize the citations.

2. In the analyses on male celibates, polygyny and polyandry are coded together vs. monogamy; this makes no sense to me theoretically, and in fact seems likely to introduce errors of interpretation. This is in contrast to the coding of polyandry and monogamy together vs. polygyny, for analyses of celibacy overall (in some cases) and for analyses of female celibates (in all cases). The latter coding is much more justifiable as—as the authors acknowledge—formal polyandry is generally associated with environments of exceptional scarcity and competition for land, making them an intensification of some of the same practices associated with monogamy, thus lumping them together is appropriate. In the case of male celibates, however, the authors argue in the methods section that polygyny and polyandry are both likely to be associated with competition among brothers—yet this is not convincing to me as the type of competition is likely to be very different. In order to address this concern, I suggest that the authors either:
a. Recode marriage systems in their analyses of male celibates and either (a) replace the current analysis with the new analysis, (b) show both analyses, or (c) show one version but clearly state the results for the other and put it in the supplement.
b. Another option—which I think would be preferable if there is statistical power for it—would be to analyze data across all three marriage types so that readers could see and compare those results. This is done for some analyses of all celibates, which I appreciated, and was disappointed to see that it was not done for the analyses of sex-specific types of celibacy.

3. The organization of the paper, with the introduction leading directly to the results and the methods at the end, makes certain aspects of the results hard to understand. This needs to be managed by telegraphing certain key aspects of the methods either at the end of the introduction or in the presentation of results. I have left several comments in the text on important topics for which this should be done.

4. The lack of inheritance data for female celibates needs to be better explained up front. While I am well aware that the

formal inheritance of property—especially land—is much more commonly patrilineal than matrilineal, and more commonly focused on sons, it is not clear why there is no attempt to analyze matrilineal inheritance or simply inheritance practices in general to show the patterns that exist. Why not contrast the societies with female celibates across matrilineal, bilateral, and patrilineal inheritance patterns? If this is related to lack of power then this should be clearly noted, but—barring a problem with power—I can't see why an analysis is not done to better clarify the patterns of association of inheritance with different types of celibacy as is done for male celibates and for celibacy overall.

5. For Figure 5 and related analyses, my interpretation of these results is somewhat different in emphasis than that of the authors. The most striking result to me is that marriage transactions from either side are very common in the context of celibacy—and much less common when there is no celibacy. It is true that there is also a relationship with marriage transactions coming from the bride's or groom's side, but it seems odd to emphasize this without emphasizing the stronger relationship.

6. The discussion could use work, as while some of it is quite clear other sections are less focused and employ numerous examples without a clear structure or point. There are also concerns about the timeframes being referred to/contrasted, lack of appropriate citations in some cases, and under-citation in other cases. Notes are included in the manuscript.

7. Finally, there are periodic instances of grammatical errors or unclear writing throughout the text—I have made suggestions for revisions in the manuscript either in a comment or in track changes. While not a significant concern for the quality of the research, these should be attended to as part of the process of revision.

(Remarks on code availability)

Reviewer #2

(Remarks to the Author)
First, we apologize to the authors for the length of this review—this was a review conducted in a collaboration between a more senior and junior scientist, so there are lots of ideas and suggestions that we hope are helpful to you. There is no inconsistency in our suggestions/concerns, but there may be some repetition (apologies again).

GENERAL
The finding that "(religious) celibacy indeed arises where marriage practices, heritable property and significant transfers of resources at marriage promote competition between individuals within families" is a valuable contribution to the scientific literature.

However, as currently presented, we don't find this adequately framed or sufficiently clearly theoretically motivated to justify acceptance by Nat Comms. The topic of how and why celibacy as a cultural trait is patterned across societies is important, and of quite general interest. But we have concerns with novelty, theoretical cogency, and (particularly) presentation that render the current MS not well-suited to Nature Communications, or at least in need of serious revision. A paper for Nature Communications should have a clear theoretical claim, and a convincing statement regarding how the present study improves/ complements/ extends previous research (i.e., what new does it bring to the field). We would encourage the authors to strongly revise their presentation for a general science journal like Nat Comms, or to send it to a discipline-specific venue.
On novelty, the authors at one juncture do acknowledge the lack of novelty to the central claim of this paper (L128-139). This should be acknowledged much earlier—the reader wants to know what the distinct contribution of the paper might be. In fact, confusingly, the first line of the current MS refers to a study showing ecological predictors of cultural traits, but there is zero ecology in this paper. The reader was expecting something very different, linking celibacy to ecological factors.

On theoretical cogency, we generally find the results convincing (if not surprising given previous work), but the logic of the causal model poorly presented. The use of DAGs is welcome and makes intuitive sense, but its set up to the paper is extremely confusing (see comments on structure/presentation). It is also surprising not to incorporate into the theoretical set up the importance of shared/unshared (divisible/indivisible and rival/non-rival) investment in the nonhuman (Lazarus and Inglis 1986, Clutton-Brock 1991), human (Ross, Borgerhoff Mulder et al. 2018), demographic (Downey 2001), and economic (Fontenay, Gobbi et al. 2024) literatures, a distinction clearly linked to all treatments of the ecology of reproduction. This might help to bridge the gap between claims (as in line 1) they are addressing ecological predictors of celibacy without a single ecological variable included! It is also surprising given the Nat Comms readership not to include any theory, evidence or modelling from studies of reproductive competition in non-humans. We do appreciate the authors have produced models and within-society analyses of the reproductive competition that might drive celibacy, but rather than simply saying "we are testing previous models", they need to lay out the logic for EACH of their predictions.

On structure we both found the whole the introductory materials (41-188, i.e., over a third of a 500-line paper) somewhat confusingly written and muddled. It consists of both ethnohistorical observations regarding celibacy and multiple anthropological studies examining the links between hypergyny, sex-specific inheritance, unigeniture, inequality, marriage type, marital payments and celibacy with the logic for inclusion, or links to celibacy, often unspecified. We do appreciate the authors want to situate their study in broader ethnographic contexts, but it would be far more effective to use this literature to justify/challenge/amend or qualify their DAG.
Below we expand on these three concerns, referring to specific points in the MS, with points listed in no particular order.

NOVELTY

I am sure a clear crisp and concise statement of novelty could be made for this paper, but I don't see one. Rather I found text that confused me with respect to what was really new in this paper. For example:

On lines 163-4 the authors write "While researchers have examined the relationship between inheritance patterns, same-sex competition, and rates of celibacy, other socio-ecological factors remain unexplored". But this is not followed up with a clear explanation of what other socioecological factors are going to be investigated, and why. In fact, because "same sex (sibling) competition" which is at the heart of the DAG can only be proxied by other variables (and these variables [marriage payments, marriage systems, and sex specific inheritance] are all analysed in earlier studies) I fail to see the novelty of these "other socio-ecological factors".

And on line 168 the authors say "but empirical studies have not investigated whether they can predict celibacy in population", after only a few lines earlier (155-162) having cited a number of studies predicting celibacy, or at least drawing logical inferences about what might be associated with celibacy.

The authors clearly state (325-328) that the finding that celibacy is most common in societies with competition over heritable wealth or marriage partners is not new. I intuit (from the ensuing text) that the novelty of their study lies in using wealth inheritance and marriage practices as proxies for resource competition (and testing these empirically). But much of the material reviewed in the introduction covers such correlational studies, so it is not clear to me what new we have actually learned. As noted above, I am sure the authors could make a more succinct and convincing claim here.

THEORY

The treatment of missing data on celibacy as absence needs prominent highlighting as a limitation earlier in the paper. The authors will be aware of recent critiques and retractions of papers based on cross cultural coding that make this mistake, and they need a very strong defence of this practice.

Regarding the theoretical framing, we think the term 'institution' is being used too generally to refer to behaviors that may not require 3rd party norm enforcement (which is how we understand institutions generally work). While the authors would certainly not be the first to contribute to this ambiguity, it weakens their theoretical explanation for the behavior of interest. In a most extreme case, if celibacy were an institution reproduced by different norm-enforcement mechanisms (e.g., punishment, conformity), you wouldn't need to invoke inclusive fitness to explain it. It's certainly possible that celibacy has collective benefits that have led to the norms promoting this behavior being culturally selected at the group level. However, this isn't something that the study argues, nor does it present evidence for such a case. The study instead argues that the distribution of celibacy across groups is due to a convergent adaptive response to ecological variation (e.g., in the form of sibling competition for parental resources), but it remains unclear why celibacy would not be more widespread within the groups that practice it, if all its members are exposed to similar "ecological drivers". Granted, the data may be limited in this regard, but a similar question can raised regarding this behavior not being more frequent across groups. For example, why is religious celibacy "rare outside Eurasia", despite same-sex competition for parental resources or mates, and despite the widespread presence of major world religions? While the study does present a partial answer to this question in the form of sex-specific celibacy, it is still applying individual or household-level explanations to account for population-level phenomena or data. Thus, the question of why some groups have religious celibates and others (presumably) don't remains unclear. We don't view this as a fatal flaw to the theory, but we think the authors' line of argumentation needs to be more clearly defended against this position.

We are also a little confused by the introduction of Trivers-Willard (lines 146-151). First the authors don't really report this correctly – albeit this is how many subsequent papers capture their hypothesis; also, Trivers and Willard were not talking about human families as implied in current text. Trivers and Willard's argument was that parents will invest in the sex with the higher returns to their investment, and that this may be males or females depending on the breeding system (Silk 1988) and the wealth/status of the parent. Critically these returns may switch across wealth levels, ultimately because of the structure of the mating system. For example, if polygyny is permissible, sons may be a better target for reproduction only among the wealthiest families. Once you recognize that TW uses individual-level logic, any predictions regarding stratification, marriage systems and inheritance (as in this paper) must include interaction effects. This was a critique that Dickemann makes of Hartung (in the cited 1982 paper). This concern in no way invalidates the analysis conducted in this paper (there is a place for population level analyses) but introducing T&W theory is probably a mistake.

Despite a relatively clear statement of expectations in the abstract, and a general set of claims that resource conflict within (or across [see ambiguity below]) same-sexed individuals might drive celibacy, there is no clear exposition of exactly how and why this might be mediated. The reader is presented with a mass of studies (see Structure and Presentation) on correlations among a suite of traits (inequality, bridewealth, dowry, primogeniture, polygyny, inheritance, etc), but no clear causal framework is developed until we see the DAG. This DAG is not explained in the text, but I found the material in the Methods section at the end much more informative than any of the earlier material (see below). Claims like (line 146) the link between wealth and primogeniture is "not a coincidence" could be dropped, if we had a clear working model from the start of the paper, and an explanation of how this model is linked to the ecological factors heralded in the first line of the paper.

There is no distinction between same-sex sibling competition and same sex competition more generally, even though the dynamics could be very different. For example, same-sex sibling competition might be expected to be sensitive to the inheritance system in a way that same-sex competition across the population might not. Similarly, the two types of

competition might play out very differently in stratified versus non-stratified societies – in the former it's feasible not all men would be in direct competition with each other if there is any economic endogamy. Again, I doubt this is a fatal flaw to the theoretical model advanced here, but it needs more thought, especially insofar as in some places the authors are clearly only thinking about sibling competition (viz line 331) and in other places they are ambivalent.

The logic of the predictions regarding polygyny escaped me. We think the authors are assuming that same sex sibling competition will be lowest among females in polygynous societies, and lowest among males in monogamous societies (which seems reasonable). But we are not sure why they are predicting celibacy to be lowest in polygynous populations and highest in polyandrous population. It is certainly not true that there is no male-male competition (over wealth or wives) in polygynous populations. Some males are clearly excluded from marriage. Even more puzzling is why the authors seem to group polygynous and polyandrous societies (Figure 3). Again, a clear statement of the theoretical logic underlying predictions for each sex would help avoid these ambiguities (see directly below).

While we strongly support the use of DAGs to represent hypotheses and analytical strategy, it would probably be clearer to have one for each hypothesis. It feels as if the authors have thrown everything into the DAG without equal attention to how each element is theoretically relevant for each sex. Here are our more specific concerns:
(i) The role of a group's phylogeny or religion (the mechanisms of these forces) is not clear, and there is no clear description of the causal model further below (L497). You expect celibacy to vary across major religions, and argue these 'cause' celibacy, but why? Earlier you said that celibacy is present in almost all major religions, but what varies is the sex that is celibate. Even if you used this DAG for that slightly more specific hypothesis (religion -> celibate sex), what does religion causally have to do with this? Is the underlying assumption that all variation in same-sex resource competition is structured along religious boundaries? This would be crucial to state, but would still raise the question why not every sibling from the competing sex who is religious becomes a monk or a nun.
(ii) There seems to be discrepancy between the DAG and the supporting text in the methods section 498-524. The text argues for 3 proxies for resource competition – marriage transactions (bridewealth and dowry, # concurrent spouses, and sex specific inheritance) but if you look at the DAG these variables are included in quite distinct roles (as both causes and consequences of their unmeasured "Same sex (sibling) competition").
(iii) I would like the authors to consider why there is no arrow in the DAG between marital composition (e.g. polygyny) and marriage transactions (e.g. bridewealth), especially given Hartung's study linking bridewealth to polygyny (where notably (for the anomaly noted above #3) the posited link doesn't go through sibling competition but population-wide competition among men).
(iv) Also questionable in the DAG is the specification that phylogeny constrains unmeasured cultural factors, but not any of the other variables like marriage systems or marriage payments.

The relative importance of religion and phylogeny (with the former generally being a stronger predictor) is an interesting finding. It's strange that this is not set up as a theoretical objective of the study. I don't believe the work was preregistered, so it would seem legitimate to treat this as a feature rather than a bug.

STRUCTURE & PRESENTATION

Regarding the abstract, although a prediction is made "Here we predict that institutional lifelong religious celibacy will arise in societies where there is sibling competition for parental resources for marriage" a specification of all 3 hypotheses, and of the precise data to be used to address these hypotheses is needed. The sentence "Monogamy, heritable wealth and significant transfers of resources at marriage have all been shown to co-evolve across cultures (Mace, 2018)" is something of a distraction at this stage.

The whole section of the history and geography of celibacy is interesting but is almost entirely devoid of any of the theoretical framing or evolutionary logic promised in the Abstract. I would suggest EITHER introduce clear hypotheses first and then frame earlier findings around hypotheses, OR rewrite closely linking the literature review to the DAG (as currently in methods). I think latter option would be best, helping to reveal the logic for the DAG, which is not explained at all in the main text. A suitable introduction would distil a few central questions from the ethnography/history/previous research, and then pose a novel theoretical claim, with a convincing statement on how the current research improves on/complements/extends previous work.
Clear statements of hypotheses must be made. In fact it is not until line 193-196 that we get a clear statement of hypotheses – this should come much earlier. A sentence like (128-130) "Scholars have suggested that constraints concerning land inheritance and partibility give rise to same-sex sibling competition and therefore celibacy (Hager 1992; Dickemann 1979; Betzig 1995) could, for example, be framed within theory (not just "scholars suggest"), and be placed at the outset of the section on sex-specific celibacy.

The Discussion is rather dense. First, there is a detailed discussion of examples (eg 367 to 392) that purport to explain some of the religion-specific variability, but it is very hard for a non-specialist in the area to assess this evidence, specifically how selectively chosen. Some more general claims about support (and failures to support) the overall model might be more digestible for advancing theory. Second by pinning claims to the simulation results in Figure 5 there seems to be quite a lot of redundancy with material covered in the Introduction. I think this could be improved by addressing concerns in #1 above. In fact Figure 5 is very hard to interpret, explained only as "When simulating the distributions of new outcomes based on the estimated parameters…". We appreciate there is material in methods (L614-626) but the rationale for these simulations needs a stronger set up in the main text. Also why is this in Discussion rather than in Results? It suggests the result is somehow not central to the story being told here. Generally, both reviewers were deeply puzzled here.

Finally, at line 428 we start getting some clearer prose that explains the results within a more comprehensive theoretical framework and points out where discrepancies occur and why. Indeed, the paragraph ending on line 477 is clearly written, and more explicitly linked to the theory that appears to underlie the paper (but, as noted above, is not clearly flagged at the outset).

SMALLER POINTS
Figure 1. What is the source of these data? Do they all refer to contemporary period or ethnographic present? How generally do authors deal with contemporaneous and non contemporaneous data.
L85-88. Logic of argument unclear. More rules less status?
L113. “elsewhere”? Really everywhere in the world not mentioned in paragraphs above? Suggest authors reword.
L187. Haddix presumably refers to (Haddix 2001).
Figure 4. Do we assume panels left to right are again transactions, marriage structures, and wealth inheritance
Results general. While I appreciate there are a lot of results to cover the presentation gets very sketchy about direction and actual findings. First, associations between variables are cryptically described as positive or negative, rather than spelling out that there is more (or less) celibacy with bridewealth, or polygyny. It’s hard for the reader to remember whether dowry is coded 1 or 2, and also hard to remember whether positive or negative is in support of hypothesis or not. As a reader you find yourself glazing over, after having turned multiple times back to what the codings were. Could you make the language more friendly? Second, (this may be relevant as text lengthens due to addressing the point above), the diverse patterning of correlations between your predictors and outcomes vary across religions (266-302) – but this doesn’t get much unpacked in the discussion; perhaps the results could go to the supplement?
L206: Since the study is not measuring how common celibacy is within a society, but rather between them, I would suggest more precise wording. For example: “Globally, the presence of lifelong celibacy is more common in societies where…”
Figure 2. All terms need description in figure legend. For example, what does wealth inheritance mean (sex biased? Primogeniture or not?), or marital composition (monogamy? polygyny? Polyandry?. Of course it becomes clear in the figures, but reader needs help through the DAG.
L215: “Overall, competition for marriage partners, or lack thereof (i.e., marriage transactions and marital compositions; Table 1), exerts...”
L218 & L230: Since you’re measuring a group-level trait, change Male celibates and Female celibates to Male celibacy and Female celibacy. Change this also in Table 1, Figure 4, and the Data-Celibacy subsection (L542-548).
L234: Figure g-h???
L238 / Table1: I really like the “% PP” column. Would it be useful to the reader to clarify that “expected side of zero” means ‘predicted directionality of effect’?
L246 / Figure 3: I liked this figure and found it clear and easy to interpret.
L257-258: “…there is about two times more variation in the probability of celibacy”. Again, I would advise “…probability of a society having at least some celibate members”, or “…probability of finding celibacy in a society”, or similar.
L260: “…religion accounts for … the probability of male celibacy being present” or “..having male (rather than female) celibates…”
L322: “ecological drivers”. You have no ecological drivers in this analysis! In fact I found “ecological conditions” throughout the discussion, which should clearly be relabelled as social or institutional (insofar as I believe you are referring to marriage systems, inheritance customs, marriage payment transactions). Even socio-ecological seems like a poor use of terminology. You are linking institutions (marriage and inheritance) to another institution (monasteries and nunneries).
L325: “…reported by several case studies in contemporary populations” (sentence is too long to string adjectives for this noun I think)
L409-11: “This is because female celibates are less common than male celibates and show a higher tendency to cluster around more closely related societies, contrary to male celibates, which are more widespread.” This is not an explanation of the result – why is the female pattern more phylogenetically constrained – but a description of the patterning of the data
L451: Sentences like “female celibates are negatively associated with transactions from the bride’s side” (Supplementary Figure 2c-3c)” are really indigestible for Discussion. Negatively associated with what transactions – again this just needs writing in clear English without assuming coding direction.
L466: replace “be” with “are”
L478-480: “Apparently maladaptive behaviours are often explained as a mismatch between ancestral and modern environments and as maladaptive by-product resulting from our tendency to conform, respectively (Boyd and Richerson 1985; Pinker 1997).” Boyd-Richerson and Pinker disagree radically on the potential sources of maladaptation. This sentence needs careful thought and rewriting.
L502-503: “Central to our causal model is the same-sex (sibling) competition for resources…”
L545-546: I’m a little confused about how you coded presence of sex-specific celibacy. How did you code societies that have both male and female celibacy? Figure 1 seems to suggest this is a separate binary outcome variable (1, presence of both male and female celibacy; 0, absence of both male and female celibacy). However, here you say you “created a binary indicator for the predominance of male celibates, e.g., monks, including societies having male celibates only as well as societies having both male and female celibates”. If you indeed lumped these two categories, why did you? If you did not, please re-phrase for clarity.
L551-554. I found no clear explanation for why polygyny is combined with polyandry as a predictor of male celibacy. All of the empirical studies reviewed point to the interaction of wealth differences and marriage rules.
L558: The term ‘substantial’ suggests a threshold has been crossed. I would prefer it if you used the terms 'relatively greater' and ‘relatively fewer’ resources (transferred at marriage) instead.

References
Clutton-Brock, T. (1991). The evolution of parental care. Princeton, Princeton University Press.
Downey, D. B. (2001). "Number of siblings and intellectual development: the resource dilution explanation." American

Psychologist 56: 497-504.
Fontenay, S., et al. (2024). Fertility in sub-Saharan Africa: The role of inheritance, ULB--Universite Libre de Bruxelles.
Haddix, K. (2001). "When polyandry falls apart: Leaving your wife and your brothers." Evolution and Human Behavior 22(1): 47-60.
Lazarus, J. and I. R. Inglis (1986). "Shared and unshared parental investment, parent-offspring conflict and brood size." Animal Behavior 34(6): 1791–1804.
Ross, C. T., et al. (2018). "Greater wealth inequality, less polygyny: rethinking the polygyny threshold model." Journal of the Royal Society Interface 15(144).
Silk, J. B. (1988). "Maternal investment in captive bonnet macaques (Macaca radiata)." The American Naturalist 132(1): 1-19.

(Remarks on code availability)

Reviewer #3

(Remarks to the Author)
I co-reviewed this manuscript with one of the reviewers who provided the listed reports. This is part of the Nature Communications initiative to facilitate training in peer review and to provide appropriate recognition for Early Career Researchers who co-review manuscripts.

(Remarks on code availability)
“I co-reviewed this manuscript with one of the reviewers who provided the listed reports. This is part of the Nature Communications initiative to facilitate training in peer review and to provide appropriate recognition for Early Career Researchers who co-review manuscripts.”

Reviewer #4

(Remarks to the Author)
The article addresses an important yet understudied topic. It is original and well done. I endorse its publication after a minor revision. I have some reservations about the analysis, but I do not believe that the results will change dramatically when methods that I would prefer are used. The only serious objection I have is to using a constant offset between polygamy (1)-monogamy (2) and monogamy (2)-polyandry (3) in the model of celibacy probability—in essence, to using simple bM*marriage in the model formula (more info below).

Petr Tureček

More important points:

Pages 3–4: The construction “Where rules of female Buddhist monastic ordination can be found [..] they significantly outnumber the corresponding male collection of rules, signifying the unequal status of male and female celibates […] while [elsewhere] nuns are more common and respected” suggests (although only “unequal” is stated explicitly) that lower status is linked to more rules. This is possible, but in the Hindu tradition, for instance, higher castes typically need to observe more rules. Similar trends can also be found in Judaism, where the ability to follow religious rules more strictly is connected to higher social status and prestige. Can the authors elaborate on this? If they simply want to point out that a different number of regulations suggests a difference in status, that is fine, but they should then clarify why they do not want to make any claims about the direction of the inequality. However, given their expertise, I believe they are capable of making an informed claim about who holds lower status in this case and why is difference in status reflected in Buddhist monastic tradition in this particular way.

Page 4: The authors note that “there was no male equivalent [of calibate ‘aclla’ women] among the Inca.” This is especially interesting given Inca are famous for great reproductive inequality among men. Ruling class Inca’s – with Sapa Inca on top – were entitled to marry many more wives than commoners. Can authors, perhaps, return explicitly to the Inca case in the discussion? Why did not they develop male celibate if the competition between males was so big? Is it because they did not practice monetary transactions at marriage?

Page 6 and elsewhere: When the authors mention the Trivers-Willard hypothesis, could they take a moment to discuss why the effect is not sufficient in humans to compensate for hypergamy? Do high-status women really have more male children (excluding and including selective abortions and infanticide)? If not, why not? How solid is the Trivers-Willard effect among other animals?

Page 6, bottom: Why does monogamy lead to asymmetry in competition for marriage partners (less strict for men, more strict for women)? Is it because it is easier to establish a cultural norm that allocates rival wealth to one of the brothers deterministically (e.g., primogeniture), while all women, in effect, compete for these firstborn men?

Page 7: In the DAG, I find the absence of subsistence strategy somewhat surprising. Is it omitted because no causal path would require adding, for instance, a contrast between intensive agriculture and other subsistence strategies in the model? The paper mentions causal paths from agriculture/pastoralism to cultural norms in several places, so its omission here seems odd. Otherwise, it should be made explicit that this DAG is more a part of the analytical strategy than a claim of

exclusive causality between data variables. For instance, wealth inheritance can definitely impact social stratification. The causality is not unidirectional. (But a model with cycles cannot be expressed as a DAG.)

Figure 5: The effects on this scale seem rather small. What do you think is the reason for this, given that the hypothesis is so sound and the presented anecdotes so convincing? It would be helpful to add a paragraph about this in the discussion.

Page 10: The authors state that the effect of marriage transactions is positive in Christian (90% PP) and Buddhist (55% PP) societies, while it is negative in all others. Perhaps the authors should acknowledge that these effects form a continuum, with the Buddhist slope being right on the edge. A 49% PP would be interpreted as "negative," correct? This suggests that Christian societies may be the exception in this respect.

Page 15: Overall, the most salient result is that the more competition between relatives, the more celibacy can be expected in the population, because "societies that have both are most frequent in [your] sample." Do you share this opinion? If so, could you elaborate on it in the discussion?

Page 15: The authors state that "…it is adaptive for parents to allocate resources to a smaller number of offspring…" While this may sometimes be true, primogeniture can still evolve when firstborns can assert their interests—since it is always more favorable to them than to their parents, as they are twice as related to their own children as any other relatives are. Many narratives around primogeniture emphasize parental agency while overlooking the role of direct sibling competition in shaping societal norms. In fact, parents are incentivized not to split resources among offspring only if reproductive returns increase with the amount of rival resources that must be divided. This can occur, for example, when agricultural success depends on a relatively small amount of arable land, but many other circumstances can produce similar resource-reproduction relationships. A reflection on these issues would be appreciated. See also Ross, C. T., Borgerhoff Mulder, M., Oh, S. Y., Bowles, S., Beheim, B., Bunce, J., ... & Ziker, J. (2018). Greater wealth inequality, less polygyny: Rethinking the polygyny threshold model. Journal of the Royal Society Interface, 15(144), 20180035, for a related discussion on rival resources, polygyny, and environmental carrying capacity.

Page 16: The authors write that the depletion of fitness can be achieved "…by sending away some offspring to become celibate monks or nuns." It would be interesting to address how far celibate offspring are typically sent, as findings on celibate monks increasing parental inclusive fitness suggest that celibate siblings become net contributors to family resources. To do so, they would need to live in reasonable proximity. On the other hand, if primogeniture leads to the large-scale dispersal of later-borns (such as when younger sons are sent to the military), their direct reproduction does not necessarily have to be restricted—they may contribute to parental inclusive fitness through direct reproduction rather than by supporting nephews and nieces. All in all, celibacy seems to function as an alternative to actual dispersal. Do the authors agree? If so (or even if not), could they reflect on this in the article? Otherwise, this issue will likely keep resurfacing.

Page 16: The authors note that "However, while low-status males were usually sent to beggar castes and, more rarely, to monasteries, e.g., in Tamil India, male monastic institutions are predominantly found in areas with the highest rates of groom-wealth (Slater 1918), females are not associated with any form of religious celibacy." This contradicts the predictions of the authors' hypothesis, which is fine (as counterexamples are expected given that the distribution of outcomes follows the patterns in Figure 5). Nevertheless, this raises an interesting question: what do the extra unmarried women in this society do? Could the authors elaborate?

Supplement, Page 19: What are the authors' thoughts on the existence of societies where assigning children to celibate social roles does not prevent them from inheriting (and thus potentially depleting) portions of family wealth? This is particularly relevant given the interesting example where "sons and daughters who become monks or nuns receive their share of the property at the time they take monastic vows…"

Methods:

The description of the statistical analysis in its current form does not clearly convey what was precisely done. I had to examine the model code to understand the details. Sharing the models in the form of equations—perhaps in the paper's supplement—would be much appreciated so that other readers do not have to search through the code. Precisely describing the models in the methods section is also necessary. Regarding what was done, I have several comments:

1. The marital composition is described as a "3-level ordinal scale" in the models of celibacy. However, from the code, it appears to be a variable with values 1, 2, or 3 that is simply multiplied by a slope bM. This approach imposes equal spacing (on the log-odds scale) between polygyny and monogamy, as well as between monogamy and polyandry. In my opinion, this is not the correct way to incorporate an ordered-categorical predictor into a logistic regression.
The authors should instead capture the effect with two parameters: one representing the total difference between the lowest and highest competition categories (polygyny and polyandry) and another determining the relative position of monogamy between them, which could be closer to either polygyny or polyandry. (See, for instance, how education is incorporated in Model 12.6 of Statistical Rethinking, 2nd edition, which the authors reference.)
Alternatively, they could use the approach employed in their models where celibacy presence is investigated by sex, using two binary indicators. This would allow the polyandry level to emerge as the sum of two (likely positive) parameters, naturally yielding the highest celibacy baseline.
Which of these two models is preferable should be treated as an empirical question, potentially decided by predictive out-of-sample fit measures such as WAIC.

2. This “3-level ordinal scale” variable is also not standardized or transformed in any way, meaning that the intercept of the model now corresponds to the nonexistent value 0 on this scale. Am I correct? This seems unfortunate. (The extremely wide compatibility interval for celibacy probability in polyandrous societies in Figure 3B may also be a result of this choice.)

3. The analysis description should explicitly state that varying intercepts and slopes are used per religion and that uncorrelated varying effects are assumed. The current version of the manuscript only mentions “varying effects” in the “Analysis” section, which is too vague.
I would also consider modeling intercorrelated intercepts and slopes per religion, as, for instance, a religion with a high baseline probability of “monks” cannot be expected to show such large effects of marital transactions on this probability. However, given the number of categories in the religion variable, the precision of estimates may not improve drastically, and the compatibility interval for the correlation itself would likely be very wide. It is up to the authors to decide whether this refinement is worth their time.

4. The authors claim to use “weakly informative” priors, yet all the priors are unbiased (centered around 0). Do you mean “weakly regularizing”? Or am I misunderstanding the terminology? Weakly informative typically implies a prior that nudges estimates toward a particular direction, such as a positive or negative slope, or toward a smaller or larger intercept than 0.5.

5. Treating "indigenous" as an equivalent category to Buddhism or Christianity can be misleading. For instance, the current models assume potentially constant variance within Buddhist and within “none of the above” societies. Is this intentional? Is there a way to address it, such as adding an additional dispersion parameter for the “indigenous” category? While I believe this would not change the main results (which are primarily drawn from Christian and Buddhist societies), acknowledging this limitation in the models would be helpful.

6. I have reservations about how phylogeny is incorporated into the models. Currently, the distance matrix is simply multiplied by an (estimated) number. This effectively implements a Brownian motion model, which does not distinguish between the similarity of closely related cultures and the rate at which similarity decays with linguistic distance. Is there a specific reason for using this model (beyond the fact that the single sigma can be conveniently plotted against the standard deviations of by-religion intercepts and slopes in Figure 4)?
Have the authors considered using an Ornstein-Uhlenbeck (exponential, cov_GPL1 in rethinking) kernel (this would be probably my preference) or a quadratic (cov_GPL2) alternative? These approaches would allow for a better estimation of the effect of relatedness between observations.

7. In the analysis, the authors state that they used “software available at the ASJP website to calculate a pairwise distance matrix between languages” and then constructed a tree, while the model essentially uses an inverse of a distance matrix. Doesn’t forcing languages into a tree structure—when their relationships may resemble a network rather than a series of bifurcations—result in information loss? Would it not be better to omit this unnecessary step and use the original language similarity matrix directly?

Technical details:

Page 4: The sentence “However, while in Tibetan and Theravāda Buddhism, the main form of Buddhism in Sri Lanka, Myanmar, Laos, Cambodia, and Thailand, nuns are rare compared to monks, in Vietnam, China, Korea, and Japan, nuns are more common and respected (Kieschnick 2007; Powers 2007; Micheletti et al. 2022).” is long and not easy to parse.
A bracket would improve readability. How about:
"However, while in Tibetan and Theravāda Buddhism (the main form of Buddhism in Sri Lanka, Myanmar, Laos, Cambodia, and Thailand), nuns are rare compared to monks, in Vietnam, China, Korea, and Japan, nuns are more common and respected (Kieschnick 2007; Powers 2007; Micheletti et al. 2022)."

Page 6: I do not understand the sentence: 'For example, a farm cannot be divided Haddix.' Is this a typo or an incomplete citation?

Page 6-7 and elsewhere: the authors should more clearly define the difference between social stratification and wealth inequality. Throughout the manuscript, these terms seem to be used interchangeably, though they are not the same (as some usages in the manuscript also suggest). Wouldn’t it make sense to include both in the DAG in Figure 2?

Page 7: I suggest moving the DAG closer to the methods section. As it stands, it feels somewhat disconnected, leaving the reader zigzagging between the methods and the figure when reading about the models.

Figure 3: These figures seem ugly to me. Why are the posterior mean circles so large if their size has no meaning? Adding a grid behind each panel would be helpful. The 'PP xx.xx %' text is also too big and sometimes overlaps the bars.

Page 20: I advise rehosting the code and data on a platform other than GitHub. While GitHub is great for version control, the final product should be shared on a platform that allows generating permanent links (DOIs).

(Remarks on code availability)
The code is understandable. It contains a README file (although typesetted in a very large font - like if everything is a headline, why?). As a suggest above, please rehost to a platform that allows generating DOI for the files.

Version 1:

Reviewer comments:

Reviewer #1

(Remarks to the Author)
I am reviewing this paper for the second time after the authors completed major revisions. This paper, always interesting, is much improved overall—and I now think that it can be accepted pending minor revisions. Specifically, the introduction is much clearer and more streamlined, major concerns raised by reviewers in terms of the analysis have been addressed, and the discussion is now organized, well-written, and robust.

Below I include a list of mostly minor, though a few more significant, comments that I suggest be addressed prior to publication. Among these, the only significant concern is with regard to acknowledgment that the analysis is not a formal causal analysis, and tweaking some of the language in acknowledgement of this.

p. 2
The authors write that "but this is more than outweighed by an indirect fitness benefit obtained by increasing the competitiveness of their other offspring."
This statement needs to be qualified. I suggest "is theoretically more than outweighed…" or "potentially more than outweighed….".

The authors write that "This, in turn, enhances the reproductive fitness of the monk's brothers,"
Again, this needs to be qualified. I suggest "This, in turn, IS EXEPECTED TO enhance the reproductive fitness of the monk's brothers OR THE NUN'S SISTERS,"

The authors write "generated by marriage patterns". I recommend "generated IN PART by marriage patterns".

Define "rival" in paragraph 2.

The authors write "a lack of marriage opportunities in daughters"
This should be "among daughters".

The authors write that "In societies where marriage customarily involves wealth transfers from the bride's side of the family to the groom's (groom-wealth 35) or to the couple or the bride ('dowry'36)"
While I fully agree that this is a meaningful distinction theoretically, this distinction is not commonly made in the literature or among people in such cultures. It's also common that dowries may serve both as groom wealth and endowment in the same societies (e.g. most of modern South Asia). Given that the authors only use one variable—dowry—to cover these kinds of transactions in their analysis, it does not make sense to me to set this up to read as if they will separate groom wealth and dowry here.

p. 3
The authors write that "While classical studies of celibacy relied on case studies and were largely anecdotal or correlational, this work is novel in providing a systematic and cross-cultural test of these associations."
The work in this paper is also correlational (though a more robust version) so I am not sure I would emphasize this critique of other work.

The authors write that "With respect to marital composition, we expect that celibacy is more likely to occur in societies where that number is equal"
The phrase "that number is equal" is not clear; I suggest updated wording.

The authors write that "However, given that matriliny is significantly less common than patriliny and inheritance in matrilineal societies is commonly passed down to nephews (i.e., diagonally), we do not expect matrilineal inheritance to play a role in driving female celibacy and thus leave this prediction out from testing."
Is this left out due to low expectations, or because it is very rare so you just don't have data to test it? Given that the response to reviewers implies the latter, I suggest just saying that it's because there aren't enough cases to test this.

p. 7 Results
The authors write that "globally, marriage transactions—either from the groom's side, bride's side or both sides—increased the likelihood of lifelong celibacy but the effect was weak"
This causal-sounding language is not appropriate as the analyses presented (while appropriate) are not causal. It would be clearer to say that marriage transactions are "associated with an increased likelihood of celibacy."

p. 9
In Figure 3, panels a and c should be shifted to a 0-1 scale on the y axis as is already true for panels d-h. Though I realize you are trying to have them match with panel b, it is very difficult to see the effect on the 0-2 scale.

p. 10
The authors write that "We found that Buddhism and, to a similar but lesser degree, Christianity exhibited the strongest

support for our predictions, in terms of both magnitude and directionality of effects."
"To a similar but lesser degree" is not clear; saying it this way makes it sound like the magnitudes are similar. I suggest "Buddhism and, similarly but to a lesser degree, Christianity…"

p. 11
The authors write:
"male celibacy was mostly driven by marriage transactions and marital composition,"
"female celibacy was driven mostly by marriage transactions though it was generally less prominent."
Again, I suggest altering causal-sounding language.

p.15, Discussion
See concerns about causal language below, and take these into account when editing the discussion. Generally, though, this section is really strong.

p. 17
The authors write "while the results proved to serve as a baseline for subsequent sex specific analyses."
What does 'proved' mean here? I suggest rewording to clarify.

The authors write that "Lifelong religious celibacy, an apparently maladaptive behavior, is often explained as the result of an evolutionary mismatch or as a by-product of adaptation."
These alternative explanations have not been discussed in the paper, even in the introduction where they are only reviewed in a single sentence at the very beginning of the paper. It is thus odd to mention it here. I suggest citing and clarifying these perspectives in the introduction before referring to them here.

The authors write "and causal inference methods."
Again, the analyses presented are not causal. I would change this language.

p. 18 Methods

Regarding the "Causal model" section and related issues:
The use of a DAG does not mean that the analyses produce causal inferences. While a DAG hypothesizes a causal model, no testing here is being done that would actually allow causality to be ascertained. I would thus steer away from causal language as many readers will find it inappropriate even if they find your methods are appropriate.

Related to this, while I agree that the DAG hypothesized is appropriate for this paper and analysis, it hypothesizes a particular set of relationships among variables—but this is not the only way that the variables could potentially be related. For example, it is far more likely that marriage transactions and patterns of wealth inheritance are co-evolved with celibacy under conditions of competition for limited resources than that one actually 'causes' the other.

In light of both of these concerns, I recommend the addition of a few sentences to the methods section or the SI directly addressing these concerns about causality.

The authors write that "Firstly, we assumed that marriage transactions are a direct cause of resource competition" is very appropriately worded with the use of "assumed".
I suggest that similar terms are used in the following points:
• "Secondly, marital composition, defined as a socially recognized number of concurrent spouses for a given sex, i.e., polygyny, monogamy or polyandry, IS ASSUMED TO [OR, ALTERNATIVELY, IS HYPOTHESIZED TO] influence resource competition indirectly through marriage transactions"
• "Thirdly, patterns of wealth inheritance are ASSUMED TO BE another cause of resource competition"

The authors write "involve relatively fewer amounts of resources"
This should be "involve fewer resources" or "involve smaller amounts of resources"

The authors write "take other form"
Should be "take another form"

P. 20
The authors write "was used as proxy for wealth inequality"
This should be "as a proxy for wealth inequality" as the verb 'was' was already used at the beginning of the sentence.

I was also asked by the editorial team to comment specifically as to whether the revision has been responsive to Reviewer 2, who was no longer available. I also evaluated their responses to Reviewer 4.

After carefully reviewing the comments made by reviewers 2 and 3 (in a joint review) and 4, I still think that this manuscript can be accepted pending minor revisions—or given a decision of minor revisions if that is the norm at Nature Communications. Generally speaking, the main concerns of the other reviewers have been thoughtfully addressed, either in terms of changes to the text of the manuscript or in terms of appropriate responses to the comments in the rebuttal. It should

be noted especially that this manuscript was heavily revised (major revisions), including significant rewriting of the introduction and discussion, in response to the first round of review, thus not all previous comments are relevant to the current manuscript.

Below I outline a few minor issues which one could argue were not fully addressed by the authors, but none of these seems particularly serious to me—thus the recommendation for minor revisions.

Reviewers 2 and 3
The authors do not address the issue of including the inclusion of ecology in the text, but they have dealt with this comment and responded in the rebuttal.

R2/3 original comment: The treatment of missing data on celibacy as absence needs prominent highlighting as a limitation earlier in the paper. The authors will be aware of recent critiques and retractions of papers based on cross cultural coding that make this mistake, and they need a very strong defence of this practice.

Author response: We are aware of the case of mistreatment of missing data and have provided a paragraph discussing this limitation in the Discussion.

My comment: The authors have responded to this, but not in detail. I am fine with what they have done, but perhaps the other reviewers would want something more direct. Perhaps a slight enhancement of the current response.

There is a concern about group level data and individual-level explanatory models. I think the authors have addressed this appropriately, and in fact I don't share the reviewers' concerns on this front since there is no reason to expect the two will be different in this case and no way to realistically use a different sort of data for this analysis.

R2/3 original comment: Figure 1. What is the source of these data? Do they all refer to contemporary period or ethnographic present? How generally do authors deal with contemporaneous and non contemporaneous data.

Author response: The data used for the figure is from eHRAF online database. These are our own data that we used in our analysis. We have added this information in the figure caption. The data refers largely to ethnographic present. Despite the bias due to ethnographic present is a persistent issue in cross-cultural research, our own work and other studies (see 'Ringen, E., Duda, P., & Jaeggi, A. V. (2019). The evolution of daily food sharing: A Bayesian phylogenetic analysis. Evolution and Human Behavior, 40(4), 375-384') have shown that it is a relatively minor source of variation. Phylogenetic relatedness is much more important. Therefore, on these grounds, we did not account for the effect of ethnographic present in this study. Note also that we already used two sources of variation – phylogeny and religion. Yet another source of variation could result in overparameterization of our models, particularly in sex-specific celibacy with quite small sample sizes.

My comment: The point about the ethnographic present should be included in the manuscript (I do not see it there) or at least in the supplement.

Reviewer 4

R4 original comment: 2. This "3-level ordinal scale" variable is also not standardized or transformed in any way, meaning that the intercept of the model now corresponds to the nonexistent value 0 on this scale. Am I correct? This seems unfortunate. (The extremely wide compatibility interval for celibacy probability in polyandrous societies in Figure 3B may also be a result of this choice.)

Author response: While you are probably right, we hope that our additional analysis - modeling marital composition as ordinal - offsets this. It shows that polyandry increases celibacy, particularly in the Eurasian subsample (see Results and Supplementary Figures 1 and 4).

My comment: This has not been dealt with directly in the text, but it does not seem like a concern to me in the current version of the manuscript.

There are numerous statistical topics raised by R4. Generally speaking, the authors have either addressed these (~65% of the time) or explained their reasons for not doing so (~35%). Most of this I find thoughtful and reasonable, though there are a few comments that I am not enough of an expert to evaluate. I am not concerned with this paper moving forward, but a statistical specialist should be asked if there is concern from the editors.

(Remarks on code availability)

Reviewer #4

(Remarks to the Author)
I see most of my concerns adequately addressed in the discussion and supplement.

What I do not understand is, however, why the central analysis (if I read the current version correctly) remained the one that treats variable “marriage” as continuous (values 1-3, implying also intercept being estimated for a contrafactual 0 value). Clearly, polyandry is not “twice as far” from polygyny than monogamy as the model with categorical predictor suggests. I find Figure 3 (b) presented as the main result misleading given the findings from ordinal model (that, however, cannot capture the possibility of celibacy probability decreasing again from monogamy to polyandry!) and the categorical model. I would strongly recommend making the categorical model the main one and omit Fig. 3b altogether or place it in the supplement (preferably after changing the continuous variable to -1 – 0 – 1, where 0 is monogamy… But still. Even such a model would be misleading and clearly dominated by the categorical model. Am I not right? What would the model comparison using WAIC, PSIS or similar suggest?)

Otherwise, when zigzagging between the main text and supplement, I discovered a typo:
“Similarly, in Hindu societies, male celibacy aligns with our predictions (Supplementary Figures 2b–7b)” Supplementary Figure 2 is now the single-panel categorical model plot without.

(Remarks on code availability)
Data and code are available and allow to reproduce the results.

Version 2:

Reviewer comments:

Reviewer #1

(Remarks to the Author)
I have reviewed this article twice before, and the authors have now addressed all of my comments. I recommend that the paper be accepted and published.

(Remarks on code availability)

Reviewer #4

(Remarks to the Author)
I am pleased to see that the manuscript has been revised according to my suggestions.

I would, however, now strongly suggest to plot a different y limit in panels a-c in Figure 3. All the "action" is happenning below 0.2 or so and the reader can now see almost no difference between the groups as the y-limit reaches all the way to 1. If authors worry that readers might be confused about different y-limits across panels, they can highlight the difference (and the fact that the models can still consider the full 0-1 probability scale) in the figure's caption.

I have no further comments. Congratulations on a fine paper!

(Remarks on code availability)
The code is fine.

Open Access This Peer Review File is licensed under a Creative Commons Attribution 4.0 International License, which permits use, sharing, adaptation, distribution and reproduction in any medium or format, as long as you give appropriate credit to the original author(s) and the source, provide a link to the Creative Commons license, and indicate if changes were made.
In cases where reviewers are anonymous, credit should be given to 'Anonymous Referee' and the source.
The images or other third party material in this Peer Review File are included in the article's Creative Commons license, unless indicated otherwise in a credit line to the material. If material is not included in the article's Creative Commons license and your intended use is not permitted by statutory regulation or exceeds the permitted use, you will need to obtain permission directly from the copyright holder.
To view a copy of this license, visit https://creativecommons.org/licenses/by/4.0/

Reviewer #1 (Remarks to the Author):

Overview

I really enjoyed this paper. I found it theoretically accurate and compelling and the methods appropriate. I would like to see the paper revised and published. I do, however, have several concerns that ought to be addressed before the paper is published. The concerns are outlined below, and specific commentary is also attached in a marked-up draft of the manuscript.

Thank you for your appreciation of our work. We have provided feedback both to the comments below and in the attached draft. Note that because of the substantial revision of the manuscript, some of your comments may no longer be relevant. In such cases, we let notified the reviewer.

Here are my primary concerns:

1. I have a moderately significant concern that the article is under-cited, and especially that it relies heavily on classic papers in evolutionary anthropology without citing more recent work on related topics either from evolutionary anthropology or other relevant fields. While I agree that it is fully appropriate and necessary for the authors to cite classic papers from Goody, Dickemann, Boone, Deady et al., etc that clearly underlie the theory and analyses in the paper, there are occasionally entire sections of the text (particularly in the introduction and discussion) that only cite these texts despite the fact that many more recent papers exist for those regions or topics, sometimes from an evolutionary perspective and sometimes papers that support key points even if they come from other fields. Work is thus needed throughout the text to add to and modernize the citations.

Thank you for highlighting this. We have cited more up-to-date relevant work but please note that the manuscript has undergone substantial revisions and some sections to which the comment may apply have been removed entirely. Also, we included citations suggested by other reviewers, while at the same time adhering to the citation limit (max 70 citations). If the reviewer has a specific work in mind that we have missed, we'll be happy to include it if the citation is provided (if space permits).

2. In the analyses on male celibates, polygyny and polyandry are coded together vs. monogamy; this makes no sense to me theoretically, and in fact seems likely to introduce errors of interpretation. This is in contrast to the coding of polyandry and monogamy

together vs. polygyny, for analyses of celibacy overall (in some cases) and for analyses of female celibates (in all cases). The latter coding is much more justifiable as—as the authors acknowledge—formal polyandry is generally associated with environments of exceptional scarcity and competition for land, making them an intensification of some of the same practices associated with monogamy, thus lumping them together is appropriate. In the case of male celibates, however, the authors argue in the methods section that polygyny and polyandry are both likely to be associated with competition among brothers—yet this is not convincing to me as the type of competition is likely to be very different. In order to address this concern, I suggest that the authors either:

a. Recode marriage systems in their analyses of male celibates and either (a) replace the current analysis with the new analysis, (b) show both analyses, or (c) show one version but clearly state the results for the other and put it in the supplement.

b. Another option—which I think would be preferable if there is statistical power for it—would be to analyze data across all three marriage types so that readers could see and compare those results. This is done for some analyses of all celibates, which I appreciated, and was disappointed to see that it was not done for the analyses of sex-specific types of celibacy.

Thank you for bringing this up. We acknowledge that coding of marital composition in case of male celibacy was not completely appropriate and that it is probably more relevant for females. Therefore, we followed both suggestions and a) recoded marital composition to 0-monogamy/polyandry vs. 1-polygyny in models for male celibacy; and b) we also added an analysis of both celibacy and male celibates where we treated marital composition as both categorical and ordinal predictor, thus estimating the parameter for each category. See the Results section for details.

3. The organization of the paper, with the introduction leading directly to the results and the methods at the end, makes certain aspects of the results hard to understand. This needs to be managed by telegraphing certain key aspects of the methods either at the end of the introduction or in the presentation of results. I have left several comments in the text on important topics for which this should be done.

Thank you, we have reorganized the paper as suggested by your comments in the manuscript. Both Introduction and Results now provide information about the methods so the readers will have sufficient understanding of what was done before the actual results.

4. The lack of inheritance data for female celibates needs to be better explained up front. While I am well aware that the formal inheritance of property—especially land—is much more commonly patrilineal than matrilineal, and more commonly focused on sons, it is not

clear why there is no attempt to analyze matrilineal inheritance or simply inheritance practices in general to show the patterns that exist. Why not contrast the societies with female celibates across matrilineal, bilateral, and patrilineal inheritance patterns? If this is related to lack of power then this should be clearly noted, but—barring a problem with power—I can't see why an analysis is not done to better clarify the patterns of association of inheritance with different types of celibacy as is done for male celibates and for celibacy overall.

Thank you for raising this point. In our analysis of celibacy overall, the coding included matrilineal inheritance. We did not analyze this effect in case of female celibacy for two main reasons:

1) there are only two matrilineal societies in our sample that have celibacy: Bemba having female celibacy, and Kerala, having both male and female celibacy

2) the coding of matrilineal inheritance in the variable we used for analyses is not appropriate for the analysis of female celibacy: one level essentially codes for diagonal inheritance, i.e., by nephews, while the other refers to matrilineal 'heirs', which may not necessarily include only daughters.

We provide justification for this in the Introduction.

5. For Figure 5 and related analyses, my interpretation of these results is somewhat different in emphasis than that of the authors. The most striking result to me is that marriage transactions from either side are very common in the context of celibacy—and much less common when there is no celibacy. It is true that there is also a relationship with marriage transactions coming from the bride's or groom's side, but it seems odd to emphasize this without emphasizing the stronger relationship.

Since we have reanalyzed the models for marriage transactions due to changes in our coding as well as in our DAG, as suggested by other reviewers. We also back our interpretations by statistical evaluation of these distributions so the interpretations of these models might have changed. See Figures 4 and 5 in the main text and Figures 12-15 in the Supplementary Materials.

6. The discussion could use work, as while some of it is quite clear other sections are less focused and employ numerous examples without a clear structure or point. There are also concerns about the timeframes being referred to/contrasted, lack of appropriate citations in some cases, and under-citation in other cases. Notes are included in the manuscript.

Following your advice, the discussion has been substantially revised. We believe that it is now more balanced and focused.

7. Finally, there are periodic instances of grammatical errors or unclear writing throughout the text—I have made suggestions for revisions in the manuscript either in a comment or in track changes. While not a significant concern for the quality of the research, these should be attended to as part of the process of revision.

Thank you, we have accepted the changes and responded to the comments in the manuscript.

Reviewers #2 and #3 (Remarks to the Author):

First, we apologize to the authors for the length of this review—this was a review conducted in a collaboration between a more senior and junior scientist, so there are lots of ideas and suggestions that we hope are helpful to you. There is no inconsistency in our suggestions/concerns, but there may be some repetition (apologies again).

Thank you for your extensive yet insightful review. We have implemented most of your advice but given the advice from other reviewers, the manuscript has undergone substantial revision, thus some of your comments may no longer be relevant. In such cases, we notify the reviewers. Please see our responses below.

GENERAL
The finding that "(religious) celibacy indeed arises where marriage practices, heritable property and significant transfers of resources at marriage promote competition between individuals within families" is a valuable contribution to the scientific literature. However, as currently presented, we don't find this adequately framed or sufficiently clearly theoretically motivated to justify acceptance by Nat Comms. The topic of how and why celibacy as a cultural trait is patterned across societies is important, and of quite general interest. But we have concerns with novelty, theoretical cogency, and (particularly) presentation that render the current MS not well-suited to Nature Communications, or at least in need of serious revision. A paper for Nature Communications should have a clear theoretical claim, and a convincing statement regarding how the present study improves/ complements/ extends previous research (i.e., what new does it bring to the field). We would encourage the authors to strongly revise their presentation for a general science journal like Nat Comms, or to send it to a discipline-specific venue.

Thank you for raising this. We agree that the original presentation and theoretical framing lacked clarity, including how the present study improves previous knowledge. Following this, we have restructured the Introduction with the focus for a more general audience.

On novelty, the authors at one juncture do acknowledge the lack of novelty to the central claim of this paper (L128-139). This should be acknowledged much earlier—the reader wants to know what the distinct contribution of the paper might be. In fact, confusingly, the first line of the current MS refers to a study showing ecological predictors of cultural traits, but there is zero ecology in this paper. The reader was expecting something very different, linking celibacy to ecological factors.

Following this and other advice, we have completely rewritten the Introduction which we hope now more clearly acknowledges what is novel and what makes the paper distinct from previous studies. On this note, the Introduction now no longer mentions ecology (but see our explanation of using the term in the next comment).

On theoretical cogency, we generally find the results convincing (if not surprising given previous work), but the logic of the causal model poorly presented. The use of DAGs is welcome and makes intuitive sense, but its set up to the paper is extremely confusing (see comments on structure/presentation). It is also surprising not to incorporate into the theoretical set up the importance of shared/unshared (divisible/indivisible and rival/non-rival) investment in the nonhuman (Lazarus and Inglis 1986, Clutton-Brock 1991), human (Ross, Borgerhoff Mulder et al. 2018), demographic (Downey 2001), and economic (Fontenay, Gobbi et al. 2024) literatures, a distinction clearly linked to all treatments of the ecology of reproduction. This might help to bridge the gap between claims (as in line 1) they are addressing ecological predictors of celibacy without a single ecological variable included! It is also surprising given the Nat Comms readership not to include any theory, evidence or modelling from studies of reproductive competition in non-humans. We do appreciate the authors have produced models and within-society analyses of the reproductive competition that might drive celibacy, but rather than simply saying “we are testing previous models”, they need to lay out the logic for EACH of their predictions.

Following your comment, the revised Introduction provides a broader theoretical framing of our predictions, including the discussion of reproductive competition in both human and non-human literature. Each prediction is now laid out in the Introduction and framed within the logic of the DAG. Also, we are grateful for the references provided by the reviewers which have been implemented in the text.

To your point about using the term “ecology”/”ecological” – from our perspective of human behavioral ecologists, culture is an integral part of human ecology. Culture constitutes a

major part of the environment humans live in. We use culture to adapt to our "ecological" environment but also to our cultural environment. It is really difficult to draw a clear line between "ecology" and "culture". We are all familiar with the futility of such dichotomizing approaches in the past.

For more discussion on this topic, we'd like to refer the reviewers to these publications:

Mace, R. (2014). Human behavioral ecology and its evil twin. *Behavioral Ecology*, *25*(3), 443-449.

Micheletti, A., Brandl, E., & Mace, R. (2025). What is cultural evolution anyway?. In *Evolutionary Social Sciences: A Tour* (pp. 141-149). Wiesbaden: Springer Fachmedien Wiesbaden.

On structure we both found the whole the introductory materials (41-188, i.e., over a third of a 500-line paper) somewhat confusingly written and muddled. It consists of both ethnohistorical observations regarding celibacy and multiple anthropological studies examining the links between hypergyny, sex-specific inheritance, unigeniture, inequality, marriage type, marital payments and celibacy with the logic for inclusion, or links to celibacy, often unspecified. We do appreciate the authors want to situate their study in broader ethnographic contexts, but it would be far more effective to use this literature to justify/challenge/amend or qualify their DAG.
Below we expand on these three concerns, referring to specific points in the MS, with points listed in no particular order.

Thank you for this. Following your advice, the revised Introduction now contains less ethnographic examples and those that we cite are used mostly to lay the ground for justifying the logic of our DAG.

NOVELTY
I am sure a clear crisp and concise statement of novelty could be made for this paper, but I don't see one. Rather I found text that confused me with respect to what was really new in this paper. For example:
On lines 163-4 the authors write "While researchers have examined the relationship between inheritance patterns, same-sex competition, and rates of celibacy, other socio-ecological factors remain unexplored". But this is not followed up with a clear explanation of what other socioecological factors are going to be investigated, and why. In fact, because "same sex (sibling) competition" which is at the heart of the DAG can only be proxied by other variables (and these variables [marriage payments, marriage systems, and sex specific inheritance] are all analysed in earlier studies) I fail to see the novelty of these

“other socio-ecological factors”. And on line 168 the authors say “but empirical studies have not investigated whether they can predict celibacy in population”, after only a few lines earlier (155-162) having cited a number of studies predicting celibacy, or at least drawing logical inferences about what might be associated with celibacy. The authors clearly state (325-328) that the finding that celibacy is most common in societies with competition over heritable wealth or marriage partners is not new. I intuit (from the ensuing text) that the novelty of their study lies in using wealth inheritance and marriage practices as proxies for resource competition (and testing these empirically). But much of the material reviewed in the introduction covers such correlational studies, so it is not clear to me what new we have actually learned. As noted above, I am sure the authors could make a more succinct and convincing claim here.

The novelty of our study is in conducting an empirical test of these hypotheses, as most of previous studies are either anecdotal or only correlational and limited in their scope. We provide a cross-cultural test, using appropriate methodology, of these long-standing, but not systematically tested, hypotheses. We believe that the revised Introduction makes it sufficiently clear.

THEORY

The treatment of missing data on celibacy as absence needs prominent highlighting as a limitation earlier in the paper. The authors will be aware of recent critiques and retractions of papers based on cross cultural coding that make this mistake, and they need a very strong defence of this practice.

We are aware of the case of mistreatment of missing data and have provided a paragraph discussing this limitation in the Discussion.

Regarding the theoretical framing, we think the term ‘institution’ is being used too generally to refer to behaviors that may not require 3rd party norm enforcement (which is how we understand institutions generally work). While the authors would certainly not be the first to contribute to this ambiguity, it weakens their theoretical explanation for the behavior of interest. In a most extreme case, if celibacy were an institution reproduced by different norm-enforcement mechanisms (e.g., punishment, conformity), you wouldn’t need to invoke inclusive fitness to explain it. It’s certainly possible that celibacy has collective benefits that have led to the norms promoting this behavior being culturally selected at the group level. However, this isn’t something that the study argues, nor does it present evidence for such a case. The study instead argues that the distribution of celibacy across groups is due to a convergent adaptive response to ecological variation (e.g., in the form of sibling competition for parental resources), but it remains unclear why celibacy would not

be more widespread within the groups that practice it, if all its members are exposed to similar "ecological drivers". Granted, the data may be limited in this regard, but a similar question can raised regarding this behavior not being more frequent across groups. For example, why is religious celibacy "rare outside Eurasia", despite same-sex competition for parental resources or mates, and despite the widespread presence of major world religions? While the study does present a partial answer to this question in the form of sex-specific celibacy, it is still applying individual or household-level explanations to account for population-level phenomena or data. Thus, the question of why some groups have religious celibates and others (presumably) don't remains unclear. We don't view this as a fatal flaw to the theory, but we think the authors' line of argumentation needs to be more clearly defended against this position.

We indeed used the term institution in its general form but to avoid potential ambiguities we dropped it altogether.

The point raised by reviewers about why celibacy is not more widespread both within and across groups is a relevant one. We assume that individuals are – *on average* – exposed to similar pressures. Although, as you point out, our data is limited in its resolution – it is in fact at the level of an administrative unit (village, community, province, etc.) – it was carefully chosen to represent society as a whole, to reflect predominate patterns in society. Although such coarseness is certainly a limitation, it is necessary for this kind of analysis. Therefore, while we are fully aware of this limitation, we believe that the discrepancy between household/village/community and population level perspectives may not be serious enough to invalidate our explanatory framework or preclude us from making statements of this kind. Even at the cost of eventually noisier estimates.

We believe similar logic applies to the frequency of celibacy across groups. Celibacy is likely only one among multiple alternative strategies that mitigate competition for resources between siblings. These can include practices related to marriage, e.g., monogamy/polyandry, or inheritance, e.g., primogeniture/ultimogeniture, and we speculate that even infanticide is potentially one of such strategies. These practices often co-occur so they can sufficiently compensate for the need to have celibacy. In other instances, competition for parental resources can be present but not intense enough to adopt celibacy. For example, there are stark differences in the amount and nature of bride-price (a predictor of male celibacy in our study) between societies, which might explain why some bride-price societies have celibacy while others do not. We devote substantial part of the Discussion to these counter examples.

We are also a little confused by the introduction of Trivers-Willard (lines 146-151). First the authors don't really report this correctly – albeit this is how many subsequent papers

capture their hypothesis; also, Trivers and Willard were not talking about human families as implied in current text. Trivers and Willard's argument was that parents will invest in the sex with the higher returns to their investment, and that this may be males or females depending on the breeding system (Silk 1988) and the wealth/status of the parent. Critically these returns may switch across wealth levels, ultimately because of the structure of the mating system. For example, if polygyny is permissible, sons may be a better target for reproduction only among the wealthiest families. Once you recognize that TW uses individual-level logic, any predictions regarding stratification, marriage systems and inheritance (as in this paper) must include interaction effects. This was a critique that Dickemann makes of Hartung (in the cited 1982 paper). This concern in no way invalidates the analysis conducted in this paper (there is a place for population level analyses) but introducing T&W theory is probably a mistake.

Thank you for highlighting this. We used the TW hypothesis to explain examples of hypergyny and primogeniture in the early modern Europe referred to in the Introduction, not to directly derive predictions about celibacy. Therefore, the revised version of the Introduction no longer includes the TW hypothesis.

Despite a relatively clear statement of expectations in the abstract, and a general set of claims that resource conflict within (or across [see ambiguity below]) same-sexed individuals might drive celibacy, there is no clear exposition of exactly how and why this might be mediated. The reader is presented with a mass of studies (see Structure and Presentation) on correlations among a suite of traits (inequality, bridewealth, dowry, primogeniture, polygyny, inheritance, etc), but no clear causal framework is developed until we see the DAG. This DAG is not explained in the text, but I found the material in the Methods section at the end much more informative than any of the earlier material (see below). Claims like (line 146) the link between wealth and primogeniture is "not a coincidence" could be dropped, if we had a clear working model from the start of the paper, and an explanation of how this model is linked to the ecological factors heralded in the first line of the paper.

Thank you for this important point. We agree that the causal framework including the DAG itself should have been presented earlier in the text. Therefore, we decided to include the DAG in the Introduction, which simultaneously helps to lay the logic of our predictions.

There is no distinction between same-sex sibling competition and same sex competition more generally, even though the dynamics could be very different. For example, same-sex sibling competition might be expected to be sensitive to the inheritance system in a way that same-sex competition across the population might not. Similarly, the two types of

competition might play out very differently in stratified versus non-stratified societies – in the former it's feasible not all men would be in direct competition with each other if there is any economic endogamy. Again, I doubt this is a fatal flaw to the theoretical model advanced here, but it needs more thought, especially insofar as in some places the authors are clearly only thinking about sibling competition (viz line 331) and in other places they are ambivalent.

We acknowledge that the two terms were used interchangeably in the text but agree that same-sex sibling competition and same sex competition can have quite different dynamics. From a theoretical point of view of this paper, it is same-sex sibling competition that is of interest to us. On this note, all instances now refer to same-sex sibling competition.

The logic of the predictions regarding polygyny escaped me. We think the authors are assuming that same sex sibling competition will be lowest among females in polygynous societies, and lowest among males in monogamous societies (which seems reasonable). But we are not sure why they are predicting celibacy to be lowest in polygynous populations and highest in polyandrous population. It is certainly not true that there is no male-male competition (over wealth or wives) in polygynous populations. Some males are clearly excluded from marriage. Even more puzzling is why the authors seem to group polygynous and polyandrous societies (Figure 3). Again, a clear statement of the theoretical logic underlying predictions for each sex would help avoid these ambiguities (see directly below).

We used this ordering (polygyny-monogamy-polyandry) only in the analysis of general celibacy, as we believe it best reflects an increasing competition for heritable wealth - particularly land, which is at the heart of our causal model. Decline of polygyny and the rise of monogamy have been linked to the increasing importance of rival wealth (land), see Ross et al. (2018), while polyandry typically occurs under strong environmental constraints and severe competition for land/inheritance. Thus, we find this ordering justified. Moreover, it is argued in the Discussion that there indeed is competition for wealth in polygynous societies but it is less intense and the form of wealth is different (livestock). Despite a widespread distribution of polygynous marriage around the globe, celibacy is indeed rare in such societies, compared to monogamous or polyandrous ones. Please see the revised version of our Discussion for more details.

Regarding the grouping of polygyny with polyandry – we recoded this into polygyny vs. monogamy and polyandry and reanalyzed the data (male celibacy), as it was also suggested by another reviewer.

While we strongly support the use of DAGs to represent hypotheses and analytical strategy, it would probably be clearer to have one for each hypothesis. It feels as if the authors have thrown everything into the DAG without equal attention to how each element is theoretically relevant for each sex. Here are our more specific concerns:

(i) The role of a group's phylogeny or religion (the mechanisms of these forces) is not clear, and there is no clear description of the causal model further below (L497). You expect celibacy to vary across major religions, and argue these 'cause' celibacy, but why? Earlier you said that celibacy is present in almost all major religions, but what varies is the sex that is celibate. Even if you used this DAG for that slightly more specific hypothesis (religion -> celibate sex), what does religion causally have to do with this? Is the underlying assumption that all variation in same-sex resource competition is structured along religious boundaries? This would be crucial to state, but would still raise the question why not every sibling from the competing sex who is religious becomes a monk or a nun.

(ii) There seems to be discrepancy between the DAG and the supporting text in the methods section 498-524. The text argues for 3 proxies for resource competition – marriage transactions (bridewealth and dowry, # concurrent spouses, and sex specific inheritance) but if you look at the DAG these variables are included in quite distinct roles (as both causes and consequences of their unmeasured "Same sex (sibling) competition").

(iii) I would like the authors to consider why there is no arrow in the DAG between marital composition (e.g. polygyny) and marriage transactions (e.g. bridewealth), especially given Hartung's study linking bridewealth to polygyny (where notably (for the anomaly noted above #3) the posited link doesn't go through sibling competition but population-wide competition among men).

(iv) Also questionable in the DAG is the specification that phylogeny constrains unmeasured cultural factors, but not any of the other variables like marriage systems or marriage payments.

i) We included both phylogeny and religion for several reasons. We assumed they lead to covariation in celibacy among related societies, violating the assumption of independence of data points. Phylogeny is also used as a proxy for shared but unobserved cultural variables that may confound relationships of interest. In other words, related societies are more likely to have similar social organization, norms, etc., which can have a confounding influence. We haven't measured these variables, but we can use phylogenetic relatedness as a crude proxy to account for these influences (see 'Deffner, D., Rohrer, J. M., & McElreath, R. (2022). A causal framework for cross-cultural generalizability. *Advances in Methods and Practices in Psychological Science*, *5*(3)').

As for religion, it is not a cause in its classical meaning, rather it represents a variable along the values of which celibacy and the relationships we are testing can vary. Religion is understood as an effect modifier, which is not strictly confounding but it needs to be controlled for (see 'Pretelli I, Borgerhoff Mulder M, Makame Khamis B, McElreath R. 2023 Foraging and the importance of knowledge in Pemba, Tanzania: implications for childhood evolution. Proc. R. Soc. B 290: 20231505' for a similar case).

ii) Thank you for pointing this out. After careful consideration, we slightly revised our DAG and reanalyzed some of the models (those for the effect of marriage transactions) accordingly. We believe that the current DAG makes more intuitive sense and aligns more with the literature.

iii) Again, thank you for this comment. We agree, and we have included a causal arrow from marital composition to marriage transactions and reanalyzed some of the models correspondingly.

iv) You are right, we have not made links to other variables only for the simplicity of our DAG.

The relative importance of religion and phylogeny (with the former generally being a stronger predictor) is an interesting finding. It's strange that this is not set up as a theoretical objective of the study. I don't believe the work was preregistered, so it would seem legitimate to treat this as a feature rather than a bug.

Thank you for your suggestion to include it as one of the predictions. After consideration, we decided to keep it as it was originally.

STRUCTURE & PRESENTATION
Regarding the abstract, although a prediction is made "Here we predict that institutional lifelong religious celibacy will arise in societies where there is sibling competition for parental resources for marriage" a specification of all 3 hypotheses, and of the precise data to be used to address these hypotheses is needed. The sentence "Monogamy, heritable wealth and significant transfers of resources at marriage have all been shown to co-evolve across cultures (Mace, 2018)" is something of a distraction at this stage.

Thank you, following your advice, we specified the tested hypotheses and the data in Abstract. It is now more concise (to align with *Nat Comms* formatting standards) but contains all the relevant information about our predictions, the data and analysis.

The whole section of the history and geography of celibacy is interesting but is almost entirely devoid of any of the theoretical framing or evolutionary logic promised in the Abstract. I would suggest EITHER introduce clear hypotheses first and then frame earlier

findings around hypotheses, OR rewrite closely linking the literature review to the DAG (as currently in methods). I think latter option would be best, helping to reveal the logic for the DAG, which is not explained at all in the main text. A suitable introduction would distil a few central questions from the ethnography/history/previous research, and then pose a novel theoretical claim, with a convincing statement on how the current research improves on/complements/extends previous work. Clear statements of hypotheses must be made. In fact it is not until line 193-196 that we get a clear statement of hypotheses – this should come much earlier. A sentence like (128-130) "Scholars have suggested that constraints concerning land inheritance and partibility give rise to same-sex sibling competition and therefore celibacy (Hager 1992; Dickemann 1979; Betzig 1995) could, for example, be framed within theory (not just "scholars suggest"), and be placed at the outset of the section on sex-specific celibacy.

Thank you for your suggestion. We have substantially revised the Introduction where we reduced the number of ethnographic/historical examples and those remaining now serve to prepare the ground for the DAG, which has also been included in the Introduction. The section about the history and geography of celibacy has been moved to Supplementary Materials.

The Discussion is rather dense. First, there is a detailed discussion of examples (eg 367 to 392) that purport to explain some of the religion-specific variability, but it is very hard for a non-specialist in the area to assess this evidence, specifically how selectively chosen. Some more general claims about support (and failures to support) the overall model might be more digestible for advancing theory. Second by pinning claims to the simulation results in Figure 5 there seems to be quite a lot of redundancy with material covered in the Introduction. I think this could be improved by addressing concerns in #1 above. In fact Figure 5 is very hard to interpret, explained only as "When simulating the distributions of new outcomes based on the estimated parameters…". We appreciate there is material in methods (L614-626) but the rationale for these simulations needs a stronger set up in the main text. Also why is this in Discussion rather than in Results? It suggests the result is somehow not central to the story being told here. Generally, both reviewers were deeply puzzled here.

Thank you for this point. We acknowledge that the previous version of the Discussion contained numerous ethnographic/historical examples without a clear connection to the results. We have substantially reworked the Discussion, which we believe is now more general and balanced.

Regarding the simulation results, we have moved them from the Discussion to the Results under the section "Simulating the distributions of celibacy in Buddhist and Christian societies" and provided a brief rationale in the first paragraph of that section.

Finally, at line 428 we start getting some clearer prose that explains the results within a more comprehensive theoretical framework and points out where discrepancies occur and

why. Indeed, the paragraph ending on line 477 is clearly written, and more explicitly linked to the theory that appears to underlie the paper (but, as noted above, is not clearly flagged at the outset).

Thank you.

SMALLER POINTS

Figure 1. What is the source of these data? Do they all refer to contemporary period or ethnographic present? How generally do authors deal with contemporaneous and non contemporaneous data.

The data used for the figure is from eHRAF online database. These are our own data that we used in our analysis. We have added this information in the figure caption. The data refers largely to ethnographic present. Despite the bias due to ethnographic present is a persistent issue in cross-cultural research, our own work and other studies (see 'Ringen, E. J., Duda, P., & Jaeggi, A. V. (2019). The evolution of daily food sharing: A Bayesian phylogenetic analysis. *Evolution and Human Behavior*, *40*(4), 375-384') have shown that it is a relatively minor source of variation. Phylogenetic relatedness is much more important. Therefore, on these grounds, we did not account for the effect of ethnographic present in this study. Note also that we already used two sources of variation – phylogeny and religion. Yet another source of variation could result in overparameterization of our models, particularly in sex-specific celibacy with quite small sample sizes.

L85-88. Logic of argument unclear. More rules less status?

Correct, though this section is no longer present in the revised Introduction.

L113. "elsewhere"? Really everywhere in the world not mentioned in paragraphs above? Suggest authors reword.

Since the Introduction has been substantially revised this seems to be no longer relevant.

L187. Haddix presumably refers to (Haddix 2001).

Indeed, we have corrected it.

Figure 4. Do we assume panels left to right are again transactions, marriage structures, and wealth inheritance

Yes, the order is the same as in other panels but this information is not relevant compared to what kind of information was meant to be displayed here – variation components. Therefore, we did not specify which model exactly is being displayed. Also, the figure has been moved to the supplementary materials.

Results general. While I appreciate there are a lot of results to cover the presentation gets very sketchy about direction and actual findings. First, associations between variables are cryptically described as positive or negative, rather than spelling out that there is more (or less) celibacy with bridewealth, or polygyny. It's hard for the reader to remember whether dowry is coded 1 or 2, and also hard to remember whether positive or negative is in support of hypothesis or not. As a reader you find yourself glazing over, after having turned multiple times back to what the codings were. Could you make the language more friendly?

Thank you for highlighting this. Following your suggestion, the results have been completely rewritten in a more comprehensive manner.

Second, (this may be relevant as text lengthens due to addressing the point above), the diverse patterning of correlations between your predictors and outcomes vary across religions (266-302) – but this doesn't get much unpacked in the discussion; perhaps the results could go to the supplement?

We agree that the discussion of the variation across religions doesn't get much space, if we do, we discuss these issues from a more general perspective. But we were left with enough space after the revisions, so we decided to keep this section of the results. However, we have moved the figures showing this variation to the supplement.

L206: Since the study is not measuring how common celibacy is within a society, but rather between them, I would suggest more precise wording. For example: "Globally, the presence of lifelong celibacy is more common in societies where..."

We have rephrased our formulations as suggested.

Figure 2. All terms need description in figure legend. For example, what does wealth inheritance mean (sex biased? Primogeniture or not?), or marital composition (monogamy? polygyny? Polyandry?. Of course it becomes clear in the figures, but reader needs help through the DAG.

Thank you for this suggestion, we have added a comprehensive description of each variable in the legend.

L215: “Overall, competition for marriage partners, or lack thereof (i.e., marriage transactions and marital compositions; Table 1), exerts…”

The Results have been substantially revised, including this sentence.

L218 & L230: Since you’re measuring a group-level trait, change Male celibates and Female celibates to Male celibacy and Female celibacy. Change this also in Table 1, Figure 4, and the Data-Celibacy subsection (L542-548).

Thank you, we have changed it accordingly.

L234: Figure g-h???

Corrected to “Figure 3g-h”.

L238 / Table1: I really like the “% PP” column. Would it be useful to the reader to clarify that “expected side of zero” means ‘predicted directionality of effect’?

Certainly, we have added this explanation to the Methods.

L246 / Figure 3: I liked this figure and found it clear and easy to interpret.

Thank you.

L257-258: “…there is about two times more variation in the probability of celibacy”. Again, I would advise “…probability of a society having at least some celibate members”, or “…probability of finding celibacy in a society”, or similar. L260: “…religion accounts for … the probability of male celibacy being present” or “..having male (rather than female) celibates…”

Thank you, we rephrased these lines as suggested.

L322: “ecological drivers”. You have no ecological drivers in this analysis! In fact I found “ecological conditions” throughout the discussion, which should clearly be relabelled as social or institutional (insofar as I believe you are referring to marriage systems, inheritance customs, marriage payment transactions). Even socio-ecological seems like a poor use of

terminology. You are linking institutions (marriage and inheritance) to another institution (monasteries and nunneries).

Please, refer to our response to the use of the term in your comment above.

L325: "...reported by several case studies in contemporary populations" (sentence is too long to string adjectives for this noun I think)

The Discussion has also been substantially revised, as a result, this sentence was removed.

L409-11: "This is because female celibates are less common than male celibates and show a higher tendency to cluster around more closely related societies, contrary to male celibates, which are more widespread." This is not an explanation of the result – why is the female pattern more phylogenetically constrained – but a description of the patterning of the data

Thank you for highlighting this. We have reworked this section.

L451: Sentences like "female celibates are negatively associated with transactions from the bride's side" (Supplementary Figure 2c-3c)" are really indigestible for Discussion. Negatively associated with what transactions – again this just needs writing in clear English without assuming coding direction.

Thank you, we have substantially revised the Discussion including phrasing that is more appropriate for the Discussion.

L466: replace "be" with "are"

Thank you but this sentence is no longer relevant in the revised version of the Discussion.

L478-480: "Apparently maladaptive behaviours are often explained as a mismatch between ancestral and modern environments and as maladaptive by-product resulting from our tendency to conform, respectively (Boyd and Richerson 1985; Pinker 1997)." Boyd-Richerson and Pinker disagree radically on the potential sources of maladaptation. This sentence needs careful thought and rewriting.

Thank you for this point. We have rephrased the sentence.

L502-503: “Central to our causal model is the same-sex (sibling) competition for resources…”

Corrected.

L545-546: I’m a little confused about how you coded presence of sex-specific celibacy. How did you code societies that have both male and female celibacy? Figure 1 seems to suggest this is a separate binary outcome variable (1, presence of both male and female celibacy; 0, absence of both male and female celibacy). However, here you say you “created a binary indicator for the predominance of male celibates, e.g., monks, including societies having male celibates only as well as societies having both male and female celibates”. If you indeed lumped these two categories, why did you? If you did not, please re-phrase for clarity.

Yes, we did lump these two categories together in our analysis. It is because substantial portion of male celibacy occurs in association with female celibacy and vice versa. Societies with both are more common than societies with either form of celibacy in our sample. At the downside, we cannot completely separate male celibacy from female celibacy, which could be responsible for some noisy estimates in our results.

L551-554. I found no clear explanation for why polygyny is combined with polyandry as a predictor of male celibacy. All of the empirical studies reviewed point to the interaction of wealth differences and marriage rules.

We agree and, in line with the same point raised by another reviewer, we have recoded this predictor into polygyny vs. monogamy/polyandry and reanalyzed the data.

L558: The term ‘substantial’ suggests a threshold has been crossed. I would prefer it if you used the terms 'relatively greater' and ‘relatively fewer’ resources (transferred at marriage) instead.

We have changed it accordingly.

References

Clutton-Brock, T. (1991). The evolution of parental care. Princeton, Princeton University Press.
Downey, D. B. (2001). "Number of siblings and intellectual development: the resource dilution explanation." American Psychologist 56: 497-504.

Fontenay, S., et al. (2024). Fertility in sub-Saharan Africa: The role of inheritance, ULB--Universite Libre de Bruxelles.
Haddix, K. (2001). "When polyandry falls apart: Leaving your wife and your brothers." Evolution and Human Behavior 22(1): 47-60.
Lazarus, J. and I. R. Inglis (1986). "Shared and unshared parental investment, parent-offspring conflict and brood size." Animal Behavior 34(6): 1791–1804.
Ross, C. T., et al. (2018). "Greater wealth inequality, less polygyny: rethinking the polygyny threshold model." Journal of the Royal Society Interface 15(144).
Silk, J. B. (1988). "Maternal investment in captive bonnet macaques (Macaca radiata)." The American Naturalist 132(1): 1-19.

Reviewer #4 (Remarks to the Author):

The article addresses an important yet understudied topic. It is original and well done. I endorse its publication after a minor revision. I have some reservations about the analysis, but I do not believe that the results will change dramatically when methods that I would prefer are used. The only serious objection I have is to using a constant offset between polygamy (1)-monogamy (2) and monogamy (2)-polyandry (3) in the model of celibacy probability—in essence, to using simple bM*marriage in the model formula (more info below).

Petr Tureček

Thank you for your constructive and helpful review, especially regarding our analysis. While we decided to keep the original model formula, we have also implemented the analysis as suggested by the reviewer. Namely, to model marital composition as an ordinal variable. Although the results indeed do not change, additional analysis extends our understanding and strengthens our interpretations.

Also note that after a substantial revision of the manuscript, and taking into account other reviewers' suggestions, some of the comments posed by the reviewer may no longer be relevant or the corresponding sections that a given comment refers to have simply been moved to the supplementary materials.

More important points:

Pages 3–4: The construction "Where rules of female Buddhist monastic ordination can be found [..] they significantly outnumber the corresponding male collection of rules,

signifying the unequal status of male and female celibates [...] while [elsewhere] nuns are more common and respected" suggests (although only "unequal" is stated explicitly) that lower status is linked to more rules. This is possible, but in the Hindu tradition, for instance, higher castes typically need to observe more rules. Similar trends can also be found in Judaism, where the ability to follow religious rules more strictly is connected to higher social status and prestige. Can the authors elaborate on this? If they simply want to point out that a different number of regulations suggests a difference in status, that is fine, but they should then clarify why they do not want to make any claims about the direction of the inequality. However, given their expertise, I believe they are capable of making an informed claim about who holds lower status in this case and why is difference in status reflected in Buddhist monastic tradition in this particular way.

Thank you for your point. The section was meant to merely show that there are indeed differences in status between male and female celibates in Buddhism, and that a higher number of rules for female celibates reflects their lower status compared to male celibates. We believe that comparisons with Hinduism and Judaism may not be completely appropriate as these religions have very little celibacy at all. If they do, it is only one sex (males) but not both.

Nonetheless, this section is no longer present in the Introduction. It has been moved to the Supplementary Materials.

Page 4: The authors note that "there was no male equivalent [of calibate 'aclla' women] among the Inca." This is especially interesting given Inca are famous for great reproductive inequality among men. Ruling class Inca's – with Sapa Inca on top – were entitled to marry many more wives than commoners. Can authors, perhaps, return explicitly to the Inca case in the discussion? Why did not they develop male celibate if the competition between males was so big? Is it because they did not practice monetary transactions at marriage?

Thank you for bringing this to our attention. We have included a short paragraph discussing this example in the Discussion.

Page 6 and elsewhere: When the authors mention the Trivers-Willard hypothesis, could they take a moment to discuss why the effect is not sufficient in humans to compensate for hypergamy? Do high-status women really have more male children (excluding and including selective abortions and infanticide)? If not, why not? How solid is the Trivers-Willard effect among other animals?

Thank you for these interesting points but, following other reviewers' comments, we decided to remove the discussion of hypergyny (and primogeniture) to which the Trivers-

Willard hypothesis originally referred to in the Introduction.

Page 6, bottom: Why does monogamy lead to asymmetry in competition for marriage partners (less strict for men, more strict for women)? Is it because it is easier to establish a cultural norm that allocates rival wealth to one of the brothers deterministically (e.g., primogeniture), while all women, in effect, compete for these firstborn men?

Both polygyny and monogamy lead to competition for marriage partners, but as you say, what differs is the sex that competes more under respective marriage types. It is a result of multiple factors, such as subsistence and labor contributions to it by the sexes, prevailing marriage payments (dowry), and so on, rather than of easiness of establishing inheritance norms. Primogeniture/ultimogeniture, for example, are common also among polygynous societies.

Page 7: In the DAG, I find the absence of subsistence strategy somewhat surprising. Is it omitted because no causal path would require adding, for instance, a contrast between intensive agriculture and other subsistence strategies in the model? The paper mentions causal paths from agriculture/pastoralism to cultural norms in several places, so its omission here seems odd. Otherwise, it should be made explicit that this DAG is more a part of the analytical strategy than a claim of exclusive causality between data variables. For instance, wealth inheritance can definitely impact social stratification. The causality is not unidirectional. (But a model with cycles cannot be expressed as a DAG.)

Correct. From our experience, although subsistence has certainly a manifold influence, it is often an exogenous variable, being linked to the outcome only indirectly. Although subsistence is mentioned as part of our argument, there is no explicit causal hypothesis in the literature linking agriculture to celibacy. Even if such hypotheses existed, it would likely be treated as a proxy for material resources, which is already implemented in our DAG.

Figure 5: The effects on this scale seem rather small. What do you think is the reason for this, given that the hypothesis is so sound and the presented anecdotes so convincing? It would be helpful to add a paragraph about this in the discussion.

Thank you for highlighting this. There are several reasons behind this. Firstly, celibacy (as defined in the paper) is relatively uncommon globally and that may be why some of the effects appear small (e.g., marriage transactions on celibacy in the global sample). For this reason, we decided to analyze celibacy in the Eurasian subsample as well as across religions where we see stronger effects.

The second reason may be the way we coded sex-specific celibacy where both male celibacy and female celibacy variables included societies that have both male and female celibates, not only either male or female celibacy (see the Data section in the Methods). This could introduce noise in our estimates.

Another reason could be that celibacy is only one among several alternative strategies that mitigate competition for heritable wealth (monogamy, primogeniture, or perhaps even infanticide). These strategies often co-occur, which could compensate for the need to adopt celibacy. This could explain why we do not see celibacy in socio-ecological contexts where our models/predictions would expect it.

We have added a paragraph addressing these issues in the Discussion.

Page 10: The authors state that the effect of marriage transactions is positive in Christian (90% PP) and Buddhist (55% PP) societies, while it is negative in all others. Perhaps the authors should acknowledge that these effects form a continuum, with the Buddhist slope being right on the edge. A 49% PP would be interpreted as "negative," correct? This suggests that Christian societies may be the exception in this respect.

You're right. We adjusted these interpretations accordingly; please see the second and the last paragraphs in the section "Evaluating the variation accounted for by the phylogeny and religion" on p. 13-14.

Page 15: Overall, the most salient result is that the more competition between relatives, the more celibacy can be expected in the population, because "societies that have both are most frequent in [your] sample." Do you share this opinion? If so, could you elaborate on it in the discussion?

Indeed, competition for resources among both sexes can occur in a single society and it can explain why most of celibate societies have celibates of both sexes. We provide a short discussion of this issue in the Discussion.

Page 15: The authors state that "…it is adaptive for parents to allocate resources to a smaller number of offspring…" While this may sometimes be true, primogeniture can still evolve when firstborns can assert their interests—since it is always more favorable to them than to their parents, as they are twice as related to their own children as any other relatives are. Many narratives around primogeniture emphasize parental agency while overlooking the role of direct sibling competition in shaping societal norms. In fact, parents are incentivized not to split resources among offspring only if reproductive returns increase

with the amount of rival resources that must be divided. This can occur, for example, when agricultural success depends on a relatively small amount of arable land, but many other circumstances can produce similar resource-reproduction relationships. A reflection on these issues would be appreciated. See also Ross, C. T., Borgerhoff Mulder, M., Oh, S. Y., Bowles, S., Beheim, B., Bunce, J., ... & Ziker, J. (2018). Greater wealth inequality, less polygyny: Rethinking the polygyny threshold model. Journal of the Royal Society Interface, 15(144), 20180035, for a related discussion on rival resources, polygyny, and environmental carrying capacity.

Thank you for bringing this to our attention. Our theoretical perspective emphasizes the predominant role of parents, specifically parent-offspring conflict, in driving celibacy, but we agree that the interests of other parties, e.g., firstborn sons, also play a role and might have been overlooked. Still, we think these two perspectives are not mutually exclusive and both are perfectly aligned with inclusive fitness theory. We find the recent publication by the reviewer and colleagues – that we believe these points come from – inspiring and incorporated it in our Discussion.

Page 16: The authors write that the depletion of fitness can be achieved "...by sending away some offspring to become celibate monks or nuns." It would be interesting to address how far celibate offspring are typically sent, as findings on celibate monks increasing parental inclusive fitness suggest that celibate siblings become net contributors to family resources. To do so, they would need to live in reasonable proximity. On the other hand, if primogeniture leads to the large-scale dispersal of later-borns (such as when younger sons are sent to the military), their direct reproduction does not necessarily have to be restricted—they may contribute to parental inclusive fitness through direct reproduction rather than by supporting nephews and nieces. All in all, celibacy seems to function as an alternative to actual dispersal. Do the authors agree? If so (or even if not), could they reflect on this in the article? Otherwise, this issue will likely keep resurfacing.

We certainly agree and have included a brief reflection of these issues in the Discussion.

Page 16: The authors note that "However, while low-status males were usually sent to beggar castes and, more rarely, to monasteries, e.g., in Tamil India, male monastic institutions are predominantly found in areas with the highest rates of groom-wealth (Slater 1918), females are not associated with any form of religious celibacy." This contradicts the predictions of the authors' hypothesis, which is fine (as counterexamples are expected given that the distribution of outcomes follows the patterns in Figure 5). Nevertheless, this

raises an interesting question: what do the extra unmarried women in this society do? Could the authors elaborate?

Thank you for this point. Following also other reviewers' advice, a significant part of the Discussion is now devoted to counter-examples, meaning cases where our models would expect celibacy, yet it is absent there. It also includes this particular example, which we explain by the presence of female infanticide as alternative to celibacy.

Supplement, Page 19: What are the authors' thoughts on the existence of societies where assigning children to celibate social roles does not prevent them from inheriting (and thus potentially depleting) portions of family wealth? This is particularly relevant given the interesting example where "sons and daughters who become monks or nuns receive their share of the property at the time they take monastic vows..."

Thank you for bringing this up. Despite being interesting, as far as we know, such cases are rare and, therefore, we do not give it much special attention. One example that could fit in with this description – at least in our sample - were Babylonians. Here, king's daughters were appointed as priestesses and received dowries at the time of entering convents. They may be allowed to marry but were forbidden from bearing children, which were bored by priestesses' sisters or concubines. Interestingly, children born in those marriages did not inherit this wealth. Instead, it returned back to the paternal lineage, thus not depleting family wealth.

Methods:
The description of the statistical analysis in its current form does not clearly convey what was precisely done. I had to examine the model code to understand the details. Sharing the models in the form of equations—perhaps in the paper's supplement—would be much appreciated so that other readers do not have to search through the code. Precisely describing the models in the methods section is also necessary.

We added the mathematical definitions of our models; please see p. 33 in the Supplementary Materials.

Regarding what was done, I have several comments:
1. The marital composition is described as a "3-level ordinal scale" in the models of celibacy. However, from the code, it appears to be a variable with values 1, 2, or 3 that is simply multiplied by a slope bM. This approach imposes equal spacing (on the log-odds scale) between polygyny and monogamy, as well as between monogamy and polyandry. In my opinion, this is not the correct way to incorporate an ordered-categorical predictor into

a logistic regression.
The authors should instead capture the effect with two parameters: one representing the total difference between the lowest and highest competition categories (polygyny and polyandry) and another determining the relative position of monogamy between them, which could be closer to either polygyny or polyandry. (See, for instance, how education is incorporated in Model 12.6 of Statistical Rethinking, 2nd edition, which the authors reference.)
Alternatively, they could use the approach employed in their models where celibacy presence is investigated by sex, using two binary indicators. This would allow the polyandry level to emerge as the sum of two (likely positive) parameters, naturally yielding the highest celibacy baseline.
Which of these two models is preferable should be treated as an empirical question, potentially decided by predictive out-of-sample fit measures such as WAIC.

Thank you for pointing this out. We decided to use the first option and included the analysis of marital composition as ordered predictor. As per the suggestion of another reviewer, we also analyzed it as a categorical predictor. See the Results section for details on these analyses.

2. This “3-level ordinal scale” variable is also not standardized or transformed in any way, meaning that the intercept of the model now corresponds to the nonexistent value 0 on this scale. Am I correct? This seems unfortunate. (The extremely wide compatibility interval for celibacy probability in polyandrous societies in Figure 3B may also be a result of this choice.)
While you are probably right, we hope that our additional analysis - modeling marital composition as ordinal - offsets this. It shows that polyandry increases celibacy, particularly in the Eurasian subsample (see Results and Supplementary Figures 1 and 4).

3. The analysis description should explicitly state that varying intercepts and slopes are used per religion and that uncorrelated varying effects are assumed. The current version of the manuscript only mentions “varying effects” in the “Analysis” section, which is too vague.

Thank you, we have added that religion effects are assumed to be uncorrelated.

I would also consider modeling intercorrelated intercepts and slopes per religion, as, for instance, a religion with a high baseline probability of “monks” cannot be expected to show such large effects of marital transactions on this probability. However, given the number of categories in the religion variable, the precision of estimates may not improve drastically,

and the compatibility interval for the correlation itself would likely be very wide. It is up to the authors to decide whether this refinement is worth their time.

We agree that modeling the correlation between intercepts and slopes could 'soak up' part of unexpectedly large effects, but given quite small sample sizes of some subsamples, we prefer to keep our models relatively 'simple'. And while we are aware of the advantages of partial pooling (between intercepts and slopes when modeling their correlation), keep in mind that our models already include two random effects as well as phylogeny, essentially another random effect. More importantly, we believe it would not have changed the observed patterns, namely that of the prominence of Buddhism and Christianity.

4. The authors claim to use "weakly informative" priors, yet all the priors are unbiased (centered around 0). Do you mean "weakly regularizing"? Or am I misunderstanding the terminology? Weakly informative typically implies a prior that nudges estimates toward a particular direction, such as a positive or negative slope, or toward a smaller or larger intercept than 0.5.

You're right, we meant regularizing priors, which has now been corrected.

5. Treating "indigenous" as an equivalent category to Buddhism or Christianity can be misleading. For instance, the current models assume potentially constant variance within Buddhist and within "none of the above" societies. Is this intentional? Is there a way to address it, such as adding an additional dispersion parameter for the "indigenous" category? While I believe this would not change the main results (which are primarily drawn from Christian and Buddhist societies), acknowledging this limitation in the models would be helpful.

We understand your concern. However, while the indigenous category groups all other religions that are not counted as major world religions, celibacy across this category is almost invariably absent. Although it could potentially improve precision, we believe that estimates for this category, which are relatively consistent across our models (see Supplementary Tables 2-7), would not change. Also, as you pointed out – the results are primarily driven by Buddhism and Christianity. Nonetheless, we have acknowledged this in the Methods sections (p. 21).

6. I have reservations about how phylogeny is incorporated into the models. Currently, the distance matrix is simply multiplied by an (estimated) number. This effectively implements a Brownian motion model, which does not distinguish between the similarity of closely related cultures and the rate at which similarity decays with linguistic distance. Is there a specific reason for using this model (beyond the fact that the single sigma can be

conveniently plotted against the standard deviations of by-religion intercepts and slopes in Figure 4)? Have the authors considered using an Ornstein-Uhlenbeck (exponential, cov_GPL1 in rethinking) kernel (this would be probably my preference) or a quadratic (cov_GPL2) alternative? These approaches would allow for a better estimation of the effect of relatedness between observations.

Thank you for highlighting this. As you point out – the main reason we used this approach, instead of implementing a specific function to model how covariance decays with phylogenetic distance (suggested OU kernels), is that we aimed for a simple and convenient method of comparing (co)variation components - phylogeny and religion - in our models. But note that instead of a distance matrix we used a correlation matrix, which was multiplied by sigma parameter giving overall magnitude of phylogenetic correlations. We followed the standard PGLMM approach as outlined in these papers:

Ives, A. R., & Helmus, M. R. (2011). Generalized linear mixed models for phylogenetic analyses of community structure. *Ecological Monographs*, *81*(3), 511-525.

Gallinat, A. S., & Pearse, W. D. (2021). Phylogenetic generalized linear mixed modeling presents novel opportunities for eco-evolutionary synthesis. *Oikos*, *130*(5), 669-679.

7. In the analysis, the authors state that they used “software available at the ASJP website to calculate a pairwise distance matrix between languages” and then constructed a tree, while the model essentially uses an inverse of a distance matrix. Doesn’t forcing languages into a tree structure—when their relationships may resemble a network rather than a series of bifurcations—result in information loss? Would it not be better to omit this unnecessary step and use the original language similarity matrix directly?

Yes, we certainly agree that the ‘two-step’ process we utilized could potentially lead to some loss of information. However, we wanted to include societies that speak the same language or a dialect of a single language in the sample and the most straightforward way to do so was to add them directly on the tree – as polytomies (see lines 27-65 in the code file no. 1). Surely, we could simply duplicate given wordlists in an input file for the ASJP software but – as mentioned in our response above - what entered the models was a correlation (not a distance) matrix, which can be readily obtained from the tree. We are not aware of an R function that could do a simple conversion of phylogenetic distances to correlations.

Technical details:

Page 4: The sentence “However, while in Tibetan and Theravāda Buddhism, the main form

of Buddhism in Sri Lanka, Myanmar, Laos, Cambodia, and Thailand, nuns are rare compared to monks, in Vietnam, China, Korea, and Japan, nuns are more common and respected (Kieschnick 2007; Powers 2007; Micheletti et al. 2022)." is long and not easy to parse.
A bracket would improve readability. How about:
"However, while in Tibetan and Theravāda Buddhism (the main form of Buddhism in Sri Lanka, Myanmar, Laos, Cambodia, and Thailand), nuns are rare compared to monks, in Vietnam, China, Korea, and Japan, nuns are more common and respected (Kieschnick 2007; Powers 2007; Micheletti et al. 2022)."
Thank you, it has been rephrased.

Page 6: I do not understand the sentence: 'For example, a farm cannot be divided Haddix.' Is this a typo or an incomplete citation?

This was an incomplete citation, which has been corrected.

Page 6-7 and elsewhere: the authors should more clearly define the difference between social stratification and wealth inequality. Throughout the manuscript, these terms seem to be used interchangeably, though they are not the same (as some usages in the manuscript also suggest). Wouldn't it make sense to include both in the DAG in Figure 2?

We agree that they are not the same. We simply used social stratification as a proxy for wealth inequality, as there are no measures of it for societies in either D-PLACE or eHRAF. See other studies for examples of a similar treatment of wealth inequality:

Ross, C. T., Strimling, P., Ericksen, K. P., Lindenfors, P., & Mulder, M. B. (2016). The origins and maintenance of female genital modification across Africa. *Human Nature*, *27*(2), 173-200.

Haynie, H. J., Kavanagh, P. H., Jordan, F. M., Ember, C. R., Gray, R. D., Greenhill, S. J., ... & Gavin, M. C. (2021). Pathways to social inequality. *Evolutionary Human Sciences*, *3*.

Page 7: I suggest moving the DAG closer to the methods section. As it stands, it feels somewhat disconnected, leaving the reader zigzagging between the methods and the figure when reading about the models.

Thank you. We have rewritten the Introduction to accommodate causal inference, including the logic of our DAG.

Figure 3: These figures seem ugly to me. Why are the posterior mean circles so large if their

size has no meaning? Adding a grid behind each panel would be helpful. The 'PP xx.xx %' text is also too big and sometimes overlaps the bars.

We have adjusted the sizes of circles, so it doesn't collide with the text.

Page 20: I advise rehosting the code and data on a platform other than GitHub. While GitHub is great for version control, the final product should be shared on a platform that allows generating permanent links (DOIs).

We have rehosted the data to a figshare platform with the DOI provided in the manuscript.

Reviewer #4 (Remarks on code availability):
The code is understandable. It contains a README file (although typesetted in a very large font - like if everything is a headline, why?). As a suggest above, please rehost to a platform that allows generating DOI for the files.

It has been rehosted to figshare, including the modified README file.

REVIEWER COMMENTS

Reviewer #1 (Remarks to the Author):

I am reviewing this paper for the second time after the authors completed major revisions. This paper, always interesting, is much improved overall—and I now think that it can be accepted pending minor revisions. Specifically, the introduction is much clearer and more streamlined, major concerns raised by reviewers in terms of the analysis have been addressed, and the discussion is now organized, well-written, and robust.

Below I include a list of mostly minor, though a few more significant, comments that I suggest be addressed prior to publication. Among these, the only significant concern is with regard to acknowledgment that the analysis is not a formal causal analysis, and tweaking some of the language in acknowledgement of this.

Thank you again for the positive feedback on our manuscript. We have followed the reviewer's advice on the use of causal language and have rephrased the text as suggested.

p. 2
The authors write that "but this is more than outweighed by an indirect fitness benefit obtained by increasing the competitiveness of their other offspring."
This statement needs to be qualified. I suggest "is theoretically more than outweighed…" or "potentially more than outweighed….".

We have changed it as suggested.

The authors write that "This, in turn, enhances the reproductive fitness of the monk's brothers,"
Again, this needs to be qualified. I suggest "This, in turn, IS EXEPECTED TO enhance the reproductive fitness of the monk's brothers OR THE NUN'S SISTERS,"

Changed as suggested.

The authors write "generated by marriage patterns". I recommend "generated IN PART by marriage patterns".

Changed as suggested.

Define "rival" in paragraph 2.

We have added a definition of rival resources.

The authors write “a lack of marriage opportunities in daughters”
This should be “among daughters”.

Reworded.

The authors write that “In societies where marriage customarily involves wealth transfers from the bride’s side of the family to the groom’s (groom-wealth 35) or to the couple or the bride (‘dowry’36)”
While I fully agree that this is a meaningful distinction theoretically, this distinction is not commonly made in the literature or among people in such cultures. It’s also common that dowries may serve both as groom wealth and endowment in the same societies (e.g. most of modern South Asia). Given that the authors only use one variable—dowry—to cover these kinds of transactions in their analysis, it does not make sense to me to set this up to read as if they will separate groom wealth and dowry here.

Thank you for highlighting this. We have removed the mention of groom wealth from the text.

p. 3
The authors write that “While classical studies of celibacy relied on case studies and were largely anecdotal or correlational, this work is novel in providing a systematic and cross-cultural test of these associations.”
The work in this paper is also correlational (though a more robust version) so I am not sure I would emphasize this critique of other work.

We have removed this part of the sentence and rephrased the rest.

The authors write that “With respect to marital composition, we expect that celibacy is more likely to occur in societies where that number is equal”
The phrase “that number is equal” is not clear; I suggest updated wording.

It has been reworded for better clarity.

The authors write that “However, given that matriliny is significantly less common than patriliny and inheritance in matrilineal societies is commonly passed down to nephews (i.e., diagonally), we do not expect matrilineal inheritance to play a role in driving female celibacy and thus leave this prediction out from testing.”
Is this left out due to low expectations, or because it is very rare so you just don’t have data to test it? Given that the response to reviewers implies the latter, I suggest just saying that it’s because there aren’t enough cases to test this.

In fact, it is both in our view. We left it out because of low expectations and even if we wanted to test it, there wouldn’t be enough data to do so. Thus, we consider the

statement accurate.

p. 7 Results
The authors write that “globally, marriage transactions—either from the groom’s side, bride’s side or both sides—increased the likelihood of lifelong celibacy but the effect was weak”
This causal-sounding language is not appropriate as the analyses presented (while appropriate) are not causal. It would be clearer to say that marriage transactions are “associated with an increased likelihood of celibacy.”

We have rephrased as suggested.

p. 9
In Figure 3, panels a and c should be shifted to a 0-1 scale on the y axis as is already true for panels d-h. Though I realize you are trying to have them match with panel b, it is very difficult to see the effect on the 0-2 scale.

We have changed the scale in Fig 3a-3c and in Fig S3a-S3c to 0-1 to align them with the rest of the panels.

p. 10
The authors write that “We found that Buddhism and, to a similar but lesser degree, Christianity exhibited the strongest support for our predictions, in terms of both magnitude and directionality of effects.”
“To a similar but lesser degree” is not clear; saying it this way makes it sound like the magnitudes are similar. I suggest “Buddhism and, similarly but to a lesser degree, Christianity…”

Rephrased.

p. 11
The authors write:
“male celibacy was mostly driven by marriage transactions and marital composition,”
“female celibacy was driven mostly by marriage transactions though it was generally less prominent.”
Again, I suggest altering causal-sounding language.

We have rephrased these sentences to tone down causal language.

p.15, Discussion
See concerns about causal language below, and take these into account when editing the discussion. Generally, though, this section is really strong.

We have toned down the causal language in the Discussion and throughout the paper.

p. 17
The authors write “while the results proved to serve as a baseline for subsequent sex specific analyses.”
What does ‘proved’ mean here? I suggest rewording to clarify.

Rephrased.

The authors write that “Lifelong religious celibacy, an apparently maladaptive behavior, is often explained as the result of an evolutionary mismatch or as a by-product of adaptation.”
These alternative explanations have not been discussed in the paper, even in the introduction where they are only reviewed in a single sentence at the very beginning of the paper. It is thus odd to mention it here. I suggest citing and clarifying these perspectives in the introduction before referring to them here.

We have added more detail in the Introduction and slightly reworked the Conclusions.

The authors write “and causal inference methods.”
Again, the analyses presented are not causal. I would change this language.

We have replaced this with “…models formally adjusted for a set of potential confounding variables.”

p. 18 Methods

Regarding the “Causal model” section and related issues:
The use of a DAG does not mean that the analyses produce causal inferences. While a DAG hypothesizes a causal model, no testing here is being done that would actually allow causality to be ascertained. I would thus steer away from causal language as many readers will find it inappropriate even if they find your methods are appropriate.

Related to this, while I agree that the DAG hypothesized is appropriate for this paper and analysis, it hypothesizes a particular set of relationships among variables—but this is not the only way that the variables could potentially be related. For example, it is far more likely that marriage transactions and patterns of wealth inheritance are co-evolved with celibacy under conditions of competition for limited resources than that one actually ‘causes’ the other.

In light of both of these concerns, I recommend the addition of a few sentences to the methods section or the SI directly addressing these concerns about causality.

We fully agree with the reviewer's points on this matter and have acknowledged the limitations of the DAG approach in the Methods.

The authors write that "Firstly, we assumed that marriage transactions are a direct cause of resource competition" is very appropriately worded with the use of "assumed". I suggest that similar terms are used in the following points:

• "Secondly, marital composition, defined as a socially recognized number of concurrent spouses for a given sex, i.e., polygyny, monogamy or polyandry, IS ASSUMED TO [OR, ALTERNATIVELY, IS HYPOTHESIZED TO] influence resource competition indirectly through marriage transactions"

• "Thirdly, patterns of wealth inheritance are ASSUMED TO BE another cause of resource competition"

We have added the terms as suggested.

The authors write "involve relatively fewer amounts of resources"
This should be "involve fewer resources" or "involve smaller amounts of resources"

Rephrased.

The authors write "take other form"
Should be "take another form"

Rephrased.

P. 20
The authors write "was used as proxy for wealth inequality"
This should be "as a proxy for wealth inequality" as the verb 'was' was already used at the beginning of the sentence.

Rephrased.

I was also asked by the editorial team to comment specifically as to whether the revision has been responsive to Reviewer 2, who was no longer available. I also evaluated their responses to Reviewer 4.

After carefully reviewing the comments made by reviewers 2 and 3 (in a joint review) and 4, I still think that this manuscript can be accepted pending minor revisions—or given a decision of minor revisions if that is the norm at Nature Communications. Generally speaking, the main concerns of the other reviewers have been thoughtfully addressed, either in terms of changes to the text of the manuscript or in terms of

appropriate responses to the comments in the rebuttal. It should be noted especially that this manuscript was heavily revised (major revisions), including significant rewriting of the introduction and discussion, in response to the first round of review, thus not all previous comments are relevant to the current manuscript.

Below I outline a few minor issues which one could argue were not fully addressed by the authors, but none of these seems particularly serious to me—thus the recommendation for minor revisions.

Reviewers 2 and 3
The authors do not address the issue of including the inclusion of ecology in the text, but they have dealt with this comment and responded in the rebuttal.

R2/3 original comment: The treatment of missing data on celibacy as absence needs prominent highlighting as a limitation earlier in the paper. The authors will be aware of recent critiques and retractions of papers based on cross cultural coding that make this mistake, and they need a very strong defence of this practice.

Author response: We are aware of the case of mistreatment of missing data and have provided a paragraph discussing this limitation in the Discussion.

My comment: The authors have responded to this, but not in detail. I am fine with what they have done, but perhaps the other reviewers would want something more direct. Perhaps a slight enhancement of the current response.

We have improved the statement on our treatment of missing data (p. 17).

There is a concern about group level data and individual-level explanatory models. I think the authors have addressed this appropriately, and in fact I don't share the reviewers' concerns on this front since there is no reason to expect the two will be different in this case and no way to realistically use a different sort of data for this analysis.

We agree with reviewer no. 1.

R2/3 original comment: Figure 1. What is the source of these data? Do they all refer to contemporary period or ethnographic present? How generally do authors deal with contemporaneous and non contemporaneous data.

Author response: The data used for the figure is from eHRAF online database. These

are our own data that we used in our analysis. We have added this information in the figure caption. The data refers largely to ethnographic present. Despite the bias due to ethnographic present is a persistent issue in cross-cultural research, our own work and other studies (see 'Ringen, E., Duda, P., & Jaeggi, A. V. (2019). The evolution of daily food sharing: A Bayesian phylogenetic analysis. Evolution and Human Behavior, 40(4), 375-384') have shown that it is a relatively minor source of variation. Phylogenetic relatedness is much more important. Therefore, on these grounds, we did not account for the effect of ethnographic present in this study. Note also that we already used two sources of variation – phylogeny and religion. Yet another source of variation could result in overparameterization of our models, particularly in sex-specific celibacy with quite small sample sizes.

My comment: The point about the ethnographic present should be included in the manuscript (I do not see it there) or at least in the supplement.

We have included a statement regarding ethnographic present and its potential effect on recording celibacy in the limitations section of the Discussion (p. 17).

Reviewer 4

R4 original comment: 2. This "3-level ordinal scale" variable is also not standardized or transformed in any way, meaning that the intercept of the model now corresponds to the nonexistent value 0 on this scale. Am I correct? This seems unfortunate. (The extremely wide compatibility interval for celibacy probability in polyandrous societies in Figure 3B may also be a result of this choice.)

Author response: While you are probably right, we hope that our additional analysis - modeling marital composition as ordinal - offsets this. It shows that polyandry increases celibacy, particularly in the Eurasian subsample (see Results and Supplementary Figures 1 and 4).

My comment: This has not been dealt with directly in the text, but it does not seem like a concern to me in the current version of the manuscript.

We have dealt with this concern as suggested by reviewer 4.

There are numerous statistical topics raised by R4. Generally speaking, the authors have either addressed these (~65% of the time) or explained their reasons for not doing so (~35%). Most of this I find thoughtful and reasonable, though there are a few comments that I am not enough of an expert to evaluate. I am not concerned with this

paper moving forward, but a statistical specialist should be asked if there is concern from the editors.

Reviewer #4 (Remarks to the Author):

I see most of my concerns adequately addressed in the discussion and supplement.

What I do not understand is, however, why the central analysis (if I read the current version correctly) remained the one that treats variable “marriage” as continuous (values 1-3, implying also intercept being estimated for a contrafactual 0 value). Clearly, polyandry is not “twice as far” from polygyny than monogamy as the model with categorical predictor suggests. I find Figure 3 (b) presented as the main result misleading given the findings from ordinal model (that, however, cannot capture the possibility of celibacy probability decreasing again from monogamy to polyandry!) and the categorical model. I would strongly recommend making the categorical model the main one and omit Fig. 3b altogether or place it in the supplement (preferably after changing the continuous variable to -1 – 0 – 1, where 0 is monogamy… But still. Even such a model would be misleading and clearly dominated by the categorical model. Am I not right? What would the model comparison using WAIC, PSIS or similar suggest?)

The fact that we kept the original model treating marital composition as continuous variable was a result of misunderstanding of the reviewer’s point on our side. We have now dropped this model altogether from the paper and present the results of ordinal and categorical models only. We’ve also replaced the original continuous model with categorical model in the panel in Figure 3 and Figure S3, as suggested by the reviewer.

Otherwise, when zigzagging between the main text and supplement, I discovered a typo:
“Similarly, in Hindu societies, male celibacy aligns with our predictions (Supplementary Figures 2b–7b)” Supplementary Figure 2 is now the single-panel categorical model plot without.

Thank you for pointing this out. It was likely an artifact resulting from revising the manuscript.

Reviewer #4 (Remarks on code availability):

Data and code are available and allow to reproduce the results.

REVIEWERS' COMMENTS

Reviewer #1 (Remarks to the Author):

I have reviewed this article twice before, and the authors have now addressed all of my comments. I recommend that the paper be accepted and published.

Reviewer #4 (Remarks to the Author):

I am pleased to see that the manuscript has been revised according to my suggestions.

I would, however, now strongly suggest to plot a different y limit in panels a-c in Figure 3. All the "action" is happenning below 0.2 or so and the reader can now see almost no difference between the groups as the y-limit reaches all the way to 1. If authors worry that readers might be confused about different y-limits across panels, they can highlight the difference (and the fact that the models can still consider the full 0-1 probability scale) in the figure's caption.

Different y-limits have been plotted for Fig 3 and Supplementary Fig 3 and the readers are notified about this change in the captions.

I have no further comments. Congratulations on a fine paper!

Reviewer #4 (Remarks on code availability):

The code is fine.
